# Domino Aza-Michael-S_N_Ar-Heteroaromatization Route to C5-Substituted 1-Alkyl-1*H*-Indole-3-Carboxylic Esters

**DOI:** 10.3390/molecules27206998

**Published:** 2022-10-18

**Authors:** Ebenezer Ametsetor, Spencer Farthing, Richard A. Bunce

**Affiliations:** Department of Chemistry, Oklahoma State University, Stillwater, OK 74078-3071, USA

**Keywords:** domino reaction, 1*H*-indole synthesis, aza–Michael reaction, S_N_Ar reaction, heteroaromatization

## Abstract

A new synthesis of C5-substituted 1-alkyl-1*H*-indole-3-carboxylic esters is reported. A series of methyl 2-arylacrylate aza-Michael acceptors were prepared with aromatic substitution to activate them towards S_N_Ar reaction. Subsequent reaction with a series of primary amines generated the title compounds. Initially, the sequence was expected to produce indoline products, but oxidative heteroaromatization intervened to generate the indoles. The reaction proceeded under anhydrous conditions in DMF at 23–90 °C using equimolar quantities of the acrylate and the amine with 2 equiv. of K_2_CO_3_ to give 61–92% of the indole products. The reaction involves an aza-Michael addition, followed by S_N_Ar ring closure and heteroaromatization. Since the reactions were run under nitrogen, the final oxidation to the indole likely results from reaction with dissolved oxygen in the DMF. Substrates incorporating a 2-arylacrylonitrile proved too reactive to prepare using our protocol. The synthesis of the reaction substrates, their relative reactivities, and mechanistic details of the conversion are discussed.

## 1. Introduction

Indoles are among the most widely distributed heterocycles in nature and many have critical functions in living organisms. Due to their potent biological profiles, numerous natural and synthetic indoles have been prepared and studied by chemists to mitigate the effects of various diseases [1,2]. To date, numerous synthetic approaches have been developed and this family of compounds remains a highly active area of research in organic and medicinal chemistry.

The major “named” synthetic routes to indoles have been nicely summarized in the review cited above [1]. Other less general methods include: (1) intramolecular cyclization of a side chain amine on a neighboring benzyne triple bond [3], (2) cyclization of a benzylic anion to an ortho-substituted isocyanide [4], (3) intramolecular addition of a nitrene to a styryl double bond [5], and (4) palladium catalyzed ring closure of 2-iodo-1-allylaminobenzenes [6] among other variations [7]. Beyond direct syntheses, indoles can also be accessed from indolines through dehydrogenation [8,9] or elimination of functionality on the five-membered ring [10]. Finally, once prepared, methods for the introduction of alkyl substitution to the indole nitrogen have been reported by a number of routes [11,12,13,14,15].

The current study sought to develop a new synthetic approach to these systems through a domino aza-Michael-S_N_Ar-heteroaromatization sequence from acrylate esters substituted at C2 by an aromatic system substituted to promote nucleophilic aromatic substitution by an addition-elimination (S_N_Ar) mechanism. Previously, indole derivatives have been prepared by a domino-reduction-reductive amination reaction from 2-nitrophenylacetone [16] and a domino reduction-aza-Michael-elimination process from ethyl 2-(2-nitrophenyl)-b-ketoesters [17]. These procedures differ from the current route in aromatizing via elimination of water from the initial adduct. The present study involves (**1**) aza-Michael addition to a polarized 2-arylacrylate double bond, (**2**) ring formation by S_N_Ar of the added nitrogen to the electron-deficient aromatic ring, and (**3**) aromatization by reaction with molecular oxygen which is either dissolved in the reaction solvent or admitted to the flask during removal of TLC samples. The conversion is clean and provides the indoles in high yield.

**Figure 1:**
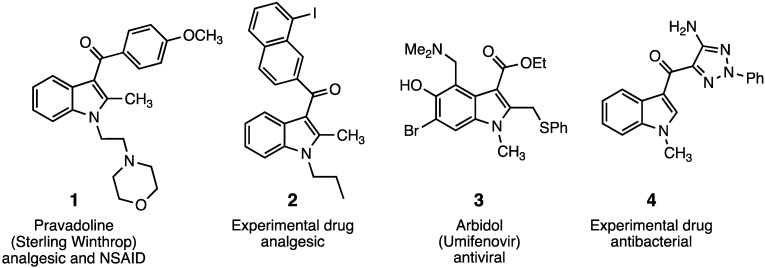
Indole-containing drug compounds incorporating the *N*-alkyl and 3-acyl groups.

Indoles are prevalent in many drug compounds showing a wide range of activities. Filtering for compounds that possess similar structural features to the compounds prepared here–an alkylated nitrogen at position 1 and an acyl group at C3–revealed a number of structures that are pictured in Figure 1. Pravadoline (**1**) has potent analgesic properties via binding to the cannabinoid CB_1_/CB_2_ receptors [18] and is closely related to neuroprotective compounds that inhibit inflammation caused by b-amyloid proteins involved in Alzheimer’s disease [19]. The iodinated naphthoylindole **2** is a strong analgesic as it also binds to the CB_1_/CB_2_ receptors [20]. Arbidol (Umifenovir, **3**) is a potent antiviral [21], used primarily in Russia and China, against influenza A [22]. Finally, 3-indolyl-5-amino-2-phenyl-1,2,3-triazine **4** has shown highly promising antimicrobial activity towards both Gram positive and Gram negative bacteria [23].

## 2. Results and Discussion

**Scheme 1:**
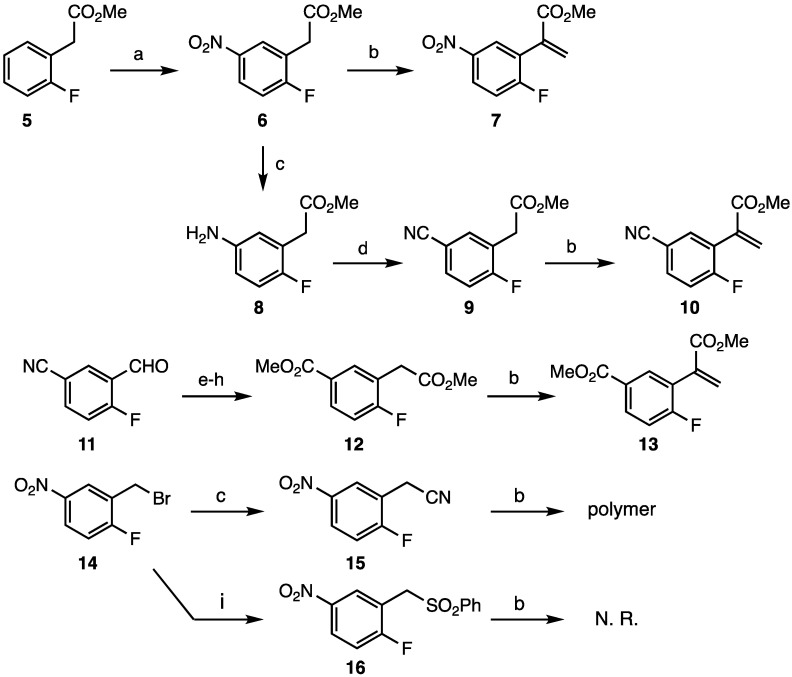
Synthesis of the reaction substrates to prepare methyl 1-alkyl-1*H*-indole-3-carboxylates. Key: (a) NaNO2, H2SO4, 0–23 °C, 91%; (b) 37% aq. HCHO, K2CO3, DMF, 23 °C, 58-62%; (c) Fe, NH4Cl, aq. EtOH, 85 °C, 93%; (d) HONO, CuCN, 65%; (e) NaBH4, EtOH, 23 °C, 91%; (f) PBr3, Et2O, 0–23 °C 90%; (g) KCN, aq. EtOH, 23 °C, 76-88%; (h) 25% H2SO4, MeOH, 90 °C, 60%; (i) PhSO2Na, EtOH, 78 °C, 70%.

The 2-arylacrylate indole precursors **7**, **10** and **13** were prepared using standard techniques (Scheme 1). Methyl 2-fluorophenylacetate (**5**) was converted to methyl 2-(2-fluoro-5-nitrophenyl)acrylate (**6**) by nitration using NaNO_3_ in H_2_SO_4_ at 0–23 °C for 2 h [24]. Installation of the acrylate double bond to give **7** was accomplished by aldol condensation with formaldehyde (37% aq. formaldehyde (formalin), K_2_CO_3_, DMF, 23 °C) [25]. The 2-(5-cyano-2-fluorophenyl)acrylate substrate (**10**) was prepared from the aforementioned intermediate nitration product **6**. Reduction of the nitro group to give aniline **8** (Fe/NH_4_Cl, aq. EtOH, 70 °C) [26] was followed by diazotization (HONO) and Sandmeyer replacement of nitrogen by cyanide (CuCN) [27,28] to afford **9**. Final conversion to acrylate **10** was accomplished as above. Finally, the diester substituted substrate **13** was prepared from 5-cyano-2-fluorobenzaldehyde (**11**). Reduction of the aldehyde to the benzyl alcohol (NaBH_4_, EtOH, 23 ^o^C), conversion to the bromide (PBr_3_, Et_2_O, 0–23 °C) [29], S_N_2 displacement of bromide by cyanide (KCN, aq. EtOH) and methanolysis of the dicyano compound (25% H_2_SO_4_ in MeOH, 90 °C) generated diester **12**. Aldol condensation with formaldehyde then led to **13**. Yields were reasonable for all steps and each synthesis was performed on a multigram scale. It should be noted that attempts to install the aza-Michael accepting double bond in 2-(2-fluoro-5-nitrophenyl)acetonitrile (**15**), generated from 2-fluoro-5-nitrobenzyl bromide (**14**) [30], yielded polymeric material under the aldol conditions used and 1-fluoro-4-nitro-2-((phenylsulfonyl)methyl)benzene (**16**), prepared from this same bromide [31], failed to aldolize under the conditions used.

Our cyclization results are summarized in Table 1. The reaction was carried out in anhydrous DMF using 1 mmol of the acrylate, 1 mmol of the RNH_2_ and 2 mmol of K_2_CO_3_. Primary amines incorporating a primary, secondary or tertiary alkyl group were all successful in the reaction but anilines failed to initiate the sequence due to their diminished reactivity. We also found that hydrazine reacted with nitro activated substrate **7** but not the cyano and ester activated substrates **10** and **13**, respectively [32]. Despite the a-effect which increases the nucleophilicity of hydrazine [33], the less S_N_Ar active substrates **10** and **13** primarily afforded products resulting from reaction at the pendant ester and cyano groups. As expected, the five-membered ring was entropically favored over the six-membered ring in the reaction of hydrazine with **7**. Additionally, the five-membered ring also benefited from stabilization gained via heteroaromatization.

Substrates incorporating nitro and cyano activation on the S_N_Ar acceptor ring proceeded at room temperature while the ester–bearing substrate required heating up to 90 °C. This observation likely reflects the relative activating ability of the different groups in the S_N_Ar process. In all cases, the work–up required adding the crude reaction mixture to aq. NH_4_Cl, extracting with ether, and washing the combined organic layers with aq. NaCl. The ether layers were dried and concentrated to give a crude product that was purified by column chromatography. All products exhibited spectral and analytical data in accord with the assigned structures (see Supplementary Materials).

**Scheme 2:**
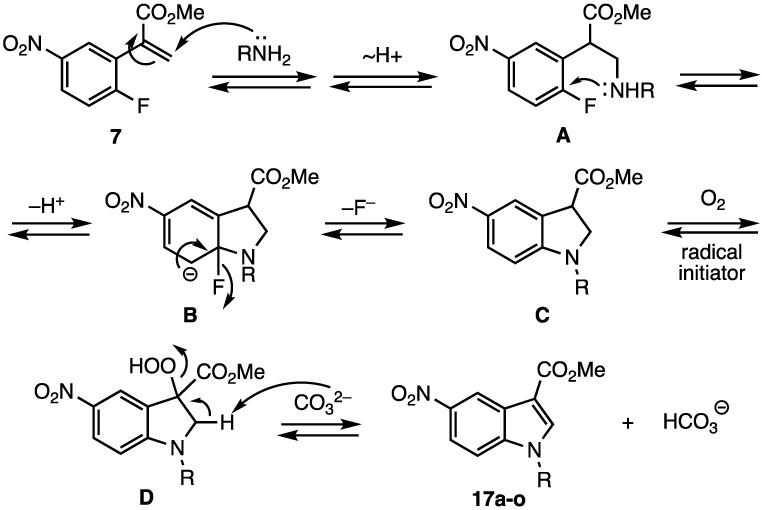
Plausible mechanism for the domino aza-Michael-S_N_Ar-heteroaromatization of **7**.

The exact chronology of events in the reaction sequence is unknown, but a plausible sequence is outlined for substrate **7** in Scheme 2. Due to the low temperatures employed, the initiating step was assumed to involve aza–Michael addition of the amine to the unhindered 2-arylacrylate double bond followed by loss of a proton to give amine adduct **A**. The nitrogen in this intermediate is positioned to add to the activated aromatic ring by a S_N_Ar reaction via Meisenheimer intermediate **B** to give indoline **C**. The heteroaromatization process likely occurs due to exposure of the compound to dissolved oxygen in the DMF [34] or oxygen introduced during removal of TLC samples. Since indoline **C** has a highly activated benzylic C–H substituted by an ester group, insertion of oxygen at this site to form a peroxide intermediate **D** should be facile. This process often requires a radical initiator [32], but it is unclear what could perform this function in the current reaction. Once oxygen inserts into the activated C–H bond, elimination under the basic conditions would install the double bond to afford the indole **17** (Scheme 2). In no case was indoline **C** detected during the reaction or in the final product. A similar process was hypothesized for the formation of quinolones in an earlier study [35].

## 3. Materials and Methods

### 3.1. General Methods

Unless otherwise indicated, all reactions were performed under dry N_2_ in oven-dried glassware. All reagents and solvents were used as received. All wash solutions in work-up procedures were aqueous. Reactions were monitored by thin layer chromatography on Analtech No 21521 silica gel GF plates (Newark, DE, USA). Preparative separations were performed by flash chromatography on silica gel (Davisil^®^, grade 62, 60–200 mesh) containing 0.5% of UV-05 UV-active phosphor (both from Sorbent Technologies, Norcross, GA, USA) slurry packed into quartz columns. Band elution for all chromatographic separations was monitored using a hand-held UV lamp (Fisher Scientific, Pittsburgh, PA, USA). Melting points were obtained using a MEL-TEMP apparatus (Cambridge, MA, USA) and are uncorrected. FT-IR spectra (0.09 cm^−1^ resolution between 4000–500 cm^−1^) were run as thin films on NaCl disks using a Nicolet iS50 spectrophotometer (Madison, WI, USA). ^1^H- and ^13^C-NMR spectra were measured using a Bruker Avance 400 system (Billerica, MA, USA) at 400 MHz and 101 MHz, respectively, in the indicated solvents containing 0.05% (CH_3_)_4_Si as the internal standard; coupling constants (*J*) are given in Hz. Low-resolution mass spectra were obtained using a Hewlett-Packard Model 1800A GCD GC-MS system (Palo Alto, CA, USA). Elemental analyses (±0.4%) were determined by Atlantic Microlabs (Norcross, GA, USA).

### 3.2. Methyl 2-(2-Fluoro-5-nitrophenyl)acetate (***6***)

The procedure was modified from that of Gale and Wilshire [24]. Methyl 2-(2-fluorophenyl)acetate (**5**, 10.0 g, 59.5 mmol), was added drop-wise over 1 h to an ice-cooled solution of sodium nitrate (5.60 g, 65.8 mmol) in concentrated sulfuric acid (102 g, 55.3 mL). After a further 30 min at 5–10 °C, the solution was poured onto ice and the resulting mixture was extracted with ether (3 × 50 mL). The combined ether layers were washed with water (2 × 50 mL), saturated NaHCO_3_ (1 × 50 mL), and saturated NaCl (1 × 50 mL) and then dried (Na_2_SO_4_). Filtration and concentration under vacuum gave the crude nitrated product as a yellow solid that was purified by trituration with 2% ether in pentane to give **6** (11.5 g, 91%) as a light yellow solid, m.p. 51–53 °C. IR: 1741, 1527, 1350 cm^−1^; ^1^H NMR (400 MHz, CDCl_3_): δ 8.25–8.18 (complex, 2H), 7.23 (t, *J* = 8.8 Hz, 1H), 3.78 (d, *J* = 1.4 Hz, 2H), 3.75 (s, 3H); ^13^C NMR (101 MHz, CDCl_3_): δ 169.8, 164.6 (d, *J* = 258.1 Hz), 144.2, 127.6 (d, *J* = 5.9 Hz), 125.2 (d, *J* = 10.1 Hz), 123.2 (d, *J* = 18.3 Hz), 116.4 (d, *J* = 24.6 Hz), 52.6, 34.1 (d, *J* = 2.6 Hz); MS (*m/z*) 213 (M^+^); Anal. Calcd for C_9_H_8_FNO_4_: C, 50.71; H, 3.78; N, 6.57. Found: C, 50.77; H, 3.83; N, 6.55.

### 3.3. Methyl 2-(5-Amino-2-fluorophenyl)acetate (***8***)

The procedure of Zhou and co-workers was used [26]. Nitroester **7** (5.00 g, 23.5 mmol) was dissolved in a mixture of EtOH (165 mL) and H_2_O (45 mL). The solution was heated at 70 °C under N_2_, NH_4_Cl (1.26 g, 23.5 mmol) and iron powder (3.92 g, 70.1 mmol) were cautiously added, and heating was continued for 2–3 h. The reaction mixture was filtered through celite, treated with saturated NaHCO_3_ (100 mL), and extracted with ethyl acetate (3 × 50 mL). The combined organic extracts were washed with saturated NaCl, dried (Na_2_SO_4_), filtered, and concentrated under vacuum to give amino ester **8** (4.00 g, 93%) as a light tan solid, m.p. 35-37 ^o^C. This material was spectroscopically pure and was used without further purification. IR: 3445, 3369, 3232, 1732 cm^−1^; ^1^H NMR (400 MHz, CDCl_3_): δ 6.84 (t, *J* = 8.9 Hz, 1H), 6.57–6.49 (complex, 2H), 3.70 (s, 3H), 3.58 (d, *J* = 1.4 Hz, 2H), 3.37 (br s, 2H); ^13^C NMR (101 MHz, CDCl_3_): δ 171.3, 154.5 (d, *J* = 236.6 Hz), 142.5 (d, *J* = 2.4 Hz), 121.7 (d, *J* = 17.2 Hz), 117.4 (d, *J* = 3.4 Hz), 115.8 (d, *J* = 23.1 Hz), 115.2 (d, *J* = 7.6 Hz), 52.2, 34.3 (d, *J* = 2.9 Hz); MS (*m/z*): 183 (M^+^).

### 3.4. Methyl 2-(5-Cyano-2-fluorophenyl)acetate (***9***)

The general procedure of Clarke and Read was used [27]. All water and aqueous solutions in this procedure used deionized H_2_O. CuCl was prepared from CuSO_4_·5H_2_O (3.42 g, 13.7 mmol), NaCl (0.90 g, 15.5 mmol) and Na_2_SO_3_ (from 0.72 g of NaHSO_3_/Na_2_S_2_O_5_ and 0.48 g (18 mmol) of NaOH) in H_2_O (14 mL) as described by Marvel and McElvain [28]. The CuCl was purified by decantation and suspended in H_2_O (10 mL). To the magnetically stirred suspension, a solution of NaCN (1.82 g, 37.1 mmol) in H_2_O (5 mL) was added drop-wise over 10 min and the CuCl dissolved with the generation of heat.

Aminoester **8** (2.00 g, 10.9 mmol) was mixed with crushed ice (ca 5 g) and 28% HCl (3 mL) was added. The flask was surrounded by an ice bath to maintain the temperature at 0–5 °C and a solution of NaNO_2_ (0.84 g, 12.1 mmol) in H_2_O (3 mL) was added with stirring over 15 min. This mixture was neutralized to pH 7 by slow addition of solid anhydrous Na_2_CO_3_ to give a solution of the diazonium salt.

The above CuCN solution was cooled to 0–5 °C, toluene (4 mL) was added and vigorous stirring was initiated. To this was added drop-wise the cold solution of the diazonium salt during 15–20 min keeping the temperature at 0–5 °C. N_2_ was evolved during the addition. The mixture was warmed to 23 °C over 2 h and then heated to 50 °C for 5 min. The heat was removed and the reaction was allowed to return to 23 °C over 1 h. The crude reaction mixture was transferred to a separatory funnel and the mixture was extracted with EtOAc (3 × 25 mL). The combined organic extracts were washed with saturated NaCl (3 × 50 mL), dried (Na_2_SO_4_), and concentrated under vacuum. The product from two runs at the above scale was purified by silica gel column chromatography (50 cm × 2.5 cm) eluted with 10–20% EtOAc in hexanes to give nitrile ester **9** (2.73 g, 65%) as a yellow solid, m.p. 51–53 °C. IR: 2232, 1741 cm^−1^; ^1^H NMR (400 MHz, CDCl_3_): δ 7.66–7.58 (complex, 2H), 7.19 (t, *J* = 8.8 Hz, 1H), 3.74 (s, 3H), 3.71 (d, *J* = 1.5 Hz, 2H); ^13^C NMR (101 MHz, CDCl_3_): δ 169.9, 163.4 (d, *J* = 256.8 Hz), 135.9 (d, *J* = 5.5 Hz), 133.6 (d, *J* = 9.6 Hz), 123.5 (d, *J* = 17.4 Hz), 117.9, 116.9 (d, *J* = 23.7 Hz), 108.7 (d, *J* = 4.0 Hz), 52.5, 33.8 (d, *J* = 2.9 Hz); MS (*m/z*): 193 (M^+^); Anal. Calcd for C_10_H_8_FNO_2_: C, 62.18; H, 4.17; N, 7.25. Found: C, 62.25; H, 4.19; N, 7.14.

### 3.5. Methyl (4-Carbomethoxy-2-fluorophenyl)acetate (***12***)

To a solution of the 5-cyano-2-fluorobenzaldehyde (**11**, 10.0 g, 67.1 mmol) in absolute ethanol (80 mL) at 0 °C (ice-water bath), NaBH_4_ (1.27 g, 33.5 mmol) was added portion-wise with stirring during 10–15 min. The cooling bath was removed and the reaction was allowed to warm to room temperature for 1 h. The reaction was quenched by addition to saturated NaCl (250 mL) and extracted with ether (3 × 50 mL). The combined ether layers were washed with saturated NaCl (3 × 50 mL) and dried (Na_2_SO_4_). Filtration and concentration under vacuum gave the alcohols as off-white solids that were purified by trituration with 2% ether in pentane to give (5-cyano-2-fluorophenyl)methanol (9.22 g, 91%) as an off-white solid, m.p. 48–50 °C; IR: 3429, 2234 cm^−1^; ^1^H NMR (400 MHz, CDCl_3_): δ 7.84 (dd, *J* = 6.7, 2.1 Hz, 1H), 7.61 (ddd, *J* = 8.3, 4.8, 2.1 Hz, 1H), 7.16 (t, *J* = 9.0 Hz, 1H), 4.80 (s, 2H), 2.16 (br s, 1H); ^13^C NMR (101 MHz, CDCl_3_): δ 162.5 (d, *J* = 256.6 Hz), 133.5 (d, *J* = 9.5 Hz), 133.1 (d, *J* = 6.2 Hz), 130.1 (d, *J* = 16.1 Hz), 118.4, 116.5 (d, *J* = 22.9 Hz), 108.6 (d, *J* = 3.9 Hz), 58.2 (d, *J* = 4.4 Hz); MS (*m/z*) 151 (M^+^); Anal. Calcd for C_8_H_6_FNO: C, 63.58; H, 4.00; N, 9.27. Found: C, 63.65; H, 3.97; N, 9.22.

A solution of (5-cyano-2-fluorophenyl)methanol (9.22 g, 61.1 mmol) in anhydrous ether (100 mL) was cooled to 0 °C. To the stirred solution was added drop-wise PBr_3_ (8.32 g, 2.89 mL, 30.7 mmol) during 1 h. The reaction was allowed to warm to 23 °C overnight at which time it was quenched by addition to saturated NaCl (150 mL). The ether layer was separated and the aqueous layer was washed with ether (2 × 50 mL). The combined ether layers were washed with saturated NaCl (3 × 50 mL) and dried (Na_2_SO_4_). Filtration and concentration under vacuum gave the bromide as an off-white solid which was purified by trituration with 2% ether in pentane to give 2-(bromomethyl)-4-cyano-1-fluorobenzene (11.8 g, 90%) as an off-white solid, m.p. 55–56 °C; IR: 2238 cm^−1^; ^1^H NMR (400 MHz, CDCl_3_): δ 7.76 (dd, *J* = 6.9, 2.2 Hz, 1H), 7.64 (ddd, *J* = 8.6, 4.8, 2.2 Hz, 1H), 7.20 (t, *J* = 8.9 Hz, 1H), 4.48 (s, 2H); ^13^C NMR (101 MHz, CDCl_3_): δ 162.9 (d, *J* = 260.0 Hz), 135.5, (d, *J* = 4.5 Hz), 134.6 (d, *J* = 9.7 Hz), 127.3 (d, *J* = 16.0 Hz), 117.5, 117.3 (d, *J* = 22.9 Hz), 109.1 (d, *J* = 4.0 Hz), 23.5 (d, *J* = 4.3 Hz); MS (*m/z*) 213, 215 (*ca* 1:1, M^+^); Anal. Calcd for C_8_H_5_FBrN: C, 44.89; H, 2.35; N, 6.54. Found: C, 44.97; H, 2.39; N, 6.44.

To a solution of KCN (5.16 g, 79.2 mmol) in water (6 mL) at 23 °C was added drop-wise a solution of the 2-(bromomethyl)-4-cyano-1-fluorobenzene (11.3 g, 52.8 mmol) in ethanol (75–90 mL) during 1 h. The solution was stirred for 16 h and quenched by addition to saturated NaCl (250 mL) and extracted with ether (3 × 50 mL). The combined ether layers were washed with saturated NaCl (3 × 50 mL) and dried (Na_2_SO_4_). Filtration and concentration under vacuum gave 3-(cyanomethyl)-4-fluorobenzonitrile as a light tan solid which was subjected to methanolysis without further purification.

A solution of concentrated sulfuric acid in methanol (100 mL, 25% *v*/*v*) was prepared at 0 °C. The benzylic nitrile was added slowly and the mixture was heated to boiling for 16 h (bath temperature 90 °C). After cooling, the reaction was quenched by addition to saturated NaCl (250 mL) and extracted with ether (3 × 50 mL). The combined ether layers were washed with saturated NaCl (3 × 50 mL) and dried (Na_2_SO_4_). Filtration and concentration under vacuum gave the ester as a yellow oil. Purification was accomplished by silica gel column chromatography (40 cm × 2.5 cm) eluted with 10–15% ether in hexanes to give 7.05 g (60%, 2 steps) of diester **12** as a light yellow oil. IR: 1735, 1720 cm^−1^; ^1^H NMR (400 MHz, CDCl_3_): δ 7.98 (m, 2H), 7.12 (t, *J* = 9.1 Hz, 1H), 3.91 (d, *J* = 2.6 Hz, 2H), 3.73 (s, 3H), 3.72 (s, 3H); ^13^C NMR (101 MHz, CDCl_3_): δ 170.6, 166.0, 164.0 (d, *J* = 254.4 Hz), 133.5 (d, *J* = 5.4 Hz), 131.1 (d, *J* = 9.5 Hz), 126.5 (d, *J* = 3.4 Hz), 121.8 (d, *J* = 16.9 Hz), 115.6 (d, *J* = 22.9 Hz), 53.3, 52.2, 34.2 (d, *J* = 2.7 Hz); MS (*m/z*) 226 (M^+^); Anal. Calcd for C_11_H_11_FO_4_: C, 58.41; H, 4.90. Found: C, 58.39; H, 4.94.

### 3.6. General Procedure for Conversion of the 2-Arylacetate Esters to Acrylates

The basic procedure of Selvakumar and co-workers was used [25]. To a mixture of the methyl 2-arylacetate (8.0 mmol) in formalin (37%, 18 mL) was added a suspension of anhydrous K_2_CO_3_ (1.66 g, 12.0 mmol) in DMF (5 mL). The resulting mixture was heated to 60 °C for 2 h and then cooled to 23 °C. The crude reaction mixture was quenched with water (75 mL) and extracted with ether (3 × 50 mL). The combined ether extracts were washed with saturated NaCl (3 × 50 mL) and dried (Na_2_SO_4_). Filtration and concentration under vacuum gave the crude product as a light yellow oil. Purification by silica gel column chromatography (25 cm × 2.5 cm) eluted with increasing concentrations of ether in hexanes afforded the pure acrylate esters, which solidified on standing.

#### 3.6.1. Methyl 2-(2-Fluoro-5-nitrophenyl)acrylate (**7**)

Yield: 1.97 g (60%) as an off-white solid, m.p. 52–54 °C; IR: 1730, 1634, 1527, 1350 cm^−1^; ^1^H NMR (400 MHz, CDCl_3_): δ 8.24 (m, 2H), 7.24 (t, *J* = 8.8 Hz, 1H), 6.68 (s, 1H), 6.03 (s, 1H), 3.83 (s, 3H); ^13^C NMR (101 MHz, CDCl_3_): δ 165.3 163.4 (d, *J* = 259.7 Hz), 144.1, 134.5, 131.6 (d, *J* = 1.7 Hz), 126.9 (d, *J* = 5.3 Hz), 126.4 (d, *J* = 17.4 Hz), 125.8 (d, *J* = 10.3 Hz), 116.6 (d, *J* = 24.9 Hz), 52.7; MS (*m/z*) 225 (M^+^); Anal. Calcd for C_10_H_8_FNO_4_: C, 53.34; H, 3.58; N, 6.22. Found: C, 53.37; H, 3.61; N, 6.13.

#### 3.6.2. Methyl 2-(5-Cyano-2-fluorophenyl)acrylate (**10**)

Yield: 1.66 g (58%) as a yellow solid, m.p. 69–70 °C; IR: 2231, 1723 cm^−1^; ^1^H NMR (400 MHz, CDCl_3_): δ 7.66 (ddd, *J* = 8.5, 4.7, 2.2 Hz, 1H), 7.63 (dd, *J* = 6.7, 2.2 Hz, 1H), 7.20 (dd, *J* = 9.2, 8.5 Hz, 1H), 6.64 (d, *J* = 0.8 Hz, 1H), 5.97 (d, *J* = 0.7 Hz, 1H), 3.82 (s, 3H); ^13^C NMR (101 MHz, CDCl_3_): δ 165.4, 162.3 (d, *J* = 258.4 Hz), 135.2 (d, *J* = 4.5 Hz), 134.5, 134.3 (d, *J* = 9.8 Hz), 131.3 (d, *J* = 1.6 Hz), 126.9 (d, *J* = 16.7 Hz), 117.8, 117.1 (d, *J* = 23.9 Hz), 108.6 (d, *J* = 4.0 Hz), 52.6; MS (*m/z*): 205 (M^+^); Anal. Calcd for C_11_H_8_FNO_2_: C, 64.39; H, 3.93; N, 6.83. Found: C, 64.33; H, 3.96; N, 6.78.

#### 3.6.3. Methyl 2-(5-Carbomethoxy-2-fluorophenyl)acrylate (**13**)

Yield: 2.03 g (62%) as an off-white solid, m.p. 52–54 °C; IR: 1724, 1627 cm^−1^; ^1^H NMR (400 MHz, CDCl_3_): δ 8.04 (ddd, *J* = 8.6, 5.0, 2.3 Hz, 1H), 8.00 (dd, *J* = 7.1, 2.3 Hz, 1H), 7.13 (t, *J* = 9.0 Hz, 1H), 6.58 (d, *J* = 1.1 Hz, 1H), 5.95 (d, *J* = 1.1 Hz, 1H), 3.92 (s, 3H), 3.81 (s, 3H); ^13^C NMR (101 MHz, CDCl_3_): δ 166.0, 165.9, 162.8 (d, *J* = 256.0 Hz), 135.7, 132.8(d, *J* = 4.7 Hz), 132.0 (d, *J* = 9.7 Hz), 130.3, 126.4 (d, *J* = 3.3 Hz), 125.4 (d, *J* = 16.0 Hz), 115.7 (d, *J* = 23.1 Hz), 52.5, 52.3; MS (*m/z*) 238 (M^+^); Anal. Calcd for C_12_H_11_FO_4_: C, 60.51; H, 4.65. Found: C, 60.48; H, 4.64.

### 3.7. General Procedure for Preparing C5-Substituted Methyl 1-Alkyl-1H-indole-3-carboxylates

A solution of the C5-substituted 2-arylacrylate (1 mmol) and the primary amine (1 mmol) in DMF (4 mL) was treated with anhydrous K_2_CO_3_ (276 mg, 2 mmol) and stirred for 12 h at 23 °C. At this time, TLC indicated that the reaction was complete. The crude reaction mixture was added to saturated NH_4_Cl (50 mL) and extracted with ether (3 × 25 mL). The combined organic extracts were washed with saturated NaCl (50 mL), dried (Na_2_SO_4_), filtered, and concentrated under vacuum. The crude product was purified by passing through a short silica gel column (25 × 2.5 cm) eluted with increasing concentrations of ether in hexanes. The compounds prepared are summarized below. *Notes*: (1) When 3-nitrobenzylamine hydrochloride was used as the amine, 3 equiv of K_2_CO_3_ were used. (2) When the substrate incorporated an ester activating group on the S_N_Ar acceptor ring, the reaction was stirred for 12 h at 50–90 °C as indicated in Table 1.

### 3.8. Reactions with Methyl 2-(2-Fluoro-5-nitrophenyl)acrylate (***7***) 

#### 3.8.1. Methyl 1-Hexyl-5-nitro-1*H*-indole-3-carboxylate (**17a**)

Yield: 243 mg (80%) as a light yellow solid, m.p. 52–54 °C; IR: 1707, 1612, 1537, 1341 cm^−1^; ^1^H NMR (400 MHz, CDCl_3_): δ 9.08 (d, *J* = 2.3 Hz, 1H), 8.18 (dd, *J* = 9.1, 2.3 Hz, 1H), 7.97 (s, 1H), 7.41 (d, *J* = 9.1 Hz, 1H), 4.19 (t, *J* = 7.1 Hz, 2H), 3.97 (s, 3H), 1.88 (quintet, *J* = 7.1 Hz, 2H), 1.31 (m, 6H), 0.88 (t, *J* = 6.9 Hz, 3H); ^13^C NMR (101 MHz, CDCl_3_): δ 164.5, 143.4, 139.2, 136.9, 125.9, 119.0, 118.3, 110.2, 109.5, 51.5, 47.5, 31.2, 29.9, 26.4, 22.4, 13.9; MS (*m/z*): 304 (M^+^); Anal. Calcd for C_16_H_20_N_2_O_4_: C, 63.14; H, 6.62; N, 9.20. Found: C, 63.11; H, 6.59; N, 9.14.

#### 3.8.2. Methyl 1-Isobutyl-5-nitro-1*H*-indole-3-carboxylate (**17b**)

Yield: 243 mg (88%) as a light yellow solid, m.p. 131–133 °C; IR: 1706, 1614, 1539, 1347 cm^−1^; ^1^H NMR (400 MHz, CDCl_3_): δ 9.06 (d, *J* = 2.3 Hz, 1H), 8.16 (dd, *J* = 9.1, 2.3 Hz, 1H), 7.96 (s, 1H), 7.40 (d, *J* = 9.1 Hz, 1H), 4.00 (d, *J* = 7.4 Hz, 2H), 3.96 (s, 3H), 2.21 (nonet, *J* = 6.9 Hz, 1H), 0.97 (d, *J* = 6.7 Hz, 6H); ^13^C NMR (101 MHz, CDCl_3_): δ 164.4, 143.3, 139.5, 137.5, 125.8, 119.8, 118.2, 110.4, 109.3, 55.0, 51.5, 29.5, 20.1; MS (*m/z*): 276 (M^+^); Anal. Calcd for C_14_H_16_N_2_O_4_: C, 60.86; H, 5.84; N, 10.14. Found: C, 60.83; H, 5.82; N, 10.17.

#### 3.8.3. Methyl 1-Allyl-5-nitro-1*H*-indole-3-carboxylate (**17c**)

Yield: 234 mg (90%) as a light yellow solid, m.p. 119–121 °C; IR: 1704, 1620, 1534, 1342 cm^−1^; ^1^H NMR (400 MHz, CDCl_3_): δ 9.06 (d, *J* = 2.3 Hz, 1H), 8.16 (dd, *J* = 9.1, 2.3 Hz, 1H), 7.96 (s, 1H), 7.40 (d, *J* = 9.1 Hz, 1H), 6.01 (ddt, *J* = 17.0, 10.6, 5.4 Hz, 1H), 5.35 (d, *J* = 10.6 Hz, 1H), 5.18 (d, *J* = 17.0 Hz, 1H), 4.82 (d, *J* = 5.4 Hz, 2H), 3.96 (s, 3H); ^13^C NMR (101 MHz, CDCl_3_): δ 164.3, 143.5, 139.3, 137.0, 131.2, 126.0, 119.4, 118.8, 118.4, 110.4, 109.7, 51.5, 49.8; MS (*m/z*): 260 (M^+^); Anal. Calcd for C_13_H_12_N_2_O_4_: C, 60.00; H, 4.65; N, 10.76. Found: C, 59.97; H, 4.67; N, 10.58.

#### 3.8.4. Methyl 1-Cyclopropyl-5-nitro-1*H*-indole-3-carboxylate (**17d**)

Yield: 195 mg (75%) as a light yellow solid, m.p. 133–134 °C; IR: 1698, 1607, 1533, 1334 cm^−1^; ^1^H NMR (400 MHz, CDCl_3_): δ 9.03 (d, *J* = 2.3 Hz, 1H), 8.19 (dd, *J* = 9.1, 2.3 Hz, 1H), 7.98 (s, 1H), 7.64 (d, *J* = 9.1 Hz, 1H), 3.95 (s, 3H), 3.48 (septet, *J* = 3.8 Hz, 1H), 1.24 (m, 2H), 1.08 (m, 2H); ^13^C NMR (101 MHz, CDCl_3_): δ 164.3, 143.7, 140.7, 137.3, 125.9, 118.8, 118.4, 111.0, 109.4, 51.5, 28.0, 6.5; MS (*m/z*): 260 (M^+^); Anal. Calcd for C_13_H_12_N_2_O_4_: C, 60.00; H, 4.65; N, 10.76. Found: C, 60.04; H, 4.65; N, 10.61.

#### 3.8.5. Methyl 1-Cyclohexyl-5-nitro-1*H*-indole-3-carboxylate (**17e**)

Yield: 236 mg (78%) as a light yellow solid, m.p. 133–134 °C; IR: 1708, 1614, 1532, 1339 cm^−1^; ^1^H NMR (400 MHz, CDCl_3_): δ 9.07 (d, *J* = 2.3 Hz, 1H), 8.16 (dm, *J* = 9.1 Hz, 1H), 8.09 (s, 1H), 7.46 (d, *J* = 9.1 Hz, 1H), 4.28 (tt, *J* = 12.0, 3.7 Hz, 1H), 3.96 (s, 3H), 2.20 (d, *J* = 12.0 Hz, 2H), 2.00 (d, *J* = 13.5, 3.6 Hz, 2H), 1.85 (d, *J* = 13.1 Hz, 1H), 1.74 (qd, *J* = 13.1, 3.6 Hz, 2H), 1.55 (qt, *J* = 13.1, 3.6 Hz, 2H), 1.31 (qt, *J* = 13.1, 3.6 Hz, 1H); ^13^C NMR (101 MHz, CDCl_3_): δ 164.6, 143.3, 138.9, 133.8, 125.8, 118.9, 118.0, 110.1, 109.5, 56.4, 51.4, 33.4, 25.7, 25.3; MS (*m/z*): 302 (M^+^); Anal. Calcd for C_16_H_18_N_2_O_4_: C, 63.56; H, 6.00; N, 9.27. Found: C, 63.63; H, 6.05; N, 9.23.

#### 3.8.6. Methyl 1-(*tert*-Butyl)-5-nitro-1*H*-indole-3-carboxylate (**17f**)

Yield: 213 mg (77%) as a light yellow solid, m.p. 161–163 °C; IR: 1708, 1615, 1536, 1342 cm^−1^; ^1^H NMR (400 MHz, CDCl_3_): δ 9.11 (d, *J* = 2.4 Hz, 1H), 8.15 (s, 1H), 8.14 (dd, *J* = 9.3, 2.4 Hz, 1H), 7.72 (d, *J* = 9.3 Hz, 1H), 3.96 (s, 3H), 1.79 (s, 9H); ^13^C NMR (101 MHz, CDCl_3_): δ 164.6, 142.8, 138.3, 135.1, 127.6, 118.8, 117.4, 113.8, 108.4, 58.0, 51.4, 29.7; MS (*m/z*): 276 (M^+^); Anal. Calcd for C_14_H_16_N_2_O_4_: C, 60.86 H, 5.84; N, 10.14. Found: C, 60.84; H, 5.81; N, 10.06.

#### 3.8.7. Methyl 5-Nitro-1-phenethyl-1*H*-indole-3-carboxylate (**17g**)

Yield: 275 mg (85%) as a light yellow solid, m.p. 165–167 °C; IR: 1705, 1619, 1536, 1341 cm^−1^; ^1^H NMR (400 MHz, CDCl_3_): δ 9.05 (d, *J* = 2.3 Hz, 1H), 8.11 (dd, *J* = 9.1, 2.3 Hz, 1H), 7.77 (s, 1H), 7.28–7.22 (complex, 4H), 7.00 (complex, 2H), 4.43 (t, *J* = 7.0 Hz, 2H), 3.94 (s, 3H), 3.15 (t, *J* = 7.0 Hz, 2H); ^13^C NMR (101 MHz, CDCl_3_): δ 164.4, 143.4. 139.1, 137.0, 136.9, 129.0, 128.6, 127.3, 125.8, 118.9, 118.3, 110.0, 109.6, 51.5, 49.2, 36.6; MS (*m/z*): 324 (M^+^); Anal. Calcd for C_18_H_16_N_2_O_4_: C, 66.66; H, 4.97; N, 8.64. Found: C, 66.71; H, 4.96; N, 8.57.

#### 3.8.8. Methyl 1-Benzyl-5-nitro-1*H*-indole-3-carboxylate (**17h**)

Yield: 263 mg (85%) as a light yellow solid, m.p. 112–113 °C; IR: 1705, 1618, 1537, 1341 cm^−1^; ^1^H NMR (400 MHz, CDCl_3_): δ 9.09 (d, *J* = 2.2 Hz, 1H), 8.14 (dd, *J* = 9.1, 2.2 Hz, 1H), 7.98 (s, 1H), 7.39–7.32 (complex, 4H), 7.17–7.14 (complex, 2H), 5.39 (s, 2H), 3.96 (s, 3H); ^13^C NMR (101 MHz, CDCl_3_): δ 164.3, 143.5, 139.4, 137.3, 134.8, 129.3, 128.7, 127.1, 126.1, 118.9, 118.6, 110.6, 110.0, 51.5, 51.3; MS (*m/z*): 310 (M^+^); Anal. Calcd for C_17_H_14_N_2_O_4_: C, 65.80; H, 4.55; N, 9.03. Found: C, 65.74; H, 4.51; N, 8.98.

#### 3.8.9. Methyl 1-(4-Methylbenzyl)-5-nitro-1*H*-indole-3-carboxylate (**17i**)

Yield: 279 g (86%) as a light yellow solid, m.p. 163–164 °C; IR: 1706, 1616, 1538, 1349 cm^−1^; ^1^H NMR (400 MHz, CDCl_3_): δ 9.07 (d, *J* = 2.2 Hz, 1H), 8.13 (dd, *J* = 9.1, 2.2 Hz, 1H), 7.96 (s, 1H), 7.37 (d, *J* = 9.1 Hz, 1H), 7.16 (d, *J* = 7.9 Hz, 2H), 7.06 (d, *J* = 7.9 Hz, 2H), 5.33 (s, 2H), 3.95 (s, 3H), 2.33 (s, 3H); ^13^C NMR (101 MHz, CDCl_3_): δ 164.4, 143.5, 139.4, 138.6, 137.2, 131.7, 129.9, 127.2, 126.1, 118.9, 118.5, 110.6, 109.8, 51.5, 51.1, 21.1; MS (*m/z*): 324 (M^+^); Anal. Calcd for C_18_H_16_N_2_O_4_: C, 66.66; H, 4.97; N, 8.64. Found: C, 66.61; H, 4.95; N, 8.62.

#### 3.8.10. Methyl 1-(3-Methoxybenzyl)-5-nitro-1*H*-indole-3-carboxylate (**17j**)

Yield: 282 mg (83%) as a light yellow solid, m.p. 112–113 °C; IR: 2840, 1704, 1611, 1547, 1338 cm^−1^; ^1^H NMR (400 MHz, CDCl_3_): δ 9.09 (d, *J* = 2.2 Hz, 1H), 8.14 (dd, *J* = 9.1, 2.2 Hz, 1H), 7.98 (s, 1H), 7.37 (d, *J* = 9.1 Hz, 1H), 7.27 (t, *J* = 7.8 Hz, 1H), 6.87 (dd, *J* = 8.6, 2.2 Hz, 1H), 6.73 (d, *J* = 7.8 Hz, 1H), 6.67 (t, *J* = 2.2 Hz, 1H), 5.35 (s, 2H), 3.96 (s, 3H), 3.76 (s, 3H); ^13^C NMR (101 MHz, CDCl_3_): δ 164.3, 160.2, 143.5, 139.4, 137.3, 136.3, 130.4, 126.1, 119.3, 118.9, 118.6, 113.5, 113.2, 110.6, 110.0, 55.3, 51.5, 51.2; MS (*m/z*): 340 (M^+^); Anal. Calcd for C_18_H_16_N_2_O_5_: C, 63.53; H, 4.74; N, 8.23. Found: C, 63.44; H, 4.77; N, 8.17.

#### 3.8.11. Methyl 1-(4-Methoxybenzyl)-5-nitro-1*H*-indole-3-carboxylate (**17k**)

Yield: 296 mg (87%) as a light yellow solid, m.p. 153–154 °C; IR: 2838, 1705, 1614, 1537, 1342 cm^−1^; ^1^H NMR (400 MHz, CDCl_3_): δ 9.08 (d, *J* = 2.3 Hz, 1H), 8.14 (dd, *J* = 9.1, 2.3 Hz, 1H), 7.95 (s, 1H), 7.39 (d, *J* = 9.1 Hz, 1H), 7.12 (d, *J* = 8.7 Hz, 2H), 6.88 (d, *J* = 8.7 Hz, 2H), 5.31 (s, 2H), 3.95 (s, 3H), 3.80 (s, 3H); ^13^C NMR (101 MHz, CDCl_3_): δ 164.4, 159.9, 143.5, 139.3, 137.1, 128.8, 126.6, 126.1, 118.9, 118.5, 114.6, 110.6, 109.8, 55.4, 51.5, 50.8; MS (*m/z*): 340 (M^+^); Anal. Calcd for C_18_H_16_N_2_O_5_: C, 63.53; H, 4.74; N, 8.23. Found: C, 63.49; H, 4.74; N, 8.21.

#### 3.8.12. Methyl 1-(4-Chlorobenzyl)-5-nitro-1*H*-indole-3-carboxylate (**17l**)

Yield: 279 mg (81%) as a light yellow solid, m.p. 167–169 °C; IR: 1706, 1611, 1537, 1342 cm^−1^; ^1^H NMR (400 MHz, CDCl_3_): δ 9.07 (d, *J* = 2.2 Hz, 1H), 8.13 (dd, *J* = 9.1, 2.2 Hz, 1H), 7.96 (s, 1H), 7.33 (d, *J* = 8.5 Hz, 2H), 7.32 (d, *J* = 9.1 Hz, 1H), 7.09 (d, *J* = 8.5 Hz, 2H), 5.37 (s, 2H), 3.96 (s, 3H); ^13^C NMR (101 MHz, CDCl_3_): δ 164.2, 149.6, 139.2, 137.1, 134.7, 133.3, 129.5, 128.4, 126.1, 119.0, 118.7, 110.5, 110.2, 51.6, 50.6; MS (*m/z*): 344 (M^+^); Anal. Calcd for C_17_H_13_ClN_2_O_4_: C, 59.23; H, 3.80; N, 8.13. Found: C, 59.16; H, 3.77; N, 8.07.

#### 3.8.13. Methyl 5-Nitro-1-((4-trifluoromethyl)benzyl)-1*H*-indole-3-carboxylate (**17m**)

Yield: 336 mg (89%) as a light yellow solid, m.p. 162–164 °C; IR: 1707, 1616, 1538, 1343, 1325 cm^−1^; ^1^H NMR (400 MHz, CDCl_3_): δ 9.11 (d, *J* = 2.3 Hz, 1H), 8.15 (dd, *J* = 9.1, 2.3 Hz, 1H), 7.99 (s, 1H), 7.62 (d, *J* = 8.1 Hz, 2H), 7.43 (s, 1H), 7.32 (d, *J* = 9.1 Hz, 1H), 7.24 (dt, *J* = 8.1 Hz, 1H), 5.47 (s, 2H), 3.97 (s, 3H); ^13^C NMR (101 MHz, CDCl_3_): δ 164.1, 143.7, 139.2, 138.9, 137.1, 131.0 (q, *J* = 32.8 Hz), 127.2, 126.3 (q, *J* = 3.7 Hz), 126.1, 123.7 (q, *J* = 272.4 Hz), 119.1, 118.8, 110.5, 110.4, 51.6, 50.7; MS (*m/z*): 378 (M^+^); Anal. Calcd for C_18_H_13_F_3_N_2_O_4_: C, 57.15; H, 3.46; N, 7.41. Found: C, 57.24; H, 3.49; N, 7.30.

#### 3.8.14. Methyl 5-Nitro-1-(3-nitrobenzyl)-1*H*-indole-3-carboxylate (**17n**)

Yield: 323 mg (91%) as a yellow solid, m.p. 147–149 °C; IR: 1706, 1618, 534, 1344 cm^−1^; ^1^H NMR (400 MHz, CDCl_3_): δ 9.11 (d, *J* = 2.3 Hz, 1H), 8.24 (d, *J* = 8.1 Hz, 1H), 8.16 (dd, *J* = 9.1, 2.3 Hz, 1H), 8.08 (br s, 1H), 8.02 (s, 1H), 7.56 (t, *J* = 8.0 Hz, 1H), 7.41 (d, *J* = 8.0 Hz, 1H), 7.33 (t, *J* = 9.1 Hz, 1H), 5.52 (s, 2H), 3.98 (s, 3H); ^13^C NMR (101 MHz, CDCl_3_): δ 164.1, 148.8, 143.8, 139.1, 137.1, 136.9, 132.6, 130.5, 126.2, 123.7, 121.9, 119.2, 119.0, 110.8, 110.2, 51.7, 50.4; MS (*m/z*): 355 (M^+^); Anal. Calcd for C_17_H_13_N_3_O_6_: C, 57.47; H, 3.69; N, 11.83. Found: C, 57.39; H, 3.65; N, 11.72.

#### 3.8.15. Methyl 1-Amino-5-nitro-1*H*-indole-3-carboxylate (**17o**)

Yield: 195 mg (83%) as a tan solid, m.p. 199–200 °C; IR: 3333, 3125, 1698 cm^−1^; ^1^H NMR (400 MHz, DMSO-*d_6_*): δ 8.86 (d, *J* = 2.3 Hz, 1H), 8.19 (s, 1H), 8.18 (dd, *J* = 9.1, 2.3 Hz, 1H), 7.75 (d, *J* = 9.1 Hz, 1H), 6.49 (s, 2H), 3.86 (s, 3H); ^13^C NMR (101 MHz, DMSO-*d_6_*): δ 163.0, 142.0, 139.2, 138.5, 122.5, 117.1, 116.4, 111.1, 104.3, 50.6; MS (*m/z*): 235 (M^+^); Anal. Calcd for C_10_H_9_N_3_O_4_: C, 51.07; H, 3.86; N, 17.87. Found: C, 51.08; H, 3.83; N, 17.77.

### 3.9. Reactions with Methyl 2-(5-Cyano-2-fluorophenyl)acrylate (***10***)

#### 3.9.1. Methyl 5-cyano-1-hexyl-1*H*-indole-3-carboxylate (**18a**)

Yield: 210 mg (74%) as a white solid, m.p. 71–73 °C; IR: 2223, 1705, 1612 cm^−1^; ^1^H NMR (400 MHz, CDCl_3_): δ 8.53 (br s, 1H), 7.92 (s, 1H), 7.51 (dd, *J* = 8.6, 1.6 Hz, 1H), 7.42 (dd, *J* = 8.6, 0.8 Hz, 1H), 4.15 (t, *J* = 7.1 Hz, 2H), 3.94 (s, 3H), 1.88 (quintet, *J* = 7.1 Hz, 2H), 1.31 (m, 6H), 0.87 (t, *J* = 6.8 Hz, 3H); ^13^C NMR (101 MHz, CDCl_3_): δ 164.6, 138.0, 136.1, 127.4, 126.3, 125.6, 120.2, 111.0, 108.0, 105.1, 51.4, 47.3, 31.2, 29.7, 26.4, 22.4, 14.1; MS (*m/z*): 284 (M^+^); Anal. Calcd for C_17_H_20_N_2_O_2_: C, 71.81; H, 7.09; N, 9.85. Found: C, 71.87; H, 7.13; N, 9.73. 

#### 3.9.2. Methyl 5-Cyano-1-isobutyl-1*H*-indole-3-carboxylate (**18b**)

Yield: 172 mg (67%) as a white solid, m.p. 70–72 °C; IR: 2223, 1705, 1612 cm^−1^; ^1^H NMR (400 MHz, CDCl_3_): δ 8.53 (br s, 1H), 7.90 (s, 1H), 7.50 (dd, *J* = 8.6, 1.6 Hz, 1H), 7.42 (dd, *J* = 8.6, 0.7 Hz, 1H), 3.98 (d, *J* = 7.4 Hz, 2H), 3.94 (s, 3H), 2.21 (nonet, *J* = 6.9 Hz, 1H), 0.95 (d, *J* = 6.7 Hz, 6H); ^13^C NMR (101 MHz, CDCl_3_): δ 164.6, 138.3, 136.6, 127.4, 126.3, 125.6, 120.2, 111.2, 108.0, 105.1, 54.8, 51.4, 29.4, 20.1; MS (*m/z*) 256 (M^+^); Anal. Calcd for C_15_H_16_N_2_O_2_: C, 70.29; H, 6.29; N, 10.93. Found: C, 70.26; H, 6.27; N, 10.87. 

#### 3.9.3. Methyl 5-Cyano-1-cyclopropyl-1*H*-indole-3-carboxylate (**18d**)

Yield: 187 mg (78%) as a white solid, m.p. 132–134 °C; IR: 2222, 1706, 1614 cm^−1^; ^1^H NMR (400 MHz, CDCl_3_): δ 8.48 (br s, 1H), 7.94 (s, 1H), 7.65 (dd, *J* = 8.5, 0.8 Hz, 1H), 7.51 (dd, *J* = 8.5, 1.6 Hz, 1H), 3.92 (s, 3H), 3.45 (apparent septet, *J* = 3.6 Hz, 1H), 1.21 (m, 2H), 1.06 (m, 2H); ^13^C NMR (101 MHz, CDCl_3_): δ 164.4, 139.5, 136.4, 127.3, 126.4, 125.8, 120.2, 111.8, 108.0, 105.5, 51.4, 27.8, 6.4; MS (*m/z*): 240 (M^+^); Anal. Calcd for C_14_H_12_N_2_O_2_: C, 69.99; H, 5.03; N, 11.66. Found: C, 69.94; H, 4.97; N, 11.59. 

#### 3.9.4. Methyl 5-Cyano-1-cyclohexyl-1*H*-indole-3-carboxylate (**18e**)

Yield: 223 mg (79%) as a white solid, m.p. 132–134 °C; IR: 2223, 1705, 1614 cm^−1^; ^1^H NMR (400 MHz, CDCl_3_): δ 8.53 (br s, 1H), 8.05 (s, 1H), 7.50 (dd, *J* = 8.6, 1.6 Hz, 1H), 7.47 (dd, *J* = 8.6, 0.8 Hz, 1H), 4.25 (tt, *J* = 11.9, 3.7 Hz, 1H), 3.94 (s, 3H), 2.17 (d, *J* = 12.8 Hz, 2H), 2.00 (dt, *J* = 13.5, 3.5 Hz, 2H), 1.84 (dd, *J* = 13.2 Hz, 1H), 1.76 (qd, *J* = 13.2, 3.5 Hz, 2H), 1.53 (qt, *J* = 13.2, 3.5 Hz, 2H), 1.33 (qt, *J* = 13.0, 3.5 Hz, 1H); ^13^C NMR (101 MHz, CDCl_3_): δ 164.7, 137.7, 132.9, 127.4, 126.3, 125.4, 120.3, 111.0, 108.1, 105.1, 56.1, 51.3, 33.4, 25.7, 25.3; MS (*m/z*): 282 (M^+^); Anal. Calcd for C_17_H_18_N_2_O_2_: C, 72.32; H, 6.43; N, 9.92. Found: C, 72.34; H, 6.44; N, 9.85. 

#### 3.9.5. Methyl 5-Cyano-1-phenethyl-1*H*-indole-3-carboxylate (**18g**)

Yield: 277 mg (91 %) as a white solid, m.p. 112–114 °C; IR: 2222, 1702, 1615 cm^−1^; ^1^H NMR (400 MHz, CDCl_3_): δ 8.51 (s, 1H), 7.73 (s, 1H), 7.45 (d, *J* = 8.5 Hz, 1H), 7.30 (d, *J* = 8.5 Hz, 1H), 7.27–7.20 (complex, 3H), 7.04–6.96 (complex, 2H), 4.42 (t, *J* = 7.0 Hz, 2H), 3.91 (s, 3H), 3.13 (t, *J* = 7.0 Hz, 2H); ^13^C NMR (101 MHz, CDCl_3_): δ 164.5, 137.9, 137.1, 136.1, 128.9, 128.6, 127.4, 127.3, 126.2, 125.7, 120.2, 110.9, 108.1, 105.1, 51.4, 48.9, 36.5; MS (*m/z*): 304 (M^+^); Anal. Calcd for C_19_H_16_N_2_O_2_: C, 74.98; H, 5.30; N, 9.20. Found: C, 74.95; H, 5.28; N, 9.14. 

#### 3.9.6. Methyl 1-Benzyl-5-cyano-1*H*-indole-3-carboxylate (**18h**)

Yield: 255 mg (88%) as a white solid, m.p. 131–133 °C; IR: 2223, 1705, 1614 cm^−1^; ^1^H NMR (400 MHz, CDCl_3_): δ 8.55 (d, *J* = 1.5 Hz, 1H), 7.94 (s, 1H), 7.47 (dd, *J* = 8.5, 1.5 Hz, 1H), 7.40–7.32 (complex, 4H), 7.15–7.13 (complex, 2H), 5.36 (s, 2H), 3.93 (s, 3H); ^13^C NMR (101 MHz, CDCl_3_): δ 164.5, 138.2, 136.4, 134.9, 129.2, 128.6, 127.5, 127.1, 126.5, 125.9, 120.1, 111.3, 108.6, 105.4, 51.4, 51.1; MS (*m/z*): 290 (M^+^); Anal. Calcd for C_18_H_14_N_2_O_2_: C, 74.47; H, 4.86; N, 9.65. Found: C, 74.44; H, 4.86; N, 9.63. 

#### 3.9.7. Methyl 5-Cyano-1-(4-methylbenzyl)-1*H*-indole-3-carboxylate (**18i**)

Yield: 246 mg (81%) as a white solid, m.p. 139–141 °C; IR: 2222, 1704, 1615 cm^−1^; ^1^H NMR (400 MHz, CDCl_3_): δ 8.54 (br s, 1H), 7.92 (s, 1H), 7.46 (dd, *J* = 8.5, 1.6 Hz, 1H), 7.38 (dd, *J* = 8.5, 0.8 Hz, 1H), 7.15 (d, *J* = 7.8 Hz, 2H), 7.05 (d, *J* = 7.8 Hz, 2H), 5.31 (s, 2H), 3.93 (s, 3H), 2.33 (s, 3H); ^13^C NMR (101 MHz, CDCl_3_): δ 164.5, 138.5, 138.2, 136.4, 131.8, 129.9, 127.4, 127.2, 126.5, 125.9, 120.1, 111.4, 108.4, 105.4, 51.4, 50.9, 21.1; MS (*m/z*): 304 (M^+^); Anal. Calcd for C_19_H_16_N_2_O_2_: C, 74.98; H, 5.30; N, 9.20. Found: C, 74.94; H, 5.29; N, 9.16. 

#### 3.9.8. Methyl 5-Cyano-1-(3-methoxybenzyl)-1*H*-indole-3-carboxylate (**18j**)

Yield: 265 mg (83%) as a white solid, m.p. 118–120 °C; IR: 2837, 2222, 1702, 1612 cm^−1^; ^1^H NMR (400 MHz, CDCl_3_): δ 8.55 (br s, 1H), 7.94 (s, 1H), 7.47 (dd, *J* = 8.6, 1.6 Hz, 1H), 7.37 (dd, *J* = 8.6, 0.7 Hz, 1H), 7.27 (t, *J* = 8.1 Hz, 1H), 6.86 (dd, *J* = 8.1, 2.5 Hz, 1H), 6.71 (d, *J* = 7.6 Hz, 1H), 6.65 (br t, *J* = 2.1 Hz, 1H), 5.32 (s, 2H), 3.94 (s, 3H), 3.75 (s, 3H); ^13^C NMR (101 MHz, CDCl_3_): δ 164.5, 160.2, 138.2, 136.4, 130.4, 127.5, 126.5, 126.0, 120.1, 119.2, 113.5, 113.2, 111.3, 108.6, 105.5, 55.3, 51.4, 51.0 (one aromatic carbon unresolved); MS (*m/z*): 320 (M^+^); Anal. Calcd for C_19_H_16_N_2_O_3_: C, 71.24; H, 5.03; N, 8.74. Found: C, 71.17; H, 4.99; N, 8.68.

#### 3.9.9. Methyl 5-Cyano-1-(4-methoxybenzyl)-1*H*-indole-3-carboxylate (**18k**)

Yield: 285 mg (89%) as a white solid, m.p. 130–132 °C; IR: 2840, 2222, 1703, 1613 cm^−1^; ^1^H NMR (400 MHz, CDCl_3_): δ 8.54 (br s, 1H), 7.90 (s, 1H), 7.47 (dd, *J* = 8.6, 1.6 Hz, 1H), 7.40 (dd, *J* = 8.6, 0.7 Hz, 1H), 7.11 (d, *J* = 8.7 Hz, 2H), 6.88 (d, *J* = 8.7 Hz, 2H), 5.28 (s, 2H), 3.93 (s, 3H), 3.79 (s, 3H); ^13^C NMR (101 MHz, CDCl_3_): δ 164.5, 159.8, 138.1, 136.2, 128.7, 127.4, 126.7, 126.5, 125.8, 120.1, 114.6, 111.3, 108.4, 105.4, 55.3, 51.4, 50.6; MS (*m/z*): 320; (M^+^) Anal. Calcd for C_19_H_16_N_2_O_3_: C, 71.24; H, 5.03; N, 8.74. Found: C, 71.23; H, 5.01; N, 8.66. 

#### 3.9.10. Methyl 1-(4-Chlorobenzyl)-5-cyano-1*H*-indole-3-carboxylate (**18l**)

Yield: 282 mg (87%) as a white solid, m.p. 193–195 °C; IR: 2223, 1704, 1614 cm^−1^; ^1^H NMR (400 MHz, CDCl_3_): δ 8.54 (br s, 1H), 7.93 (s, 1H), 7.46 (dd, *J* = 8.6, 1.6 Hz, 1H), 7.34 (dd, *J* = 8.6, 0.7 Hz, 1H), 7.31 (d, *J* = 8.5 Hz, 2H), 7.08 (d, *J* = 8.5 Hz, 2H), 5.34 (s, 2H), 3.93 (s, 3H); ^13^C NMR (101 MHz, CDCl_3_): δ 164.3, 138.0, 136.2, 134.6, 133.4, 129.5, 128.4, 127.5, 126.5, 126.0, 120.0, 111.2, 108.8, 105.6, 51.5, 50.4; MS (*m/z*): 324 (M^+^); Anal. Calcd for C_18_H_13_ClN_2_O_2_: C, 66.57; H, 4.03; N, 8.63. Found: C, 66.57; H, 4.02; N, 8.59.

#### 3.9.11. Methyl 5-Cyano-1-(3-nitrobenzyl)-1*H*-indole-3-carboxylate (**18n**)

Yield: 308 mg (92%) as a light yellow solid, m.p. 165–167 °C; IR: 2223, 1702, 1614, 1533, 1350 cm^−1^; ^1^H NMR (400 MHz, CDCl_3_): δ 8.57 (br s, 1H), 8.21 (dd, *J* = 8.2, 1.3 Hz, 1H), 8.07 (br t, *J* = 2.1 Hz, 1H), 7.98 (s, 1H), 7.55 (t, *J* = 8.0 Hz, 1H), 7.49 (dd, *J* = 8.6, 1.6 Hz, 1H), 7.39 (d, *J* = 7.7 Hz, 1H), 7.33 (d, *J* = 8.6 Hz, 1H), 5.49 (s, 2H), 3.95 (s, 3H); ^13^C NMR (101 MHz, CDCl_3_): δ 164.2, 148.8, 137.9, 137.2, 136.1, 132.6, 130.5, 127.8, 126.6, 126.4, 123.7, 121.9, 119.8, 111.0, 109.5, 106.0, 51.6, 50.2; MS (*m/z*): 335 (M^+^); Anal. Calcd for C_18_H_13_N_3_O_4_: C, 64.48; H, 3.91; N, 12.53. Found: C, 64.42; H, 3.88; N, 12.47. 

### 3.10. Methyl 2-(5-Carbomethoxy-2-fluorophenyl)acrylate (***13***)

#### 3.10.1. Dimethyl 1-Hexyl-1*H*-indole-3,5-dicarboxylate (**19a**)

Yield: 212 mg (67%) as a white solid, m.p. 64–66 °C; IR: 1719, 1634 cm^−1^; ^1^H NMR (400 MHz, CDCl_3_): δ 8.88 (d, *J* = 1.7 Hz, 1H), 7.98 (dd, *J* = 8.7, 1.7 Hz, 1H), 7.87 (s, 1H), 7.37 (d, *J* = 8.7 Hz, 1H), 4.14 (t, *J* = 7.2 Hz, 2H), 3.95 (s, 3H), 3.94 (s, 3H), 1.86 (quintet, *J* = 7.2 Hz, 2H), 1.31 (m, 6H), 0.86 (t, *J* = 6.8 Hz, 3H); ^13^C NMR (101 MHz, CDCl_3_): δ 167.9, 165.1, 139.0, 135.6, 126.1, 124.5, 124.1, 123.8, 109.8, 108.3, 52.0, 51.2, 47.2, 31.3, 29.9, 26.5, 22.5, 14.0; MS (*m/z*): 317 (M^+^); Anal. Calcd for C_18_H_23_NO_4_: C, 68.12; H, 7.30; N, 4.41. Found: C, 68.19; H, 7.31; N, 4.33. 

#### 3.10.2. Dimethyl 1-Isobutyl-1*H*-indole-3,5-dicarboxylate (**19b**)

Yield: 199 mg (69%) as a white solid, m.p. 125–126 °C; IR: 1717, 1631 cm^−1^; ^1^H NMR (400 MHz, CDCl_3_): δ 8.88 (dd, *J* = 1.7, 0.7 Hz, 1H), 7.98 (dd, *J* = 8.7, 1.7 Hz, 1H), 7.86 (s, 1H), 7.37 (dd, *J* = 8.7, 0.7 Hz, 1H), 3.96 (d, *J* = 7.2 Hz, 2H), 3.952 (s, 3H), 3.948 (s, 3H), 2.22 (septet, *J* = 6.8 Hz, 1H), 0.95 (d, *J* = 6.7 Hz, 6H); ^13^C NMR (101 MHz, CDCl_3_): δ 167.9, 165.1, 139.2, 136.1, 126.0, 124.5, 124.1, 123.9, 110.0, 108.3, 54.8, 52.0, 51.2, 29.3, 20.2; MS (*m/z*): 289 (M^+^); Anal. Calcd for C_16_H_19_NO_4_: C, 66.42; H, 6.62; N, 4.84. Found: C, 66.47; H, 6.65; N, 4.78.

#### 3.10.3. Dimethyl 1-Allyl-1*H*-indole-3,5-dicarboxylate (**19c**)

Yield: 218 mg (80 %) as a white solid, m.p. 102–103 °C; IR: 1710, 1645, 1617 cm^−1^; ^1^H NMR (400 MHz, CDCl_3_): δ 8.88 (d, *J* = 1.7 Hz, 1H), 7.98 (dd, *J* = 8.7, 1.7 Hz, 1H), 7.88 (s, 1H), 7.36 (d, *J* = 8.7 Hz, 1H), 6.00 (ddt, *J* = 15.8, 10.3, 5.4 Hz, 1H), 5.60 (d, *J* = 10.3 Hz, 1H), 5.35 (d, *J* = 15.8 Hz, 1H), 4.78 (d, *J* = 5.4 Hz, 2H), 3.95 (s, 3H), 3.94 (s, 3H); ^13^C NMR (101 MHz, CDCl_3_): δ 167.9, 165.0, 139.0, 135.6, 131.7, 126.1, 124.5, 124.2, 124.0, 118.9, 110.0, 108.7, 52.0, 51.3, 49.5; MS (*m/z*): 273 (M^+^); Anal. Calcd for C_15_H_15_NO_4_: C, 65.92; H, 5.53; N, 5.13. Found: C, 65.87; H, 5.48; N, 5.09.

#### 3.10.4. Dimethyl 1-Cyclopropyl-1*H*-indole-3,5-dicarboxylate (**19d**)

Yield: 166 mg (61%) as a white solid, m.p. 136–137 °C; IR: 1709, 1621 cm^−1^; ^1^H NMR (400 MHz, CDCl_3_): δ 8.84 (d, *J* = 1.7 Hz, 1H), 8.00 (dd, *J* = 8.7, 1.7 Hz, 1H), 7.90 (s, 1H), 7.60 (d, *J* = 8.7 Hz, 1H), 3.95 (s, 3H), 3.93 (s, 3H), 3.44 (m, 1H), 1.17 (m, 2H), 1.05 (m, 2H); ^13^C NMR (101 MHz, CDCl_3_): δ 167.9, 165.0, 140.4, 135.9, 126.1, 124.4, 124.3, 124.2, 110.6, 108.4, 52.0, 51.2, 27.8, 6.3; MS (*m/z*): 273 (M^+^); Anal. Calcd for C_15_H_15_NO_4_: C, 65.92; H, 5.53; N, 5.13. Found: C, 65.81; H, 5.55; N, 5.06. 

#### 3.10.5. Dimethyl 1-Cyclohexyl-1*H*-indole-3,5-dicarboxylate (**19e**)

Yield: 205 mg (65%) as a white solid, m.p. 138–140 °C; IR: 1703, 1618 cm^−1^; ^1^H NMR (400 MHz, CDCl_3_): δ 8.88 (d, *J* = 1.7 Hz, 1H), 8.01 (s, 1H), 7.98 (dd, *J* = 8.7, 1.7 Hz, 1H), 7.42 (d, *J* = 8.7 Hz, 1H), 4.26 (tt, *J* = 11.9, 3.7 Hz, 1H), 3.95 (s, 3H), 3.94 (s, 3H), 2.18 (d, *J* = 12.8 Hz, 2H), 1.98 (dd, *J* = 13.5, 3.5 Hz, 2H), 1.83 (d, *J* = 13.2 Hz, 1H), 1.74 (qd, *J* = 13.2, 3.5 Hz, 2H), 1.53 (qt, *J* = 13.2, 3.5 Hz, 2H), 1.33 (tt, *J* = 13.0, 3.5 Hz, 1H); ^13^C NMR (101 MHz, CDCl_3_): δ 167.9, 165.2, 138.6, 132.4, 126.0, 124.5, 123.88, 123.86, 109.8, 108.4, 54.9, 52.0, 51.2, 33.4, 25.7, 25.4; MS (*m/z*): 315 (M^+^); Anal. Calcd for C_18_H_21_NO_4_: C, 68.55; H, 6.71; N, 4.44. Found: C, 68.61; H, 6.73; N, 4.42.

#### 3.10.6. Dimethyl 1-Phenethyl-1*H*-indole-3,5-dicarboxylate (**19g**)

Yield: 300 mg (89 %) as a white solid, m.p. 152–154 °C; IR: 1706 cm^−1^; ^1^H NMR (400 MHz, CDCl_3_): δ 8.87 (d, *J* = 1.7 Hz, 1H), 7.96 (dd, *J* = 8.7, 1.7 Hz, 1H), 7.70 (s, 1H), 7.30 (d, *J* = 8.7 Hz, 1H), 7.28–7.21 (complex, 3H), 7.04–7.01 (complex, 2H), 4.39 (t, *J* = 7.2 Hz, 2H), 3.95 (s, 3H), 3.92 (s, 3H), 3.14 (t, *J* = 7.2 Hz, 2H); ^13^C NMR (101 MHz, CDCl_3_): δ 167.9, 165.0, 138.8, 137.3, 135.6, 128.8, 128.6, 127.1, 126.1, 124.5, 124.2, 123.9, 109.7, 108.4, 52.0, 51.2, 48.8, 36.5; MS (*m/z*): 337 (M^+^); Anal. Calcd for C_20_H_19_NO_4_: C, 71.20; H, 5.68; N, 4.15. Found: C, 71.18; H, 5.68; N, 4.10.

#### 3.10.7. Dimethyl 1-Benzyl-1*H*-indole-3,5-dicarboxylate (**19h**)

Yield: 278 mg (86%) as a white solid, m.p. 140–142 °C; IR: 1709, 1611 cm^−1^; ^1^H NMR (400 MHz, CDCl_3_): δ 8.90 (d, *J* = 1.7 Hz, 1H), 7.94 (dd, *J* = 8.7, 1.7 Hz, 1H), 7.89 (s, 1H), 7.37–7.30 (complex, 4H), 7.17–7.13 (complex, 2H), 5.35 (s, 2H), 3.942 (s, 3H), 3.937 (s, 3H); ^13^C NMR (101 MHz, CDCl_3_): δ 167.8, 165.0, 139.2, 135.9, 135.4, 129.1, 128.4, 127.1, 126.2, 124.5, 124.4, 124.1, 110.1, 109.0, 52.0, 51.3, 50.9; MS (*m/z*): 323 (M^+^); Anal. Calcd for C_19_H_17_NO_4_: C, 70.58; H, 5.30; N, 4.33. Found: C, 70.56; H, 5.27; N, 4.25.

#### 3.10.8. Dimethyl 1-(4-Methylbenzyl)-1*H*-indole-3,5-dicarboxylate (**19i**)

Yield: 276 mg (82%) as a white solid, m.p. 146–147 °C; IR: 1710, 1609 cm^−1^; ^1^H NMR (400 MHz, CDCl_3_): δ 8.89 (d, *J* = 1.7 Hz, 1H), 7.95 (dd, *J* = 8.7, 1.7 Hz, 1H), 7.87 (s, 1H), 7.34 (d, *J* = 8.7 Hz, 1H), 7.14 (d, *J* = 7.9 Hz, 2H), 7.05 (d, *J* = 7.9 Hz, 2H), 5.29 (s, 2H), 3.94 (s, 3H), 3.93 (s, 3H), 2.32 (s, 3H); ^13^C NMR (101 MHz, CDCl_3_): δ 167.8, 165.0, 139.1, 138.3, 135.8, 132.3, 129.8, 127.2, 126.2, 124.5, 124.3, 124.1, 110.1, 108.8, 52.0, 51.2, 50.7, 21.1; MS (*m/z*): 337 (M^+^); Anal. Calcd for C_20_H_19_NO_4_: C, 71.20; H, 5.68; N, 4.15. Found: C, 71.14; H, 5.64; N, 4.11.

#### 3.10.9. Dimethyl 1-(3-Methoxybenzyl)-1*H*-indole-3,5-dicarboxylate (**19j**)

Yield: 303 mg (86%) as a white solid, m.p. 131–132 °C; IR: 2831, 1705, 1622 cm^−1^; ^1^H NMR (400 MHz, CDCl_3_): δ 8.89 (br s, 1H), 7.96 (dd, *J* = 8.7, 1.7 Hz, 1H), 7.89 (s, 1H), 7.34 (d, *J* = 8.7 Hz, 1H), 7.25 (t, *J* = 8.1 Hz, 1H), 6.84 (t, *J* = 2.5 Hz, 1H), 6.74 (d, *J* = 7.5 Hz, 1H), 6.66 (t, *J* = 2.5 Hz, 1H), 5.32 (s, 2H), 3.945 (s, 3H), 3.941 (s, 3H), 3.74 (s, 3H); ^13^C NMR (101 MHz, CDCl_3_): δ 167.8, 165.0, 160.1, 139.2, 136.9, 135.9, 130.2, 126.2, 124.5, 124.4, 124.2, 119.3, 113.4, 113.0, 110.1, 109.0, 55.3, 52.0, 51.3, 50.9; MS (*m/z*): 353 (M^+^); Anal. Calcd for C_20_H_19_NO_5_: C, 67.98; H, 5.42; N, 3.96. Found: C, 67.91; H, 5.40, N, 3.88.

#### 3.10.10. Dimethyl 1-(4-Methoxybenzyl)-1*H*-indole-3,5-dicarboxylate (**19k**)

Yield: 304 mg (86%) as a white solid, m.p. 135–136 °C; IR: 2833, 1705, 1620 cm^−1^; ^1^H NMR (400 MHz, CDCl_3_): δ 8.89 (d, *J* = 1.7 Hz, 1H), 7.96 (dd, *J* = 8.7, 1.7 Hz, 1H), 7.86 (s, 1H), 7.36 (d, *J* = 8.7 Hz, 1H), 7.12 (d, *J* = 8.6 Hz, 2H), 6.87 (d, *J* = 8.6 Hz, 2H), 5.28 (s, 2H), 3.94 (s, 3H), 3.93 (s, 3H), 3.79 (s, 3H); ^13^C NMR (101 MHz, CDCl_3_): δ 167.8, 165.0, 159.7, 139.1, 135.7, 128.7, 127.2, 126.3, 124.5, 124.3, 124.1, 114.5, 110.1, 108.8, 55.3, 52.0, 51.3, 50.5; MS (*m/z*): 353 (M^+^); Anal. Calcd for C_20_H_19_NO_5_: C, 67.98; H, 5.42; N, 3.96. Found: C, 68.02; H, 5.41; N, 3.93.

#### 3.10.11. Dimethyl 1-(4-Chlorobenzyl)-1*H*-indole-3,5-dicarboxylate (**19l**)

Yield: 286 mg (80%) as a white solid, m.p. 140–142 °C; IR: 1709, 1617 cm^−1^; ^1^H NMR (400 MHz, CDCl_3_): δ 8.90 (d, *J* = 1.7 Hz, 1H), 7.96 (dd, *J* = 8.7, 1.7 Hz, 1H), 7.88 (s, 1H), 7.31 (d, *J* = 8.5 Hz, 2H), 7.29 (d, *J* = 8.7 Hz, 1H), 7.08 (d, *J* = 8.5 Hz, 2H), 5.32 (s, 2H), 3.946 (s, 3H), 3.943 (s, 3H); ^13^C NMR (101 MHz, CDCl_3_): δ 167.7, 164.9, 139.0, 135.6, 134.4, 133.9, 129.3, 128.4, 126.2, 124.6, 124.5, 124.3, 110.0, 109.2, 52.0, 51.3, 50.3; MS (*m/z*): 357 (M^+^); Anal. Calcd for C_19_H_16_ClNO_4_: C, 63.78; H, 4.51; N, 3.91. Found: C, 63.72; H, 4.47; N, 3.83. 

#### 3.10.12. Dimethyl 1-(3-Nitrobenzyl)-1*H*-indole-3,5-dicarboxylate (**19n**)

Yield: 334 mg (91%) as a white solid, m.p. 234–236 °C; IR: 1695, 1615, 1514, 1330 cm^−1^; ^1^H NMR (400 MHz, CDCl_3_): δ 8.92 (d, *J* = 1.7 Hz, 1H), 8.19 (d, *J* = 8.1 Hz, 1H), 8.08 (s, 1H), 7.97 (dd, *J* = 8.7, 17 Hz, 1H), 7.93 (s, 1H), 7.52 (t, *J* = 8.0 Hz, 1H), 7.38 (d, *J* = 8.1 Hz, 1H), 7.28 (d, *J* = 8.7 Hz, 1H), 5.48 (s, 2H), 3.96 (s, 3H), 3.95 (s, 3H); ^13^C NMR (101 MHz, CDCl_3_): δ 167.6, 164.7, 148.7, 138.9, 137.7, 135.5, 132.6, 130.4, 126.3, 124.84, 124.80, 124.6, 123.5, 121.9, 109.9, 109.8, 52.1, 51.4, 50.1; MS (*m/z*): 368 (M^+^); Anal. Calcd for C_19_H_16_N_2_O_6_: C, 61.96; H, 4.38; N, 7.61. Found: C, 61.93; H, 4.39; N, 7.57.

## 4. Conclusions

A method has been developed for the efficient synthesis of 1-alkyl-1*H-*indole-3-carboxylic esters that uses a domino aza-Michael-S_N_Ar-heteroaromatization sequence. Following synthesis of the substrates, the reaction was performed using an equimolar mixture of the acrylate and the amine in the presence of 2 equiv of K_2_CO_3_ in anhydrous DMF. The reaction proceeded at room temperature for substrates with nitro and cyano activated S_N_Ar acceptor rings and at 50–90 °C for rings activated by an ester. The amines were all primary alkylamines with no restriction on the structure of the alkyl group. Anilines did not undergo the ring formation due to their reduced nucleophilicity. The entire process occurred in a single reaction flask to give the aromatized product. The anticipated indoline products were not produced, but instead, oxidation to the aromatic indoles was observed. The heteroaromatization is believed to be promoted by oxygen dissolved in the DMF solvent or introduced during removal of samples for TLC analysis. In no case was an indoline observed or isolated from the reaction. Hydrazine reacted with the nitro activated substrate, but failed for substrates with less active S_N_Ar acceptor rings, giving products resulting from reaction with the cyano and ester substituents. The corresponding 2-arylacrylonitrile substrate polymerized under the aldol conditions with formalin while the phenylsulfonyl precursor to the vinyl sulfone failed to undergo aldol condensation with formaldehyde using K_2_CO_3_ as the base.

## Figures and Tables

**Table 1:**
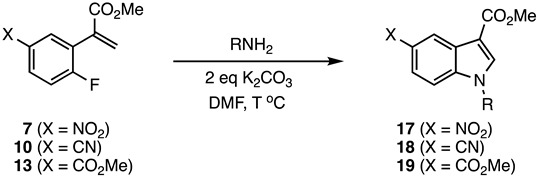
C5-Substituted 1-alkyl-1*H*-indole-3-carboxylate esters prepared.

Substrate	R	T (°C)	Product	Yield
**7**	–*n*-C_6_H_13_	23	**17a**	80
**7**	*–*CH_2_CH(CH_3_)_2_	23	**17b**	88
**7**	–CH_2_CH = CH_2_	23	**17c**	90
**7**	–*c*-C_3_H_5_	23	**17d**	75
**7**	–*c*-C_6_H_11_	23	**17e**	78
**7**	–C(CH_3_)_3_	23	**17f**	77
**7**	–CH_2_CH_2_C_6_H_5_	23	**17g**	85
**7**	–CH_2_C_6_H_5_	23	**17h**	85
**7**	–CH_2_C_6_H_4_-4-Me	23	**17i**	86
**7**	–CH_2_C_6_H_4_-4-Me	23	**17j**	83
**7**	–CH_2_C_6_H_4_-4-Me	23	**17k**	87
**7**	–CH_2_C_6_H_4_-4-Cl	23	**17l**	81
**7**	–CH_2_C_6_H_4_-4-CF_3_	23	**17m**	89
**7**	–CH_2_C_6_H_4_-3-NO_2_	23	**17n**	91
**7**	–NH_2_	23	**17o**	83
**10**	–*n*-C_6_H_13_	23	**18a**	74
**10**	*–*CH_2_CH(CH_3_)_2_	23	**18b**	67
**10**	–*c*-C_3_H_5_	23	**18d**	78
**10**	–*c*-C_6_H_11_	23	**18e**	79
**10**	–CH_2_CH_2_C_6_H_5_	23	**18g**	91
**10**	–CH_2_C_6_H_5_	23	**18h**	88
**10**	–CH_2_C_6_H_4_-4-Me	23	**18i**	81
**10**	–CH_2_C_6_H_4_-3-OMe	23	**18j**	83
**10**	–CHC_6_H_4_-4-OMe	23	**18k**	89
**10**	–CH_2_C_6_H_4_-4-Cl	23	**18l**	87
**10**	–CH_2_C_6_H_4_-3-NO_2_	23	**18n**	92
**13**	–*n*-C_6_H_13_	90	**19a**	67
**13**	*–*CH_2_CH(CH_3_)_2_	90	**19b**	69
**13**	–CH_2_CH = CH_2_	50	**19c**	80
**13**	–*c*-C_3_H_5_	90	**19d**	61
**13**	–*c*-C_6_H_11_	90	**19e**	65
**13**	–CH_2_CH_2_C_6_H_5_	90	**19g**	89
**13**	–CH_2_C_6_H_5_	50	**19h**	86
**13**	–CH_2_C_6_H_4_-4-Me	50	**19i**	82
**13**	–CH_2_C_6_H_4_-3-OMe	50	**19j**	86
**13**	–CH_2_C_6_H_4_-4-OMe	50	**19k**	86
**13**	–CH_2_C_6_H_4_-4-Cl	50	**19l**	80
**13**	–CH_2_C_6_H_4_-3-NO_2_	50	**19n**	91

## Data Availability

Not applicable.

## Supplementary Materials

The following supporting information can be downloaded at https://www.mdpi.com/article/10.3390/molecules27206998/s1, copies of the ^1^H-NMR and ^13^C-NMR spectra for all new compounds.

## References

[1] Navriti N., Silakari O. (2017). Indoles as therapeutics of interest in medicinal chemistry: Bird’s eye view. Eur. J. Med. Chem..

[2] Sravanthi T.V., Manju S.I. (2016). Indoles–A promising scaffold for drug development. Eur. J. Pharm. Sci..

[3] Fleming I., Woolias M. (1979). A benzyne route to indoles from *o*- or *m*-bromoaryl ketones. J. Chem. Soc. Perkin Trans. 1.

[4] Ito Y., Kobayashi K., Saegusa T. (1977). An efficient synthesis of indole. J. Am. Chem. Soc..

[5] Scriven E.F.V., Turnbull K. (1988). Azides: Their preparation and synthetic uses. Chem. Rev..

[6] Hegedus L.S., Allen G.F., Bozell J.J., Waterman E.L. (1978). Palladium-assisted intramolecular amination of olefins. Synthesis of nitrogen heterocycles. J. Am. Chem. Soc..

[7] Organic Chemistry Portal: Synthesis of Indoles. https://www.organic-chemistry.org/synthesis/heterocycles/benzofused/indolesshtm.

[8] Tilstam U., Harre M., Heckrodt T., Weinmann H. (2001). A mild efficient dehydrogenation of indolines. Tetrahedron Lett..

[9] Karki M., Araujo H.C., Magolan J. (2013). Dehydroaromatization with V_2_O_5_. Synth. Lett..

[10] Gribble G.W. (2016). Indolines to indoles by functionalized elimination in indole ring synthesis. Indole Ring Synthesis: From Natural Products to Drug Discovery.

[11] Caddick S., Aboutayab K., Jenkins K., West R.I. (1996). Intramolecular radical substitution reactions: A novel approach to fused [1,2-*a*]-indoles. J. Chem. Soc., Perkin Trans. 1.

[12] Giles P.R., Rogers-Evans M., Soukup M., Knight J. (2003). An improved process for the *N*-alkylation of indoles using chiral *N*-protected 2-methylaziridines. Org. Proc. Res. Dev..

[13] Bayindir S., Erdogan E., Kilic H., Aydin O., Saracoglu N. (2015). Synthesis of *N*-alkylated indolines and indoles from indoline and aliphatic ketones. J. Heterocycl. Chem..

[14] Ling L., Cao J., Hu J., Zhang H. (2017). Copper-catalyzed N-alkylation of indoles by *N*-tosylhydrazones. RSC Adv..

[15] Sun L., Zhang X., Wang C., Teng H., Ma J., Li M., Chen H., Jiang H. (2019). Direct electrosynthesis of *N*-alkyl-C3-haloindoles using alkyl halide as both alkylating and halogenating building blocks. Green Chem..

[16] Augustine R.L., Gustavsen A.J., Wanat S.F., Pattison I.C., Houghton K.S., Koletar G. (1973). Synthesis of α-monosubstituted indoles. J. Org. Chem..

[17] Bunce R.A., Randall M.H., Applegate K.G. (2002). 2-Alkylindole-3-carboxylate esters by a tandem reduction-addition-elimination reaction. Org. Prep. Proced. Int..

[18] D’Ambra T.E., Estep K.G., Bell M.R., Eissenstat M.A., Josef K.A., Ward S.J., Haycock D.A., Baizman E.R., Casiano F.M., Beglin N.C. (1992). Conformationally restrained analogs of pravadoline: Nanomolar potent, enantioselective, (aminoalkyl) indole agonists of the cannabinoid receptor. J. Med. Chem..

[19] Sun Y., Alexander S.P.H., Garle M.J., Gibson C.L., Hewitt K., Murphy S.P., Kendall D.A., Bennett A.J. (2007). Cannabinoid activation of PPARa; a novel neuroprotective mechanism. Br. J. Pharmacol..

[20] Smith V. (2008). Synthesis and pharmacology of N-alkyl-3-halonaphthoyl)indoles. All Diss.

[21] Boriskin Y.S., Leneva I.A., Pécheur E.-I., Polyak S.J. (2008). Arbidol: A broad-spectrum antiviral compound that blocks viral fusion. Curr. Med. Chem..

[22] Liu M. (2020). COVID-19 pandemic: Case studies and perspectives. Side Effects Annual.

[23] Behbehani H., Ibrahim H.M., Makhseed S., Mahmoud H. (2011). Applications of 2-arylhydrazononitriles in synthesis: Preparation of new indole containing 1,2,3-triazole, pyrazole and pyrazolo[1,5-*a*]pyrimidine derivatives and evaluation of their antimicrobial activities. Eur. J. Med. Chem..

[24] Gale D.J., Wilshire J.F.K. (1970). The preparation of sole polymethine astrazon dyes. Aust. J. Chem..

[25] Selvakumar N., Azhagan A.M., Srinivas D., Krishna G.G. (2002). A direct synthesis of 2-arylpropenoic acid esters having nitro in the aromatic ring: A short synthesis of (±)-coerulescine and (±)-horsfiline. Tetrahedron Lett..

[26] Zhao G., Souers A.J., Voorbach M., Falls H.D., Droz B., Brodjian S., Lau Y.Y., Iyengar R.R., Gao J., Judd A.S. (2008). Validation of diacyl glycerolacyltransferase I as a novel target for the treatment of obesity and dyslipidemia using a potent and selective small molecule inhibitor. J. Med. Chem..

[27] Clarke H.T., Read R.R., Gilman H., Blatt A.H. (1941). *o*-Tolunitrile and *p*-tolunitrile. Organic Syntheses, Collective Volume 1.

[28] Marvel C.S., McElvain S.M., Gilman H., Blatt A.H. (1941). *o*-Chlorotoluene and *p*-chlorotoluene. Organic Syntheses, Collective Volume 1.

[29] Bunce R.A., Rogers D., Nago T., Bryant S.A. (2008). 4*H*-Benzopyrans by a tandem S_N_2-S_N_Ar reaction. J. Heterocycl. Chem..

[30] Bunce R.A., Nago T., Sonobe N., Slaughter L.M. (2008). Benzo-fused heterocycles and carbocycles by intramolecular S_N_Ar and tandem S_N_2-S_N_Ar reactions. J. Heterocycl. Chem..

[31] Grossert J.S., Dubey P.K., Gill G.H., Cameron S., Gardner P.A. (1984). The preparation, spectral properties, structures and base-induced cleavage reactions of some α-halo-β-ketosulfones. Can. J. Chem..

[32] Smith M.B., March J. (2007). March’s Advanced Organic Chemistry Reactions Mechanisms and Structure.

[33] Carey F.A., Sundberg R.J. (2000). Advanced Organic Chemistry Part A: Structure and Mechanisms.

[34] Achord J.M., Hussey C.L. (1980). Determination of dissolved oxygen in nonaqueous electrochemical solvents. Anal. Chem..

[35] Annor-Gyamfi J.K., Ametsetor E., Meraz K., Bunce R.A. (2021). Dihydroquinolines, dihydronaphthyridines and quinolones by domino reactions of Morita-Baylis-Hillman acetates. Molecules.

