# Supplementary material for: Domino Aza-Michael-SNAr-Heteroaromatization Route to C5-Substituted 1-Alkyl-1H-Indole-3-Carboxylic Esters
Source: Molecules. 2022 Oct 18;27(20):6998. doi: 10.3390/molecules27206998 (PMC9611145; doi:10.3390/molecules27206998)

Domino aza-Michael Addition-SNAr-Heteroaromatization Route to C5-Substituted 1-Alkyl-1*H*-indole-3-carboxylic Esters

Ebenezer Ametsetor, Spencer Farthing and Richard A. Bunce*

Department of Chemistry, Oklahoma State University, Stillwater, OK 74078-3071, USA
*Correspondence: rab@okstate.edu; Tel: +1-405-744-5952

Supplementary Information

Spectral Data **Page**

^{1}H and ^{13}C NMR for methyl 2-(5-cyano-2-fluorophenyl)acetate (**9**) 3
^{1}H and ^{13}C NMR for methyl 2-(5-nitro-2-fluorophenyl)acrylate (**7**) 4
^{1}H and ^{13}C NMR for methyl 2-(5-cyano-2-fluorophenyl)acrylate (**10**) 5
^{1}H and ^{13}C NMR for methyl 2-(5-carbomethoxy-2-fluorophenyl)acrylate (**13**) 6
^{1}H and ^{13}C NMR for methyl 1-hexyl-5-nitro-1*H*-indole-3-carboxylate (**17a**) 7
^{1}H and ^{13}C NMR for methyl 1-isobutyl-5-nitro-1*H*-indole-3-carboxylate (**17b**) 8
^{1}H and ^{13}C NMR for methyl 1-allyl-5-nitro-1*H*-indole-3-carboxylate (**17c**) 9
^{1}H and ^{13}C NMR for methyl 1-cyclopropyl-5-nitro-1*H*-indole-3-carboxylate (**17d**) 10
^{1}H and ^{13}C NMR for methyl 1-cyclohexyl-5-nitro-1*H*-indole-3-carboxylate (**17e**) 11
^{1}H and ^{13}C NMR for methyl 1-(*tert*-butyl)-5-nitro-1*H*-indole-3-carboxylate (**17f**) 12
^{1}H and ^{13}C NMR for methyl 5-nitro-1-phenethyl-1*H*-indole-3-carboxylate (**17g**) 13
^{1}H and ^{13}C NMR for methyl 1-benzyl-5-nitro-1*H*-indole-3-carboxylate (**17h**) 14
^{1}H and ^{13}C NMR for methyl 1-(4-methylbenzyl)-5-nitro-1*H*-indole-3-carboxylate (**17i**) 15
^{1}H and ^{13}C NMR for methyl 1-(3-methoxybenzyl)-5-nitro-1*H*-indole-3-carboxylate (**17j**) 16
^{1}H and ^{13}C NMR for methyl 1-(4-methoxybenzyl)-5-nitro-1*H*-indole-3-carboxylate (**17k**) 17
^{1}H and ^{13}C NMR for methyl 1-(4-chlorobenzyl)-5-nitro-1*H*-indole-3-carboxylate (**17l**) 18
^{1}H and ^{13}C NMR for methyl 5-nitro-1-((4-trifluoromethyl)benzyl)-1*H*-indole-3-carboxylate (**17m**) 19
^{1}H and ^{13}C NMR for methyl 5-nitro-1-(3-nitrobenzyl)-1*H*-indole-3-carboxylate (**17n**) 20
^{1}H and ^{13}C NMR for methyl 1-amino-5-nitro-1*H*-indole-3-carboxylate (**17o**) 21

^{1}H and ^{13}C NMR for methyl 5-cyano-1-hexyl-1*H*-indole-3-carboxylate (**18a**) 22
^{1}H and ^{13}C NMR for methyl 5-cyano-1-isobutyl-1*H*-indole-3-carboxylate (**18b**) 23
^{1}H and ^{13}C NMR for methyl 5-cyano-1-cyclopropyl-1*H*-indole-3-carboxylate (**18d**) 24
^{1}H and ^{13}C NMR for methyl 5-cyano-1-cyclohexyl-1*H*-indole-3-carboxylate (**18e**) 25
^{1}H and ^{13}C NMR for methyl 5-cyano-1-phenethyl-1*H*-indole-3-carboxylate (**18g**) 26
^{1}H and ^{13}C NMR for methyl 5-cyano-1-benzyl-1*H*-indole-3-carboxylate (**18h**) 27
^{1}H and ^{13}C NMR for methyl 5-cyano-1-(4-methylbenzyl)-1*H*-indole-3-carboxylate (**18i**) 28
^{1}H and ^{13}C NMR for methyl 5-cyano-1-(3-methoxybenzyl)-1*H*-indole-3-carboxylate (**18j**) 29
^{1}H and ^{13}C NMR for methyl 5-cyano-1-(4-methoxybenzyl)-1*H*-indole-3-carboxylate (**18k**) 30
^{1}H and ^{13}C NMR for methyl 5-cyano-1-(4-chlorobenzyl)-1*H*-indole-3-carboxylate (**18l**) 31
^{1}H and ^{13}C NMR for methyl 5-cyano-1-(3-nitrobenzyl)-1*H*-indole-3-carboxylate (**18n**) 32

^{1}H and ^{13}C NMR for dimethyl 1-hexyl-1*H*-indole-3,5-dicarboxylate (**19a**) 33
^{1}H and ^{13}C NMR for dimethyl 1-isobutyl-1*H*-indole-3,5-dicarboxylate (**19b**) 34
^{1}H and ^{13}C NMR for dimethyl 1-allyl-1*H*-indole-3,5-dicarboxylate (**19c**) 35
^{1}H and ^{13}C NMR for dimethyl 1-cyclopropyl-1*H*-indole-3,5-dicarboxylate (**19d**) 36
^{1}H and ^{13}C NMR for dimethyl 1-cyclohexyl-1*H*-indole-3,5-dicarboxylate (**19e**) 37
^{1}H and ^{13}C NMR for dimethyl 1-phenethyl-1*H*-indole-3,5-dicarboxylate (**19g**) 38
^{1}H and ^{13}C NMR for dimethyl 1-benzyl-1*H*-indole-3,5-dicarboxylate (**19h**) 39
^{1}H and ^{13}C NMR for dimethyl 1-(4-methylbenzyl)-1*H*-indole-3,5-dicarboxylate (**19i**) 40
^{1}H and ^{13}C NMR for dimethyl 1-(3-methoxybenzyl)-1*H*-indole-3,5-dicarboxylate (**19j**) 41
^{1}H and ^{13}C NMR for dimethyl 1-(4-methoxybenzyl)-1*H*-indole-3,5-dicarboxylate (**19k**) 42
^{1}H and ^{13}C NMR for dimethyl 1-(4-chlorobenzyl)-1*H*-indole-3,5-dicarboxylate (**19l**) 43
^{1}H and ^{13}C NMR for dimethyl 1-(3-nitrobenzyl)-1*H*-indole-3,5-dicarboxylate (**19n**) 44

^{1}H for methyl 2-(5-cyano-2-fluorophenyl)acetate (**9**)

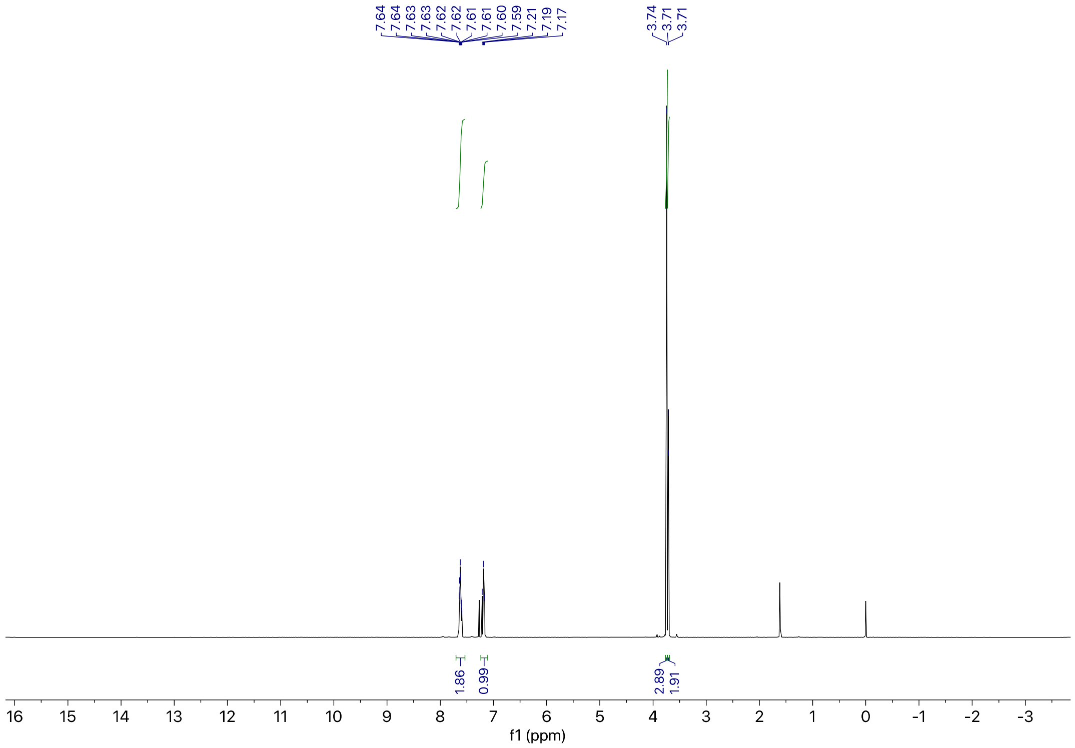

^{13}C for methyl 2-(5-cyano-2-fluorophenyl)acetate (**9**)

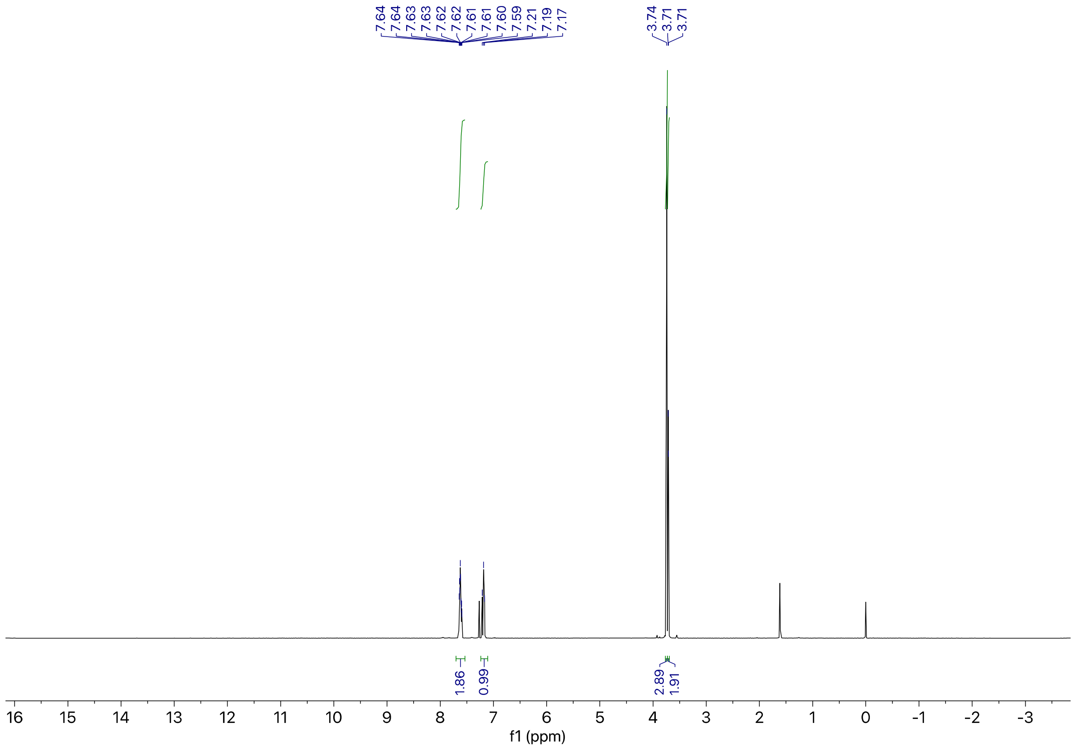

^{1}H NMR for methyl 2-(5-nitro-2-fluorophenyl)acrylate (**7**)

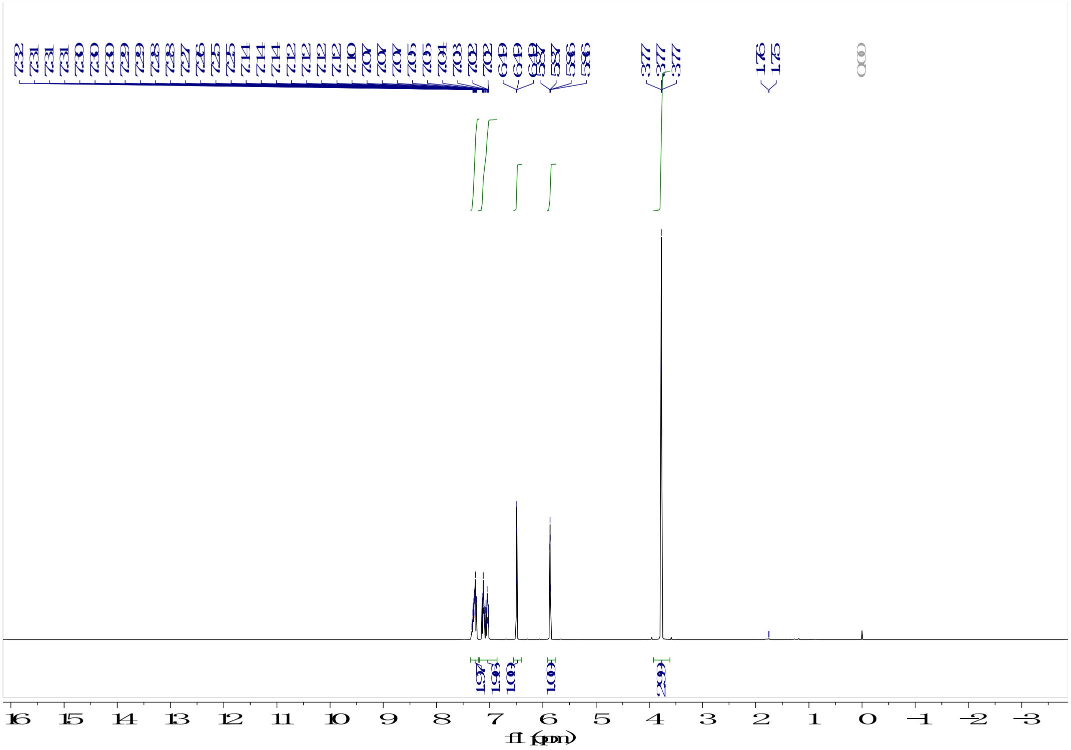

^{13}C NMR for methyl 2-(5-nitro-2-fluorophenyl)acrylate (**7**)

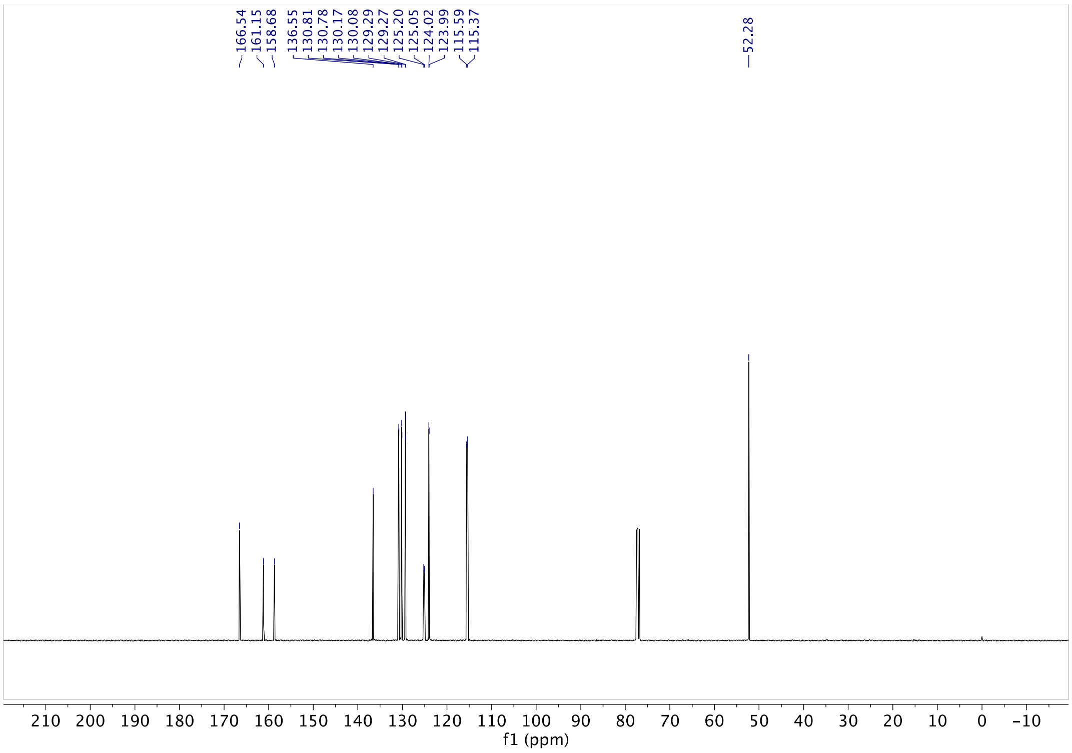

^{1}H NMR for methyl 2-(5-cyano-2-fluorophenyl)acrylate (**10**)

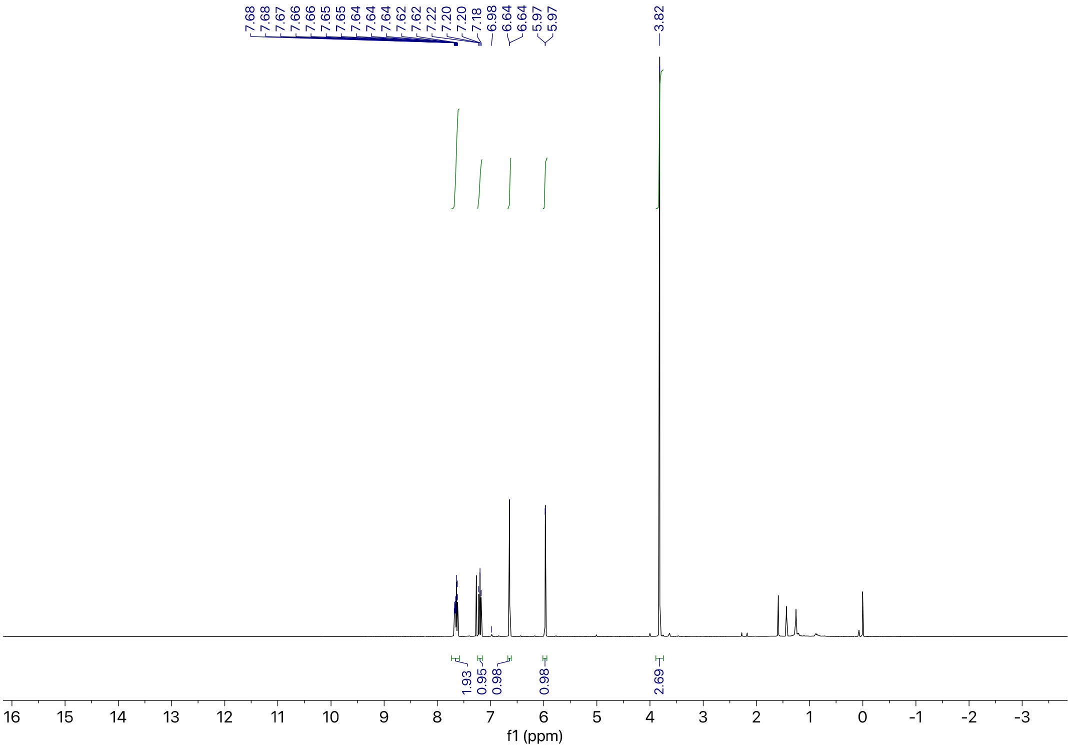

[13]C for methyl 2-(5-cyano-2-fluorophenyl)acrylate (**10**)

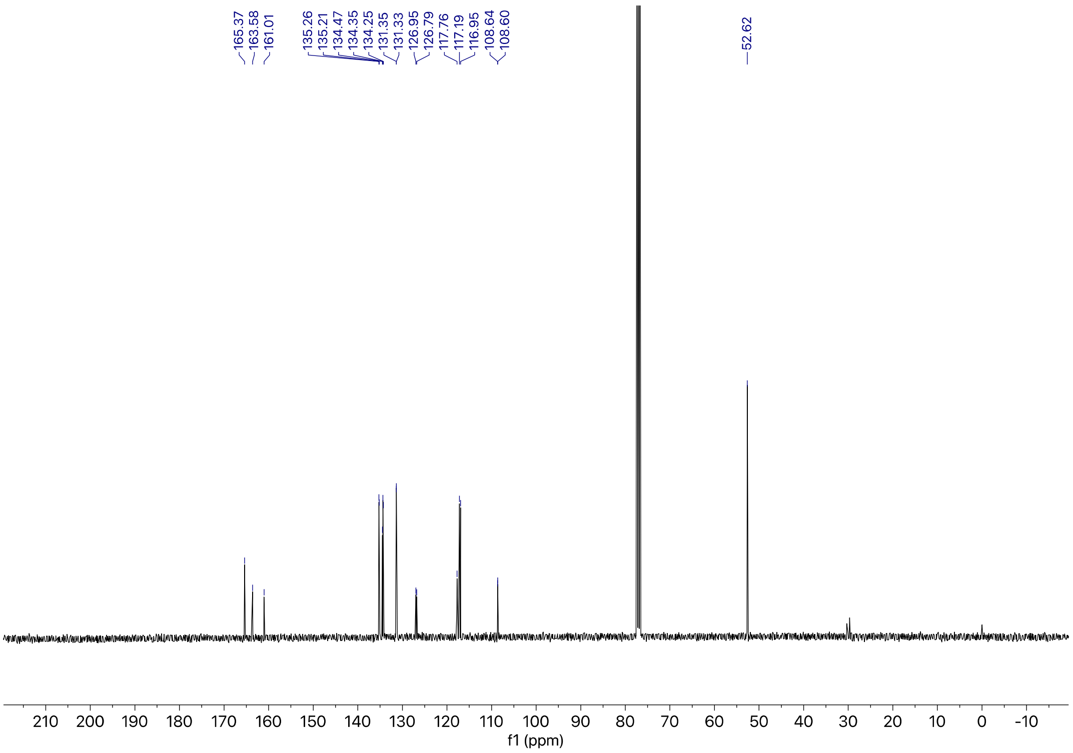

^{1}H NMR for methyl 2-(5-carbomethoxy-2-fluorophenyl)acrylate (**13**)

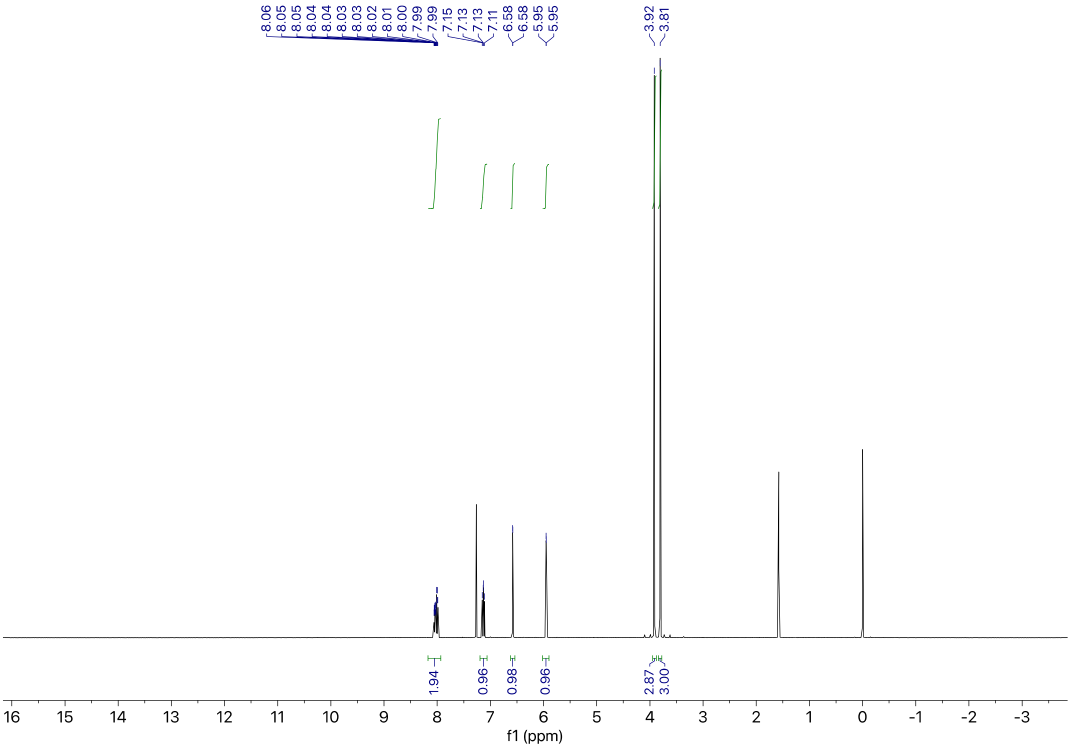

^{13}C NMR for methyl 2-(5-carbomethoxy-2-fluorophenyl)acrylate (**13**)

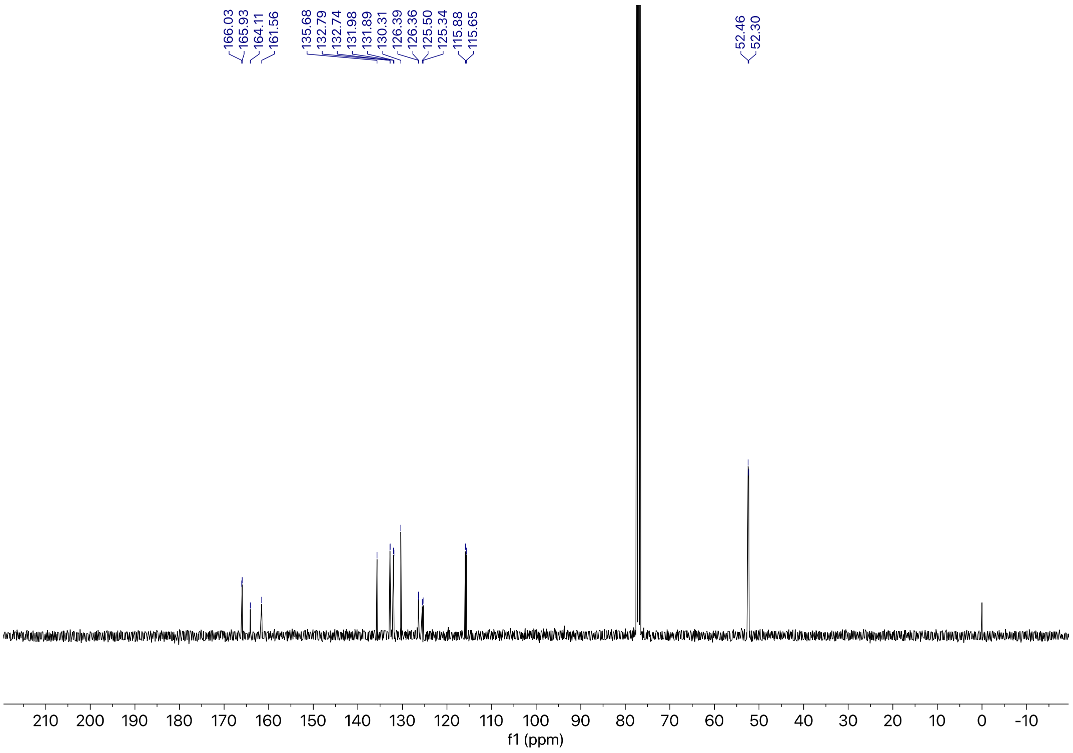

^{1}H NMR for methyl 1-hexyl-5-nitro-1*H*-indole-3-carboxylate (**17a**)

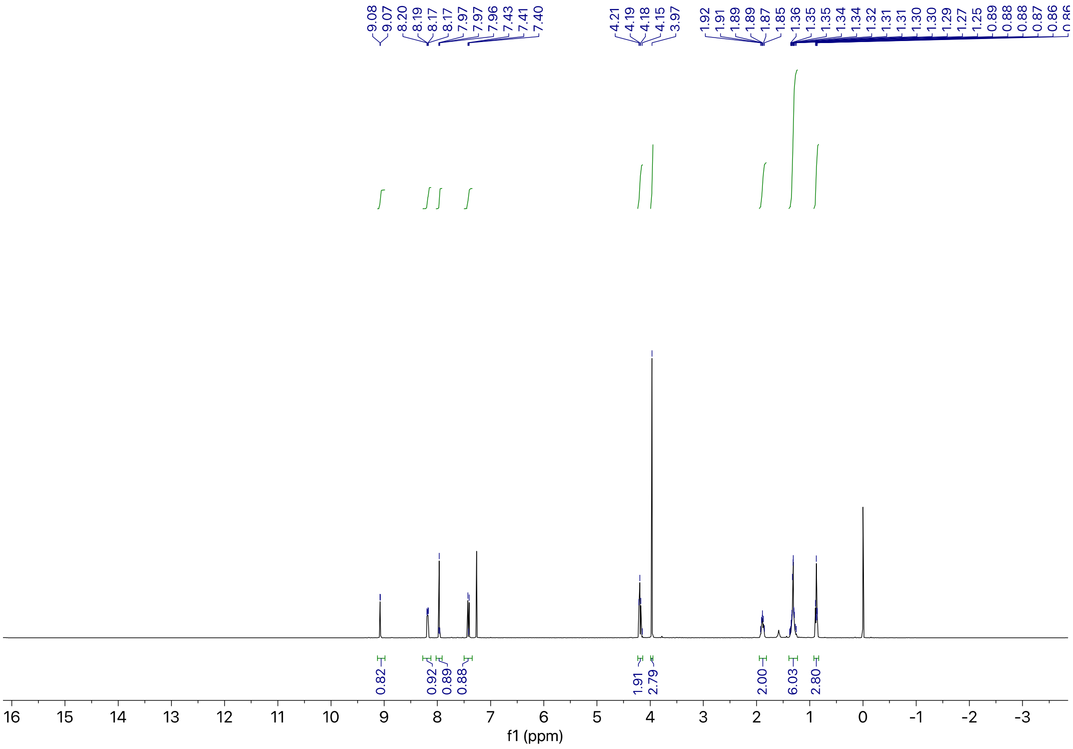

^{13}C NMR for methyl 1-hexyl-5-nitro-1*H*-indole-3-carboxylate (**17a**)

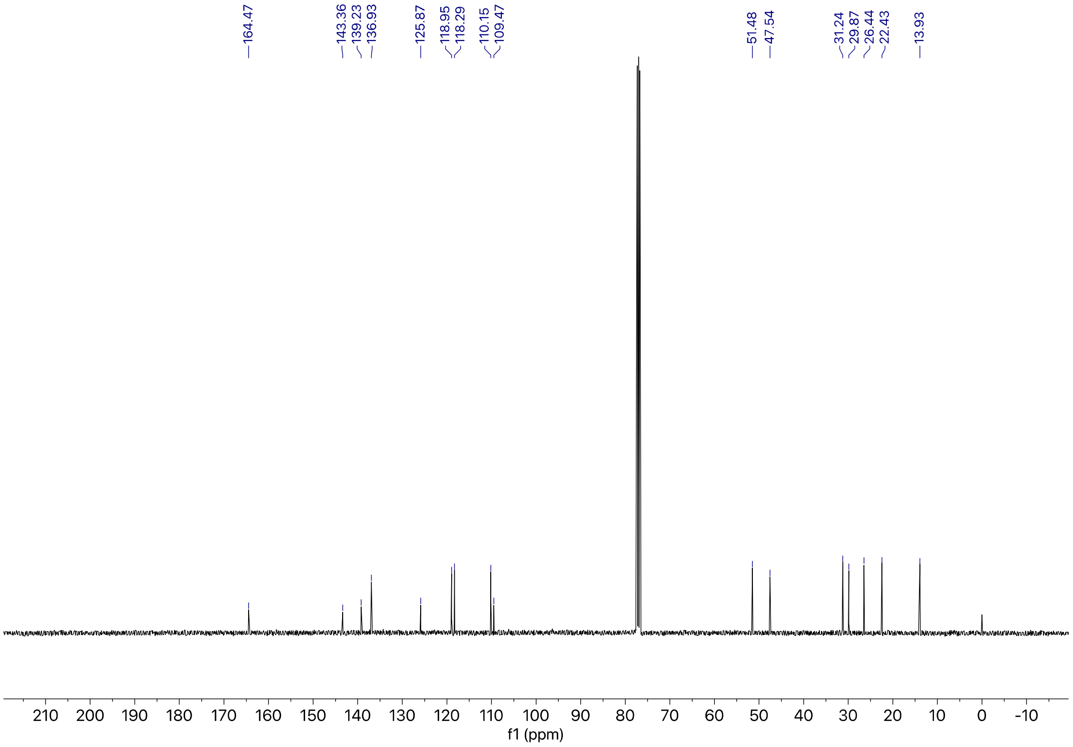

^{1}H NMR for methyl 1-isobutyl-5-nitro-1*H*-indole-3-carboxylate (**17b**)

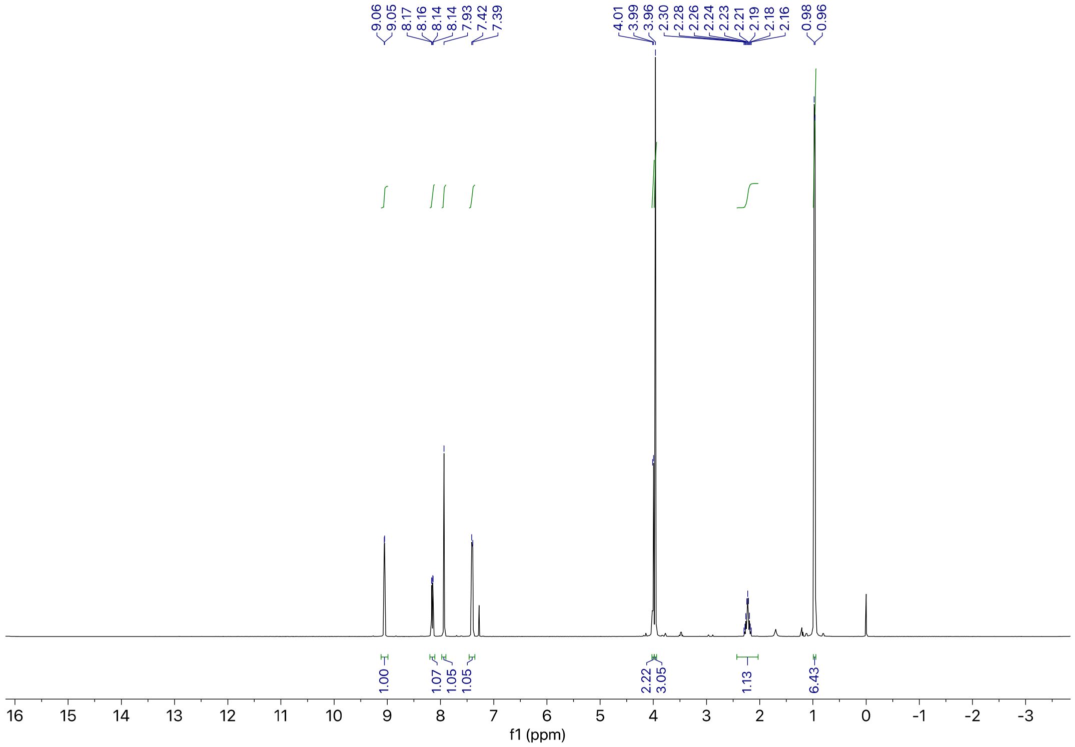

^{13}C NMR for methyl 1-isobutyl-5-nitro-1*H*-indole-3-carboxylate (**17b**)

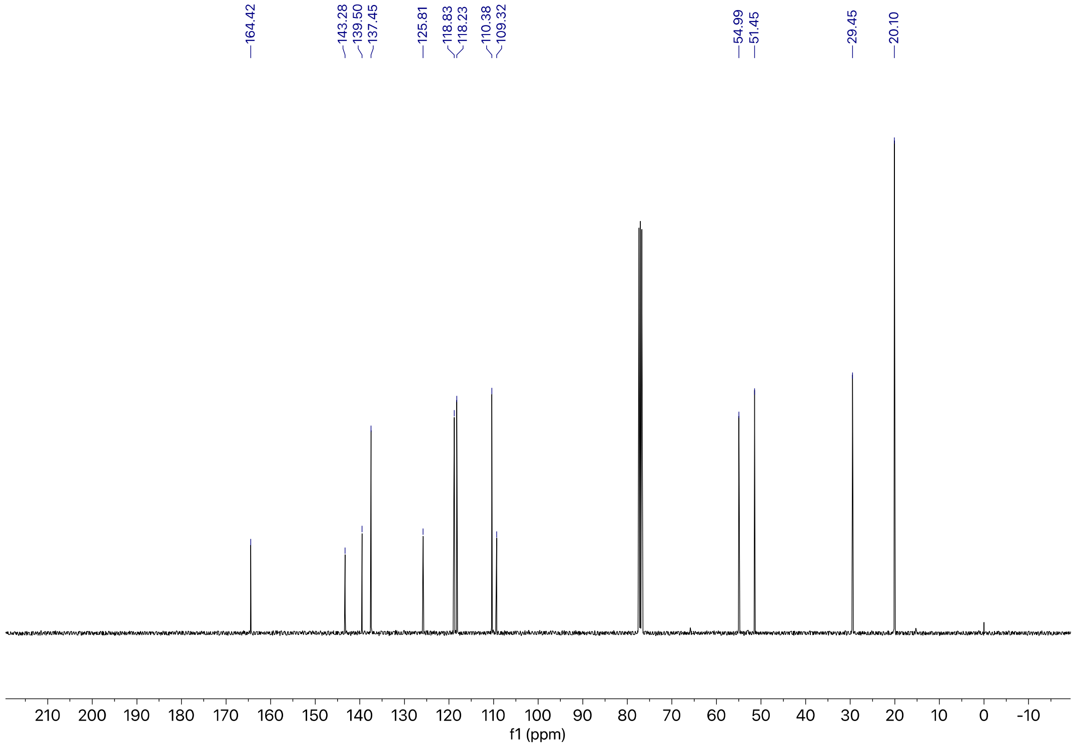

^{1}H NMR for methyl 1-allyl-5-nitro-1*H*-indole-3-carboxylate (**17c**)

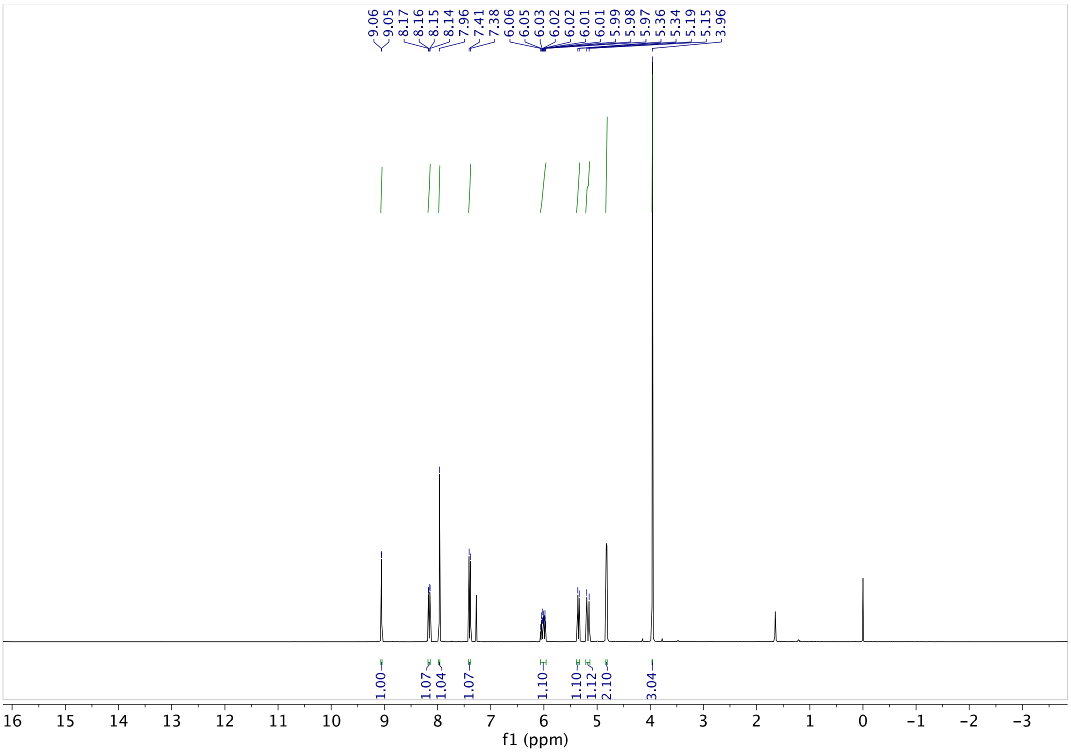

^{13}C NMR for methyl 1-allyl-5-nitro-1*H*-indole-3-carboxylate (**17c**)

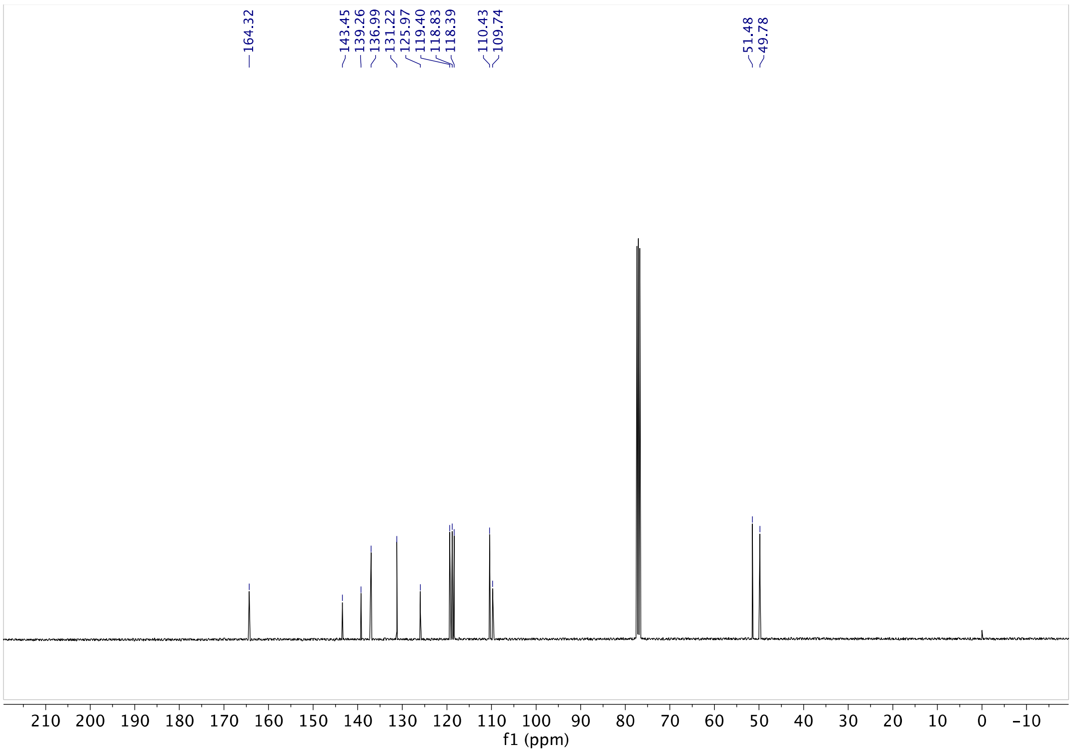

^{1}H NMR for methyl 1-cyclopropyl-5-nitro-1*H*-indole-3-carboxylate (**17d**)

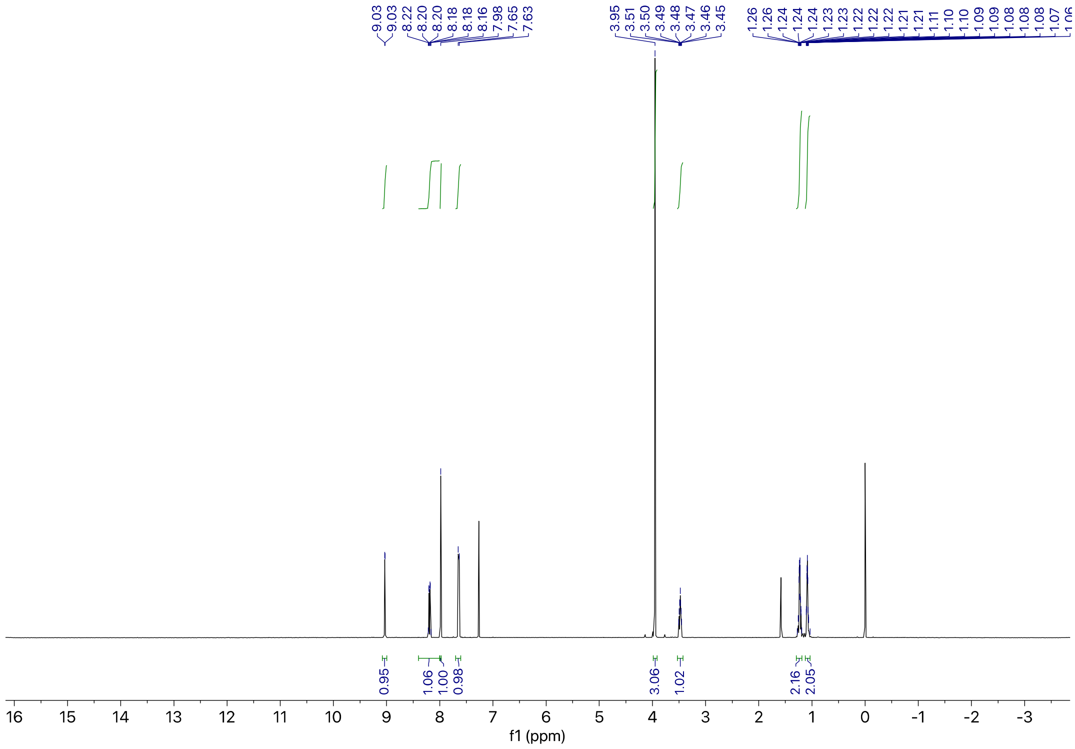

^{13}C NMR for methyl 1-cyclopropyl-5-nitro-1*H*-indole-3-carboxylate (**17d**)

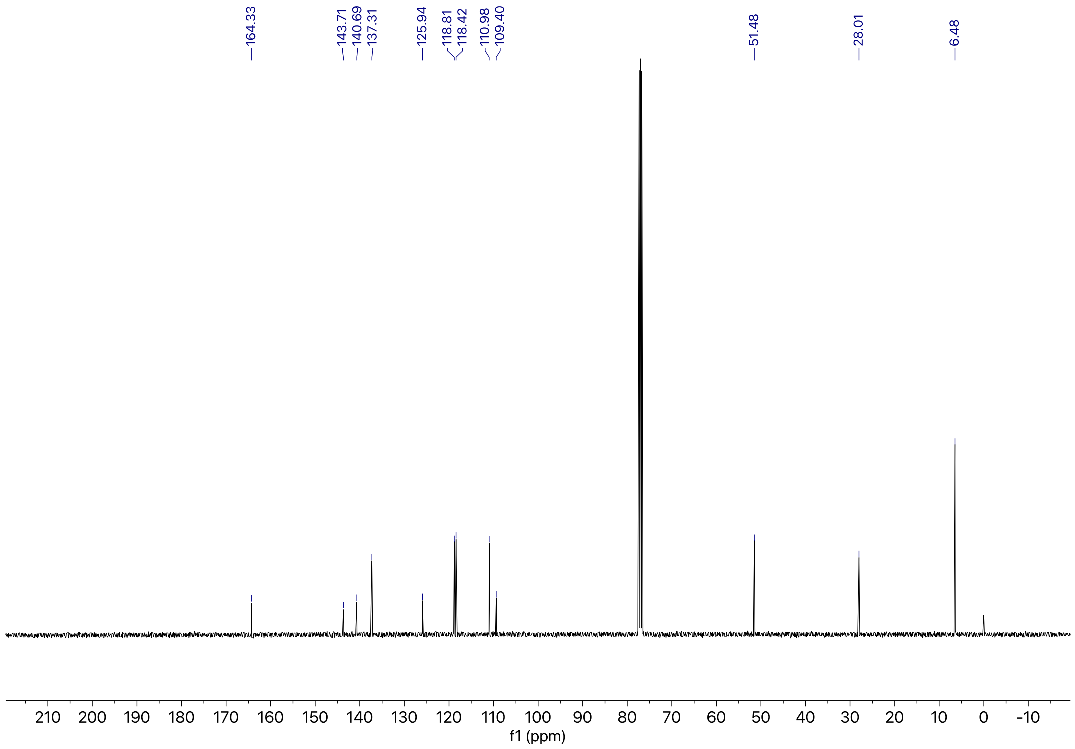

^{1}H NMR for methyl 1-cyclohexyl-5-nitro-1*H*-indole-3-carboxylate (**17e**)

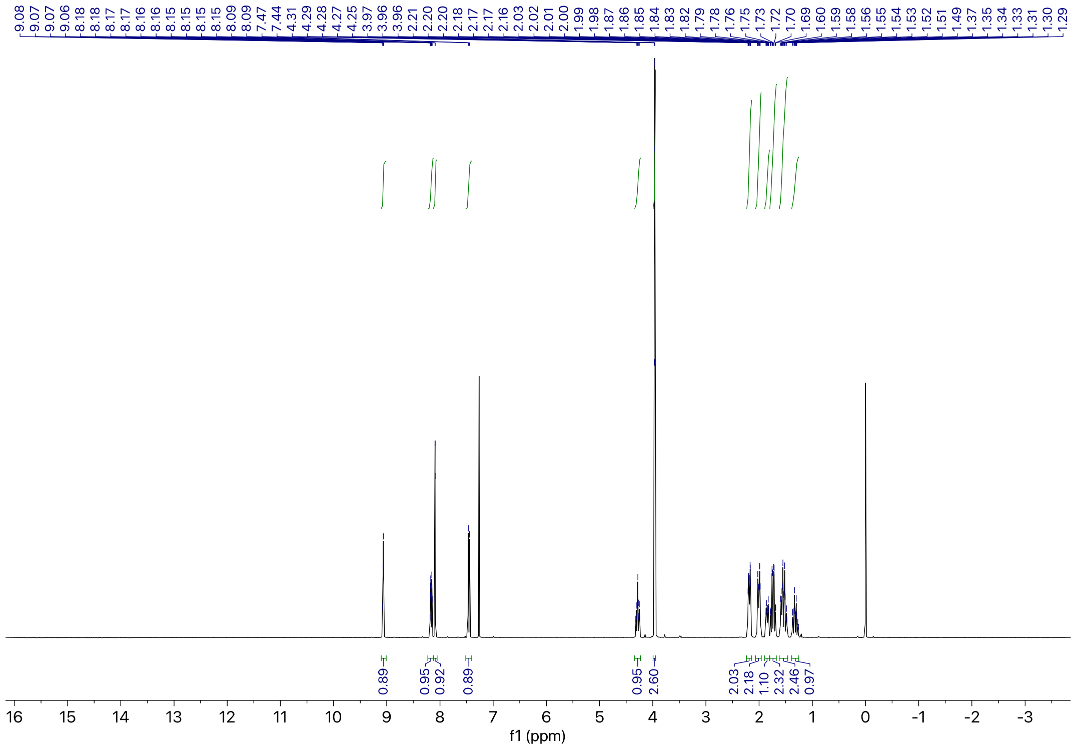

^{13}C NMR for methyl 1-cyclohexyl-5-nitro-1*H*-indole-3-carboxylate (**17e**)

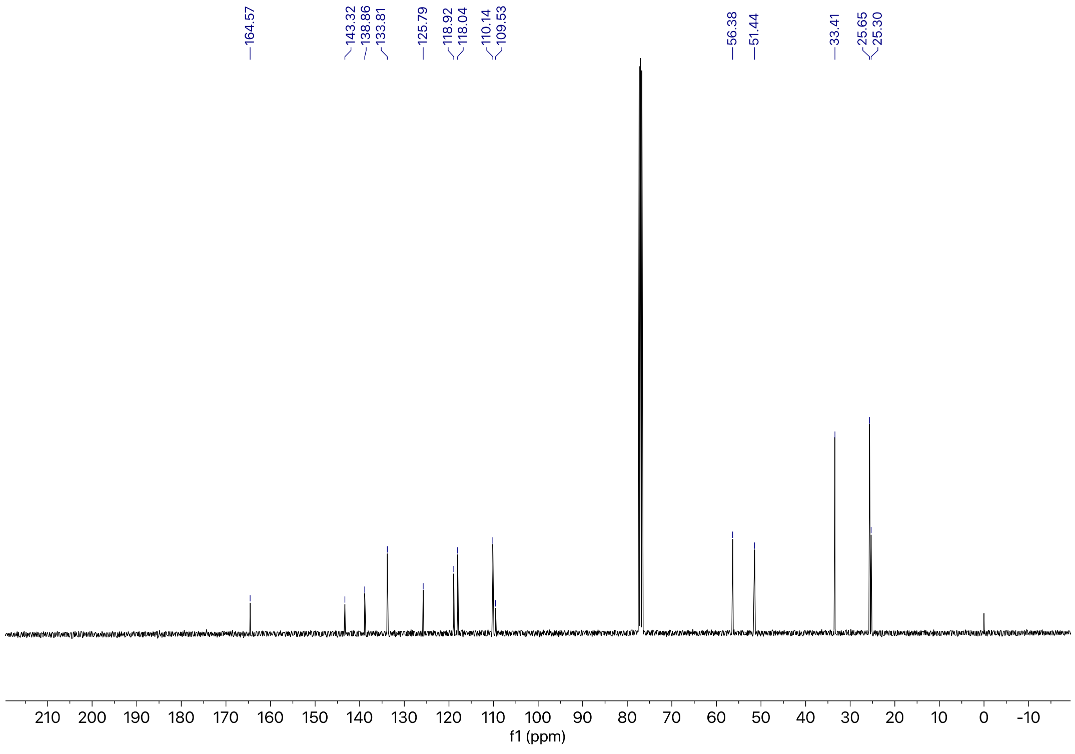

^{1}H NMR for methyl 1-(*tert*-butyl)-5-nitro-1*H*-indole-3-carboxylate (**17f**)

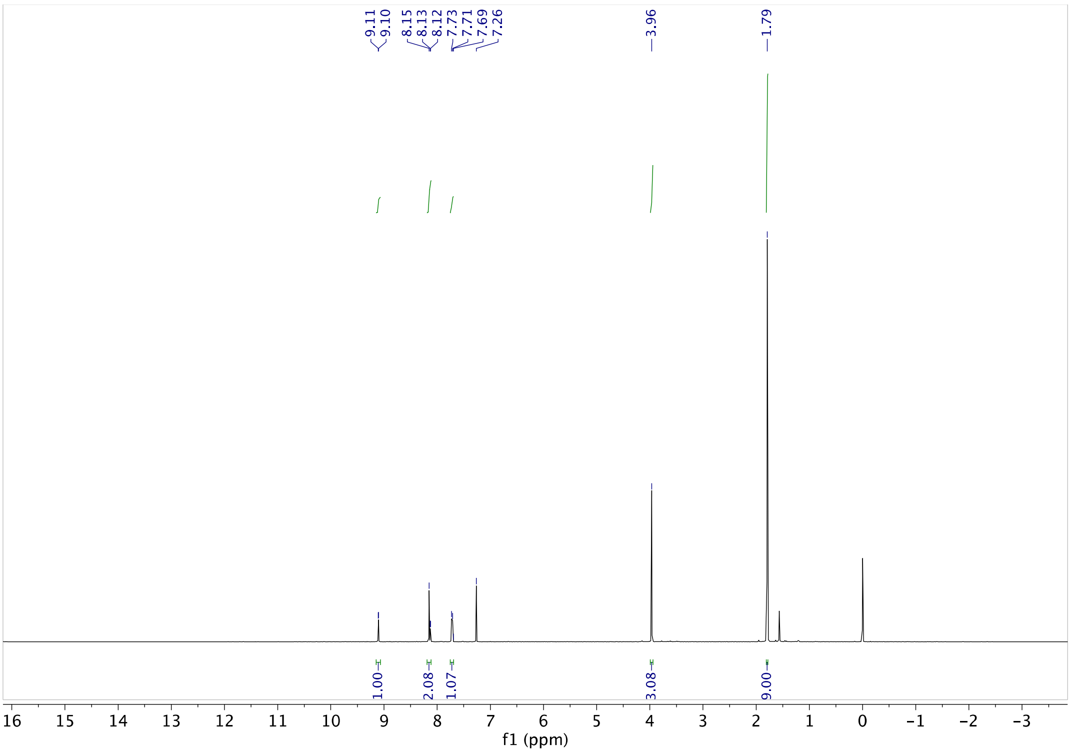

^{13}C NMR for methyl 1-(*tert*-butyl)-5-nitro-1*H*-indole-3-carboxylate (**17f**)

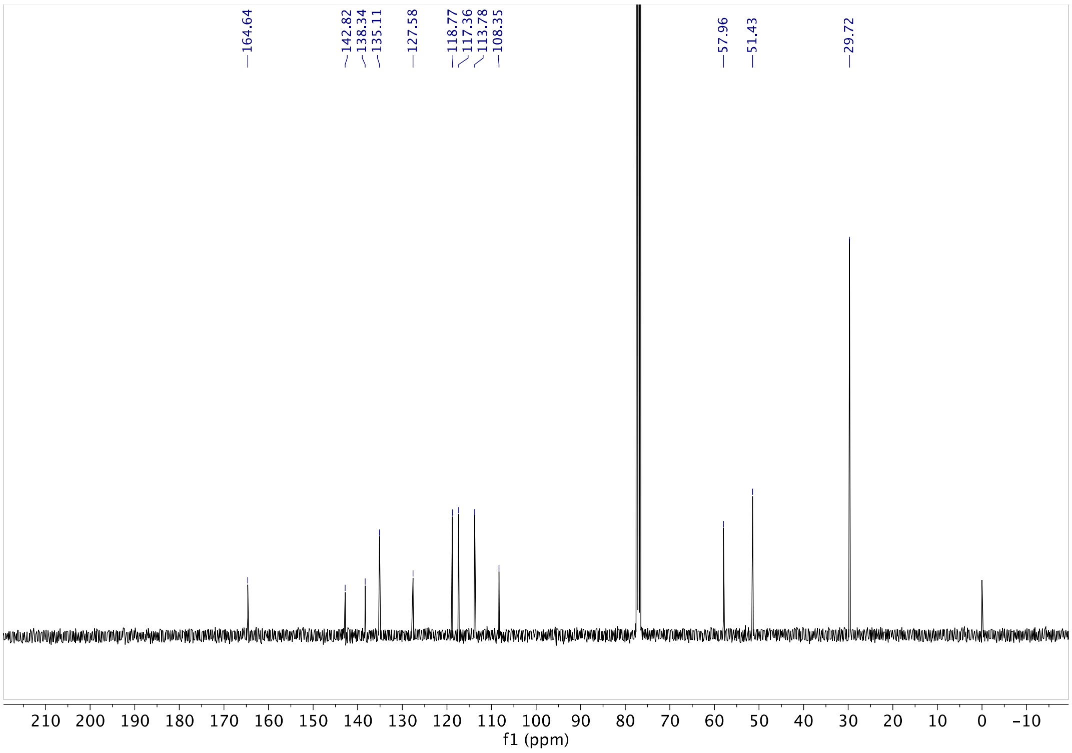

^{1}H NMR for methyl 5-nitro-1-phenethyl-1*H*-indole-3-carboxylate (**17g**)

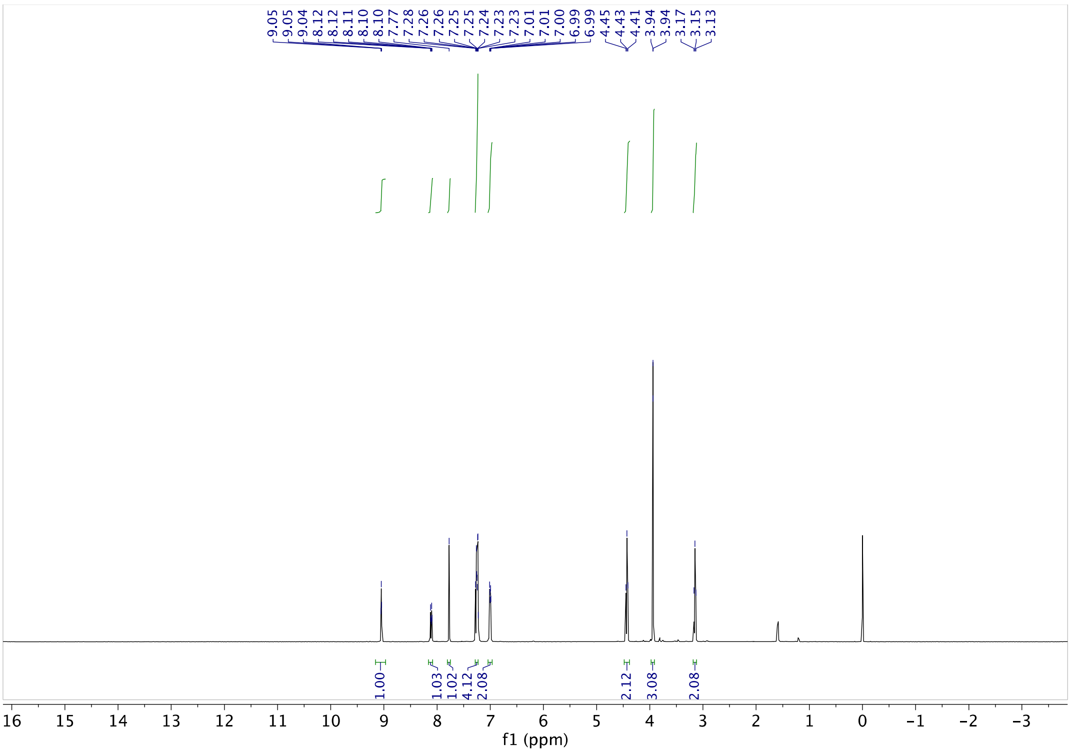

^{13}C NMR for methyl 5-nitro-1-phenethyl-1*H*-indole-3-carboxylate (**17g**)

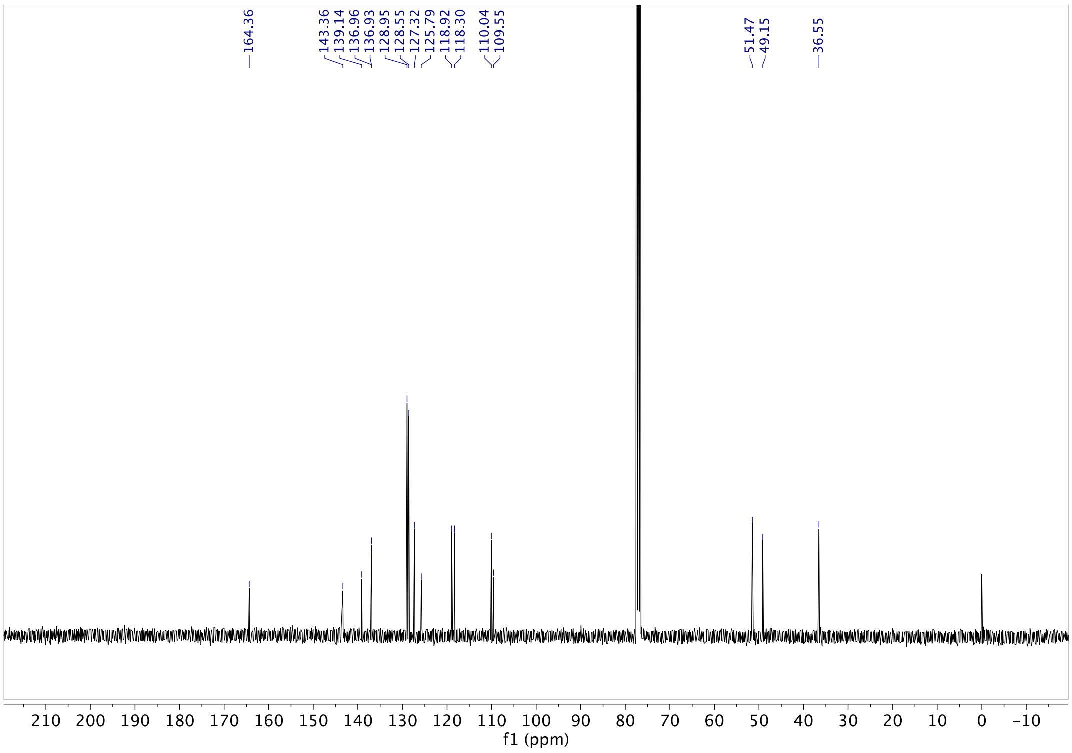

^{1}H NMR for methyl 1-benzyl-5-nitro-1*H*-indole-3-carboxylate (**17h**)

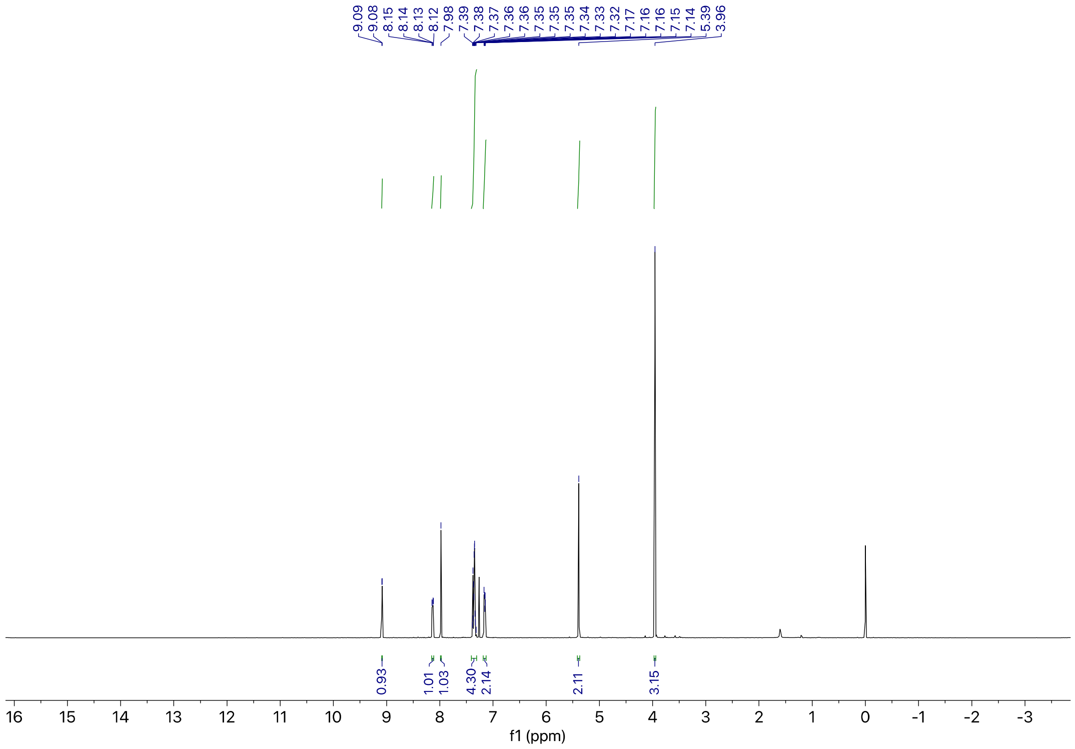

^{13}C NMR for methyl 1-benzyl-5-nitro-1*H*-indole-3-carboxylate (**17h**)

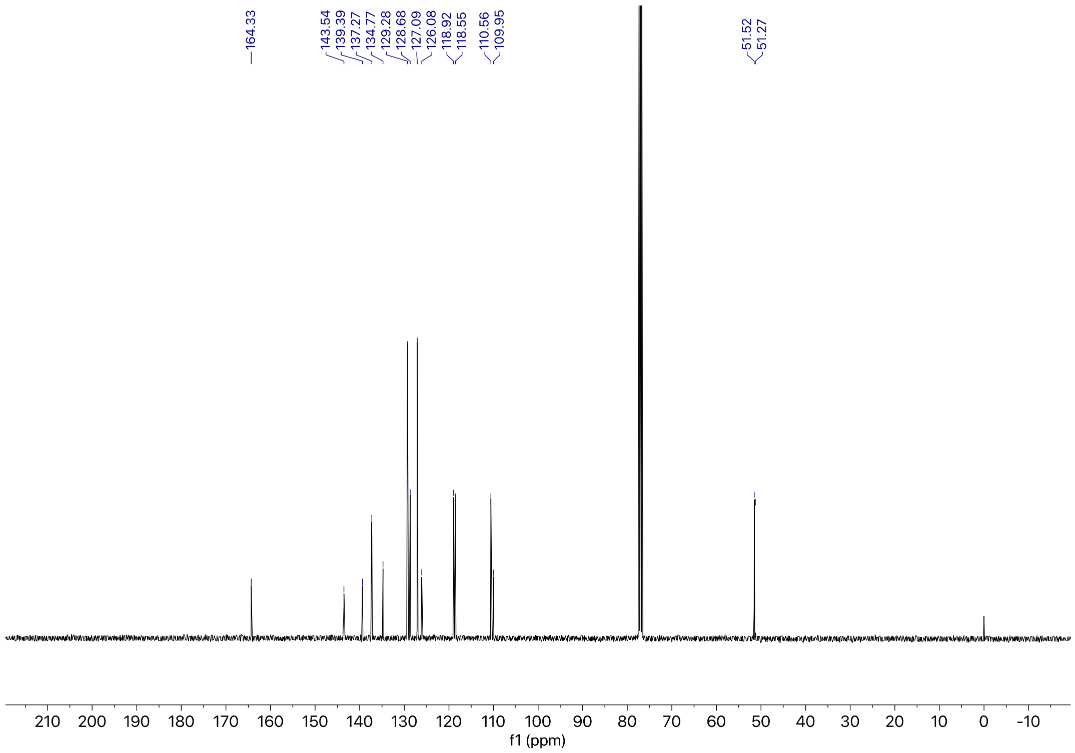

^{1}H NMR for methyl 1-(4-methylbenzyl)-5-nitro-1*H*-indole-3-carboxylate (**17i**)

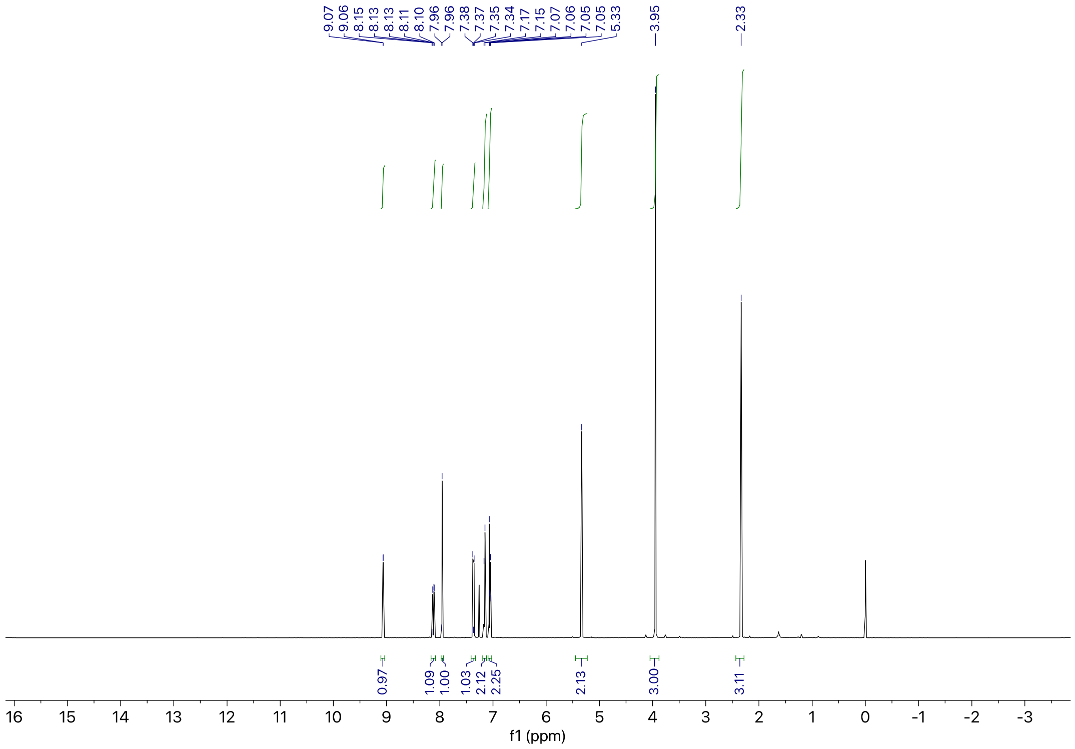

^{13}C NMR for methyl 1-(4-methylbenzyl)-5-nitro-1*H*-indole-3-carboxylate (**17i**)

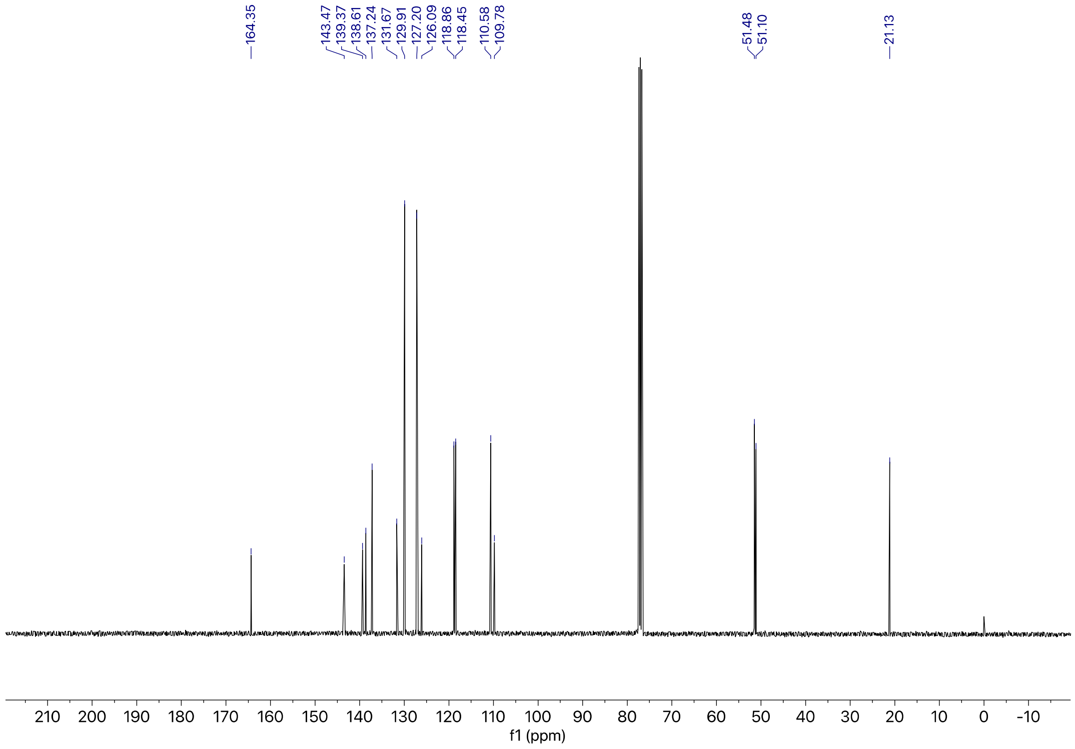

1H NMR for methyl 1-(3-methoxybenzyl)-5-nitro-1*H*-indole-3-carboxylate (**17j**)

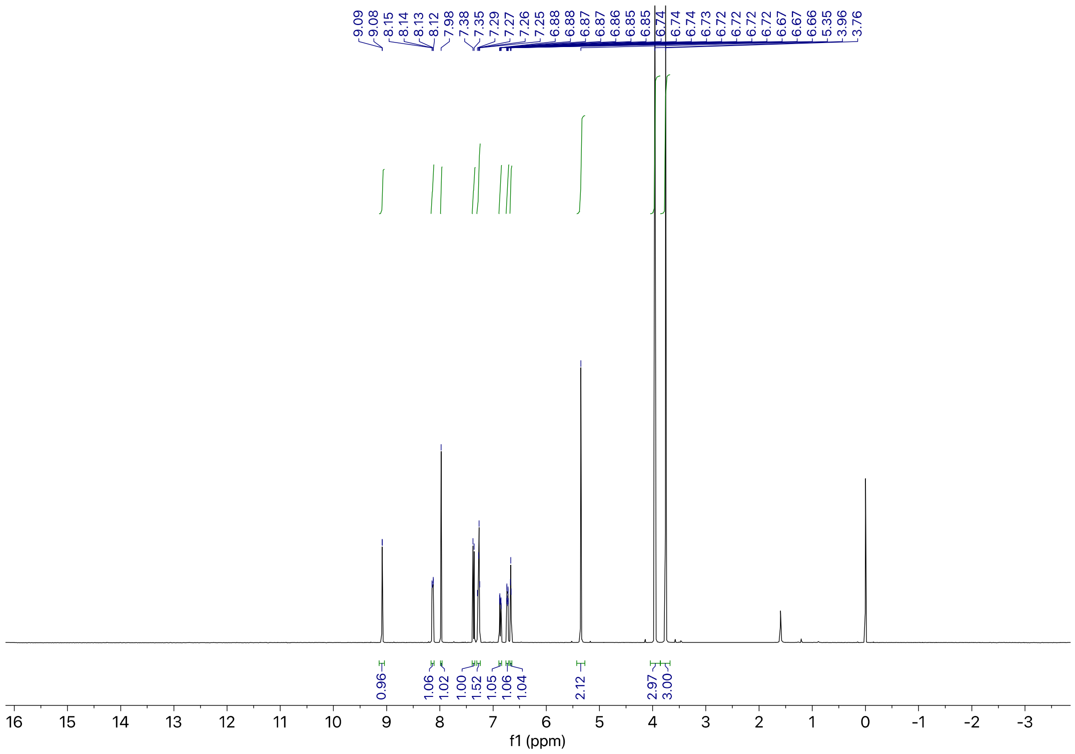

13C NMR for methyl 1-(3-methoxybenzyl)-5-nitro-1*H*-indole-3-carboxylate (**17j**)

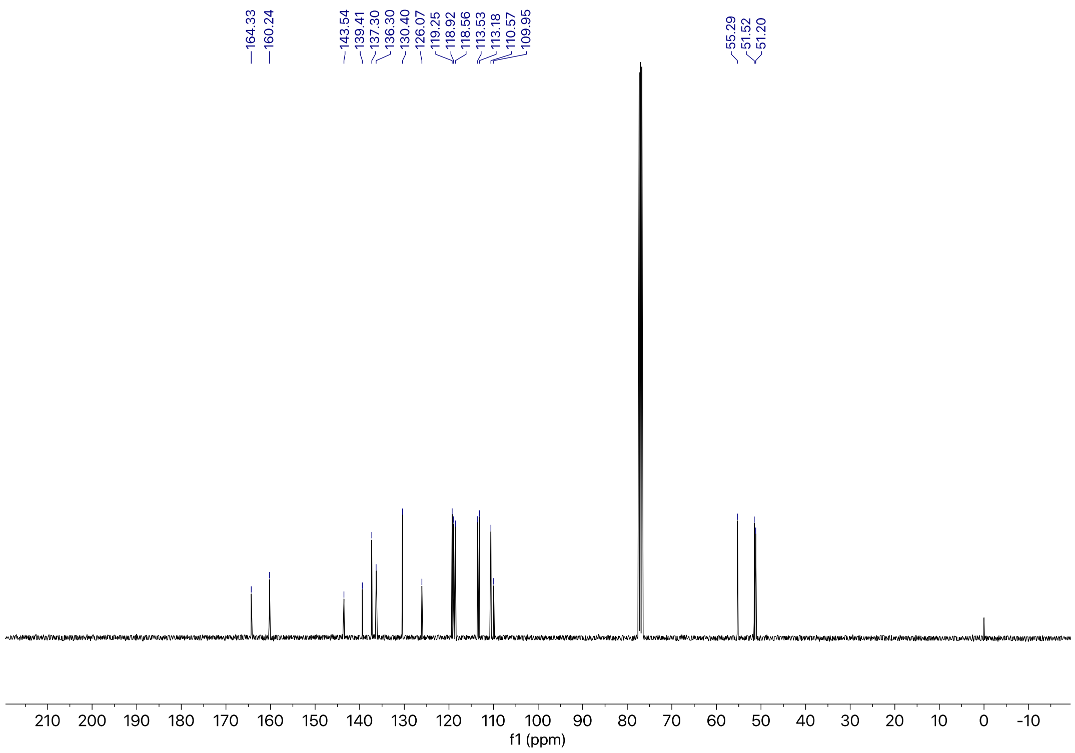

^{1}H NMR for Methyl 1-(4-methoxybenzyl)-5-nitro-1*H*-indole-3-carboxylate (**17k**)

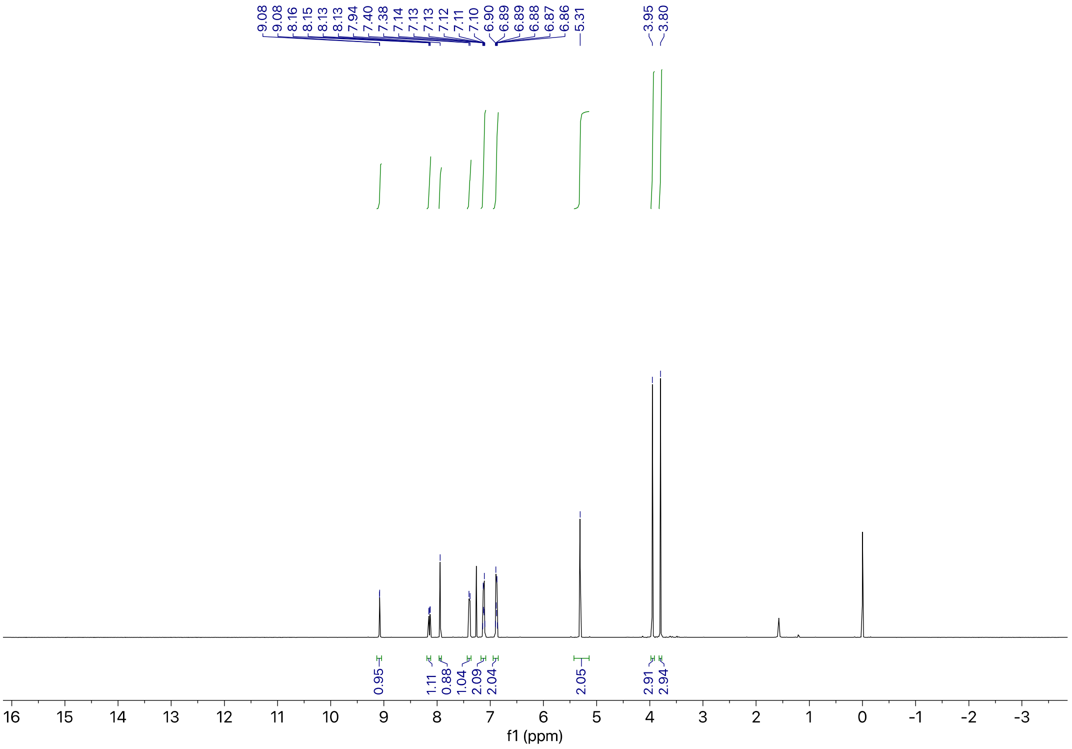

^{13}C NMR for methyl 1-(4-methoxybenzyl)-5-nitro-1*H*-indole-3-carboxylate (**17k**)

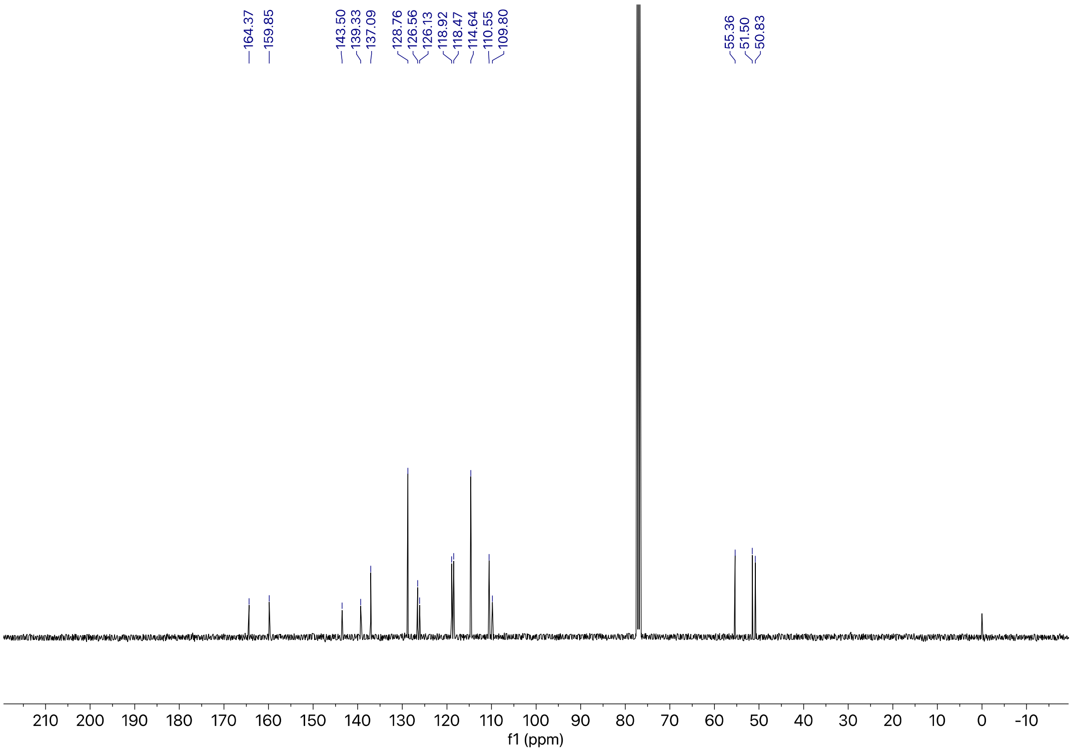

^{1}H NMR for Methyl 1-(4-chlorobenzyl)-5-nitro-1*H*-indole-3-carboxylate (**17l**)

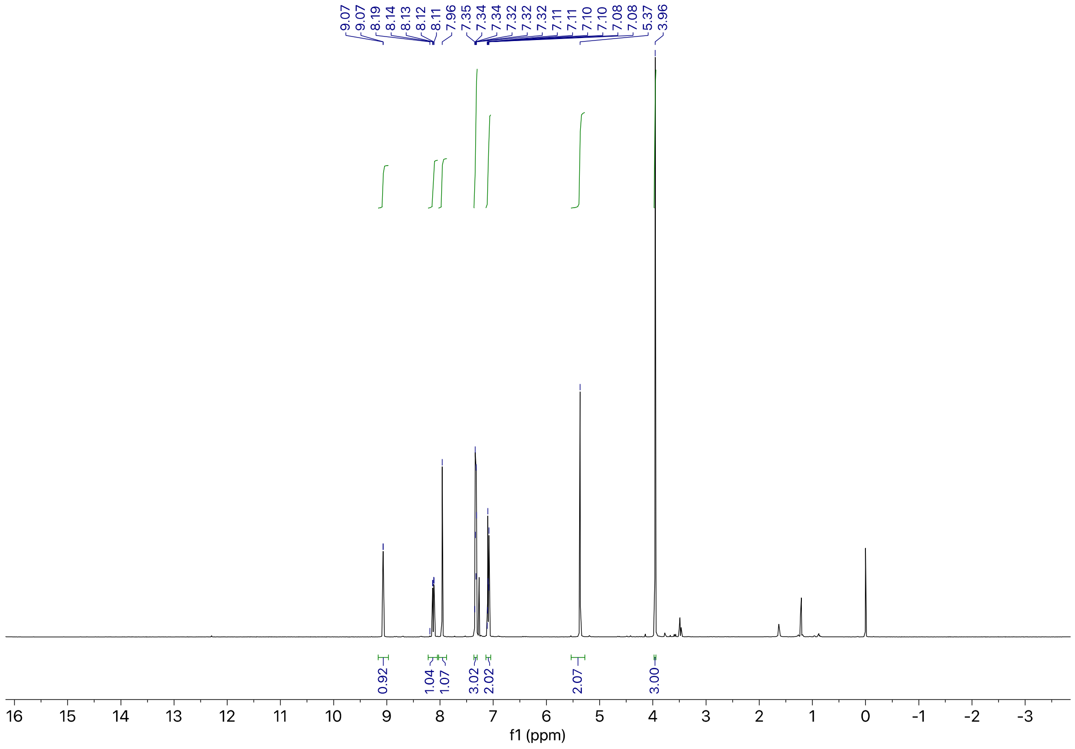

^{13}C NMR for Methyl 1-(4-chlorobenzyl)-5-nitro-1*H*-indole-3-carboxylate (**17l**)

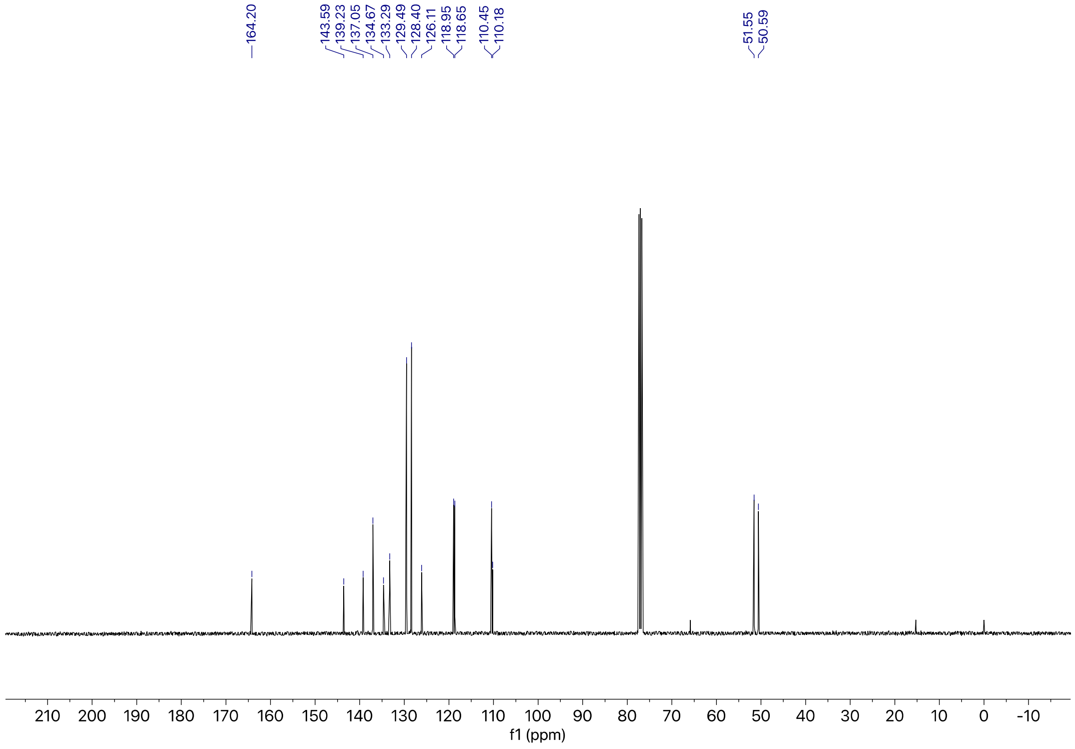

^{1}H NMR for methyl 5-nitro-1-((4-trifluoromethyl)benzyl)-1*H*-indole-3-carboxylate (**17m**)

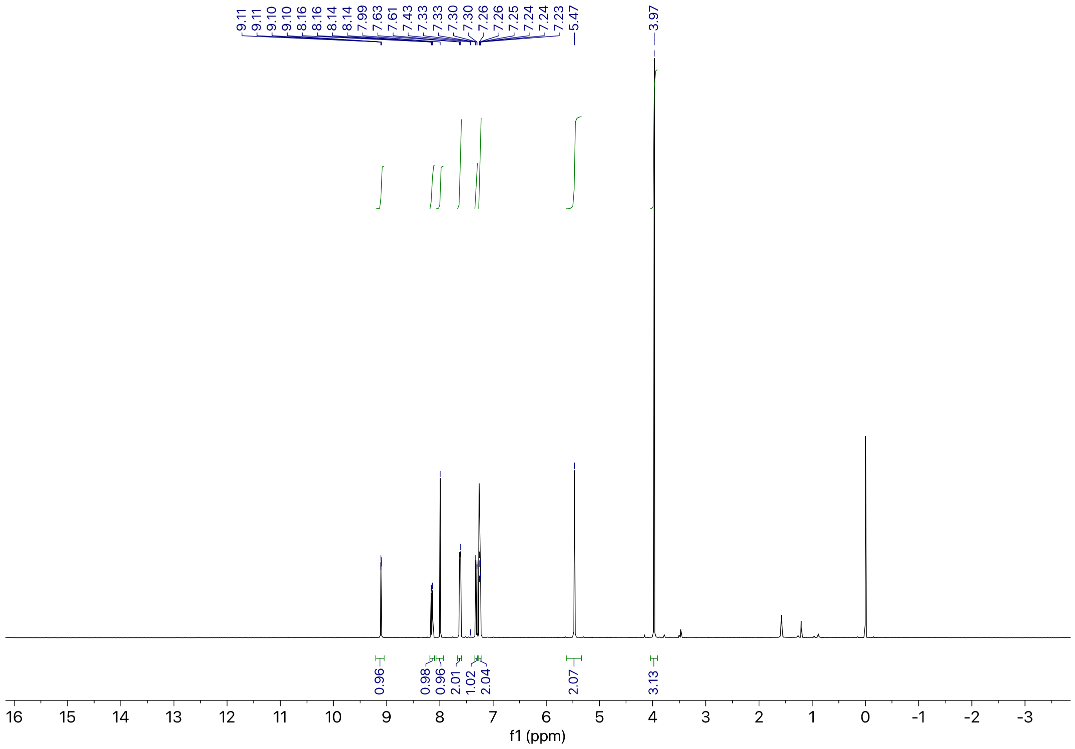

^{13}C NMR for methyl 5-nitro-1-((4-trifluoromethyl)benzyl)-1*H*-indole-3-carboxylate (**17m**)

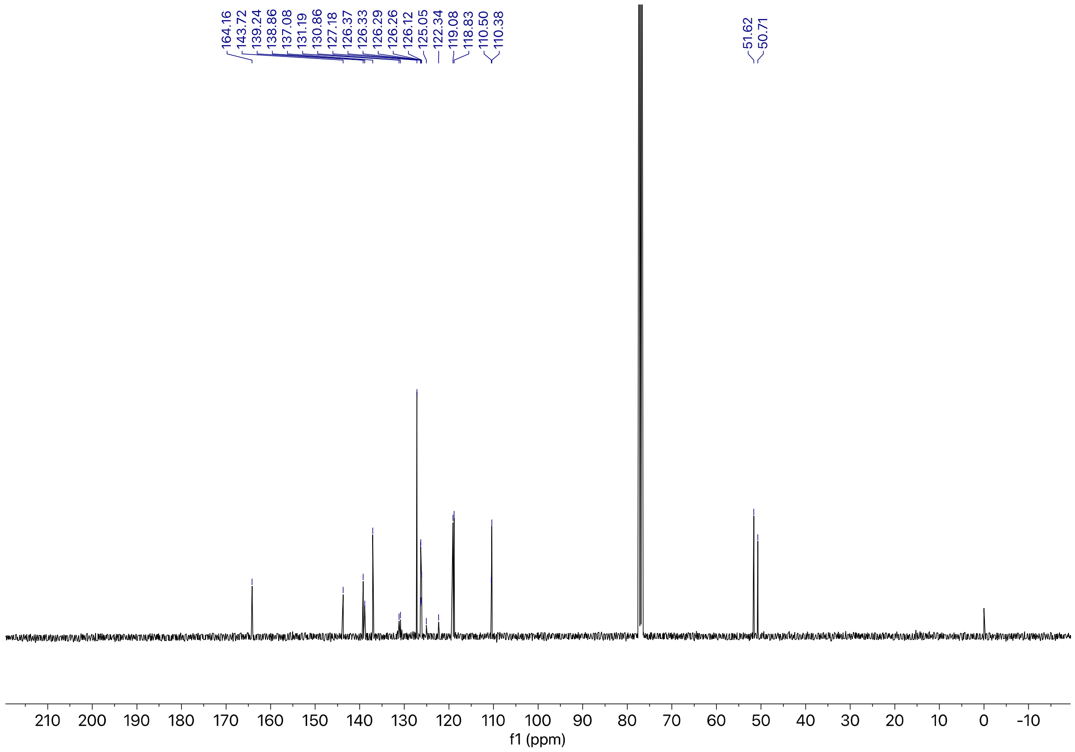

^{1}H NMR for methyl 5-nitro-1-(3-nitrobenzyl)-1*H*-indole-3-carboxylate (**17n**)

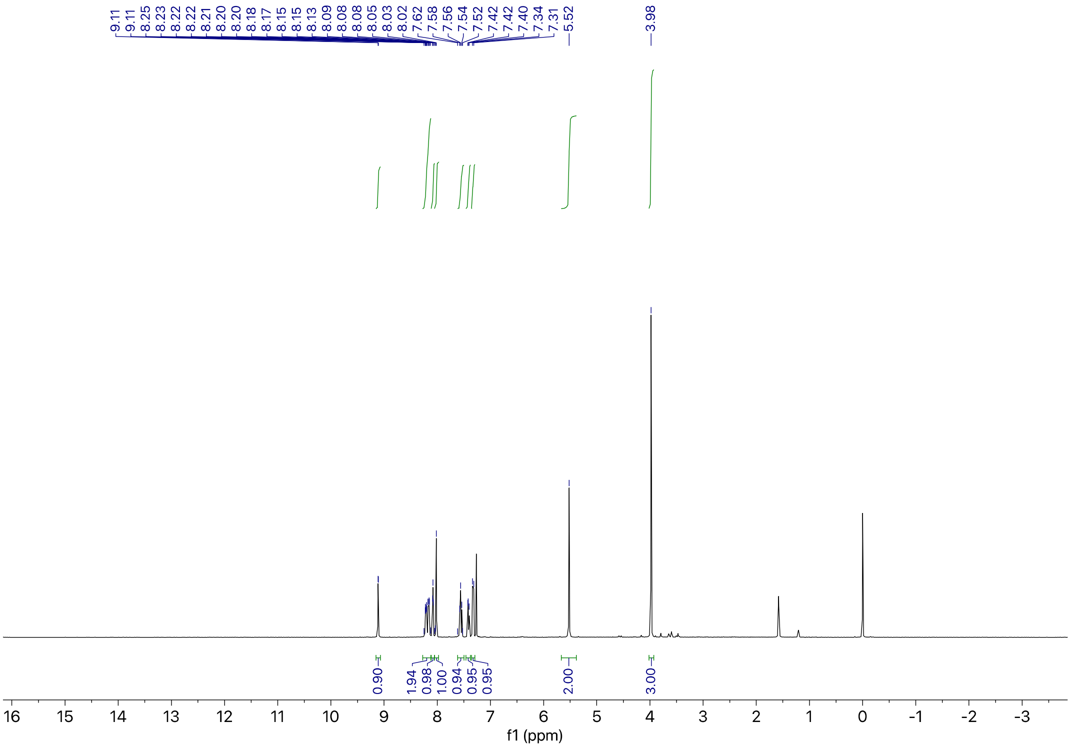

^{13}C NMR for methyl 5-nitro-1-(3-nitrobenzyl)-1*H*-indole-3-carboxylate (**17n**)

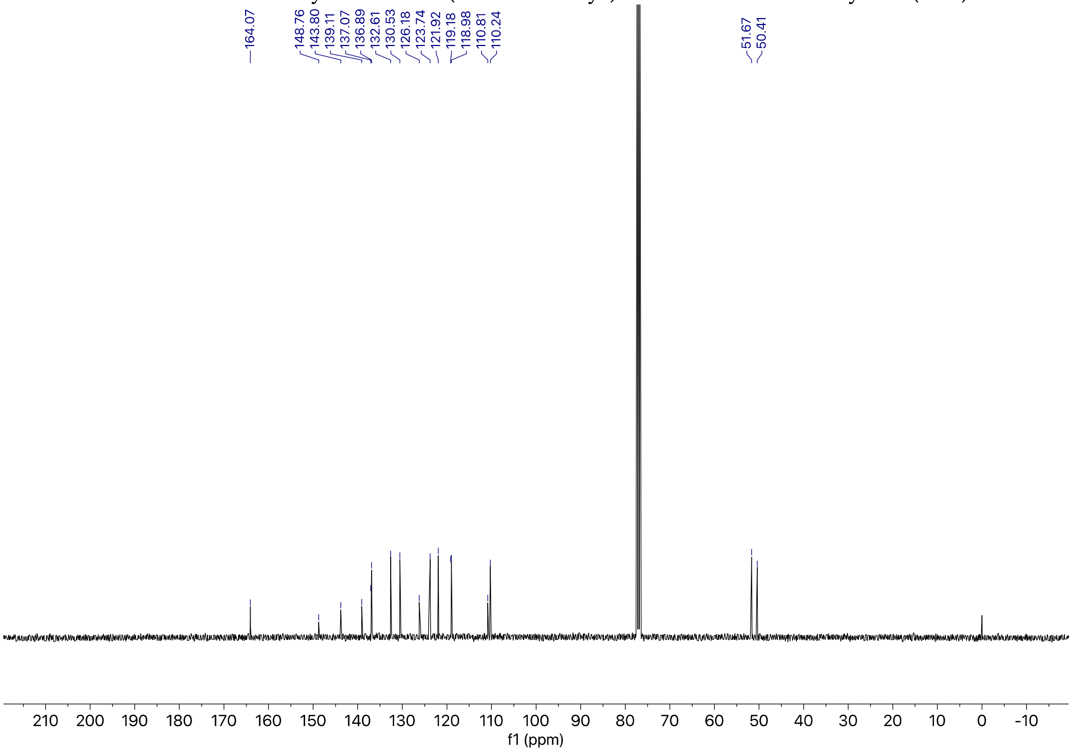

^{1}H NMR for methyl 1-amino-5-nitro-1*H*-indole-3-carboxylate (**17o**)

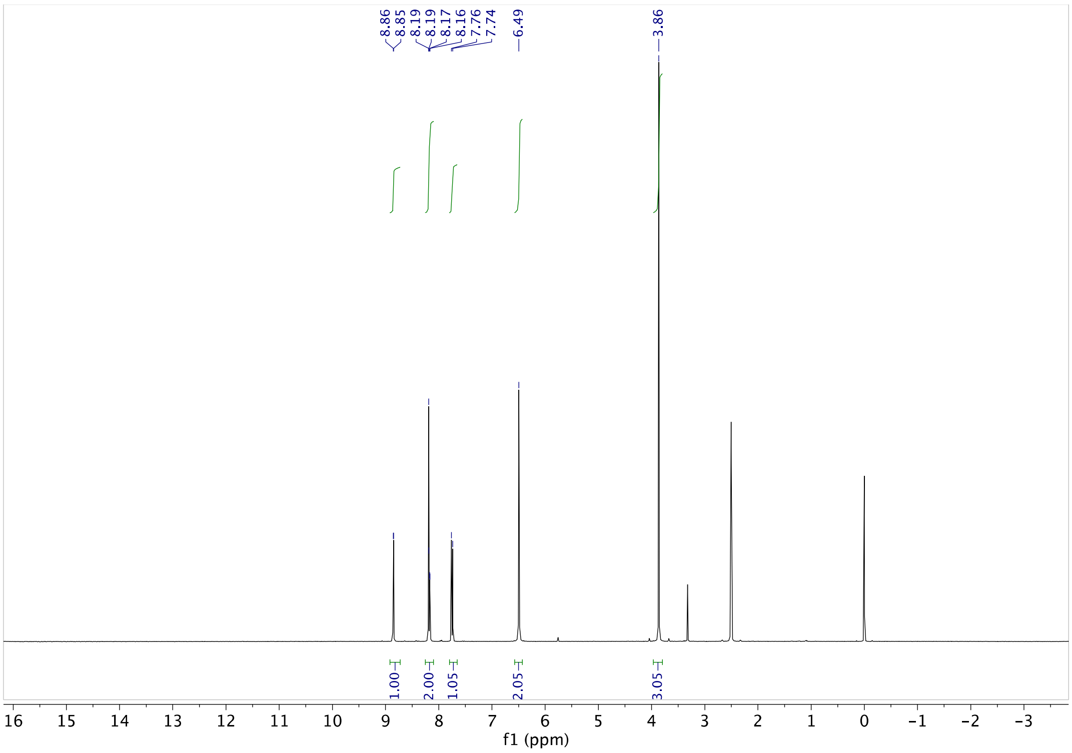

^{13}C NMR for methyl 1-amino-5-nitro-1*H*-indole-3-carboxylate (**17o**)

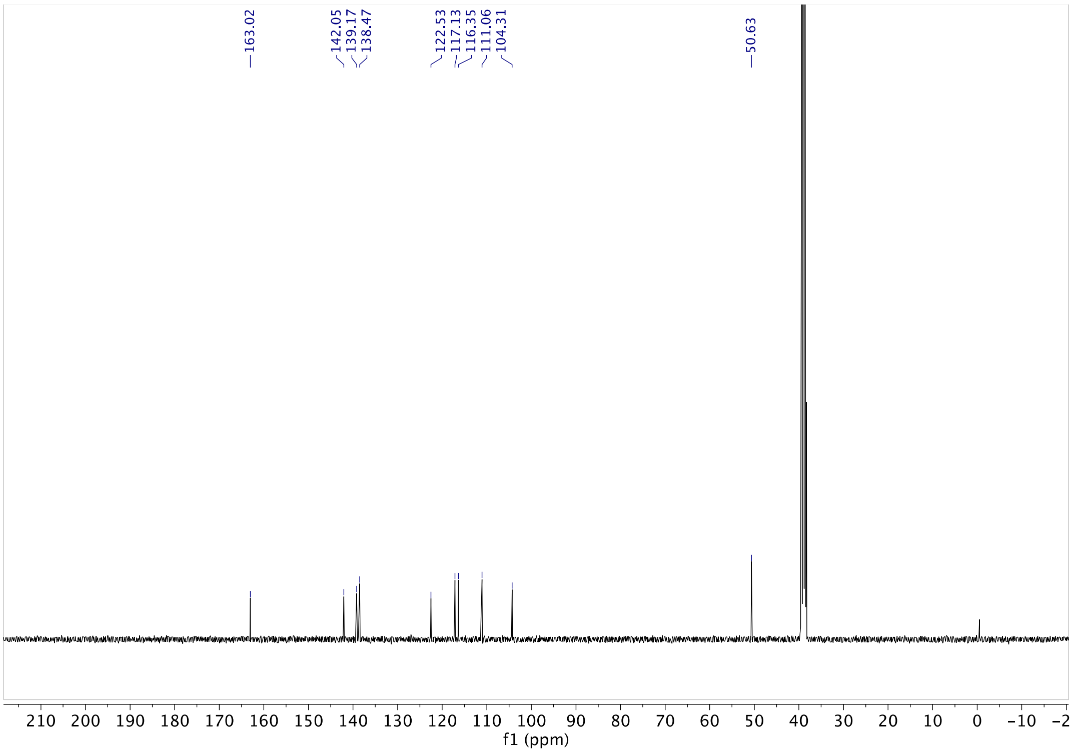

^{1}H NMR for methyl 5-cyano-1-hexyl-1*H*-indole-3-carboxylate (**18a**)

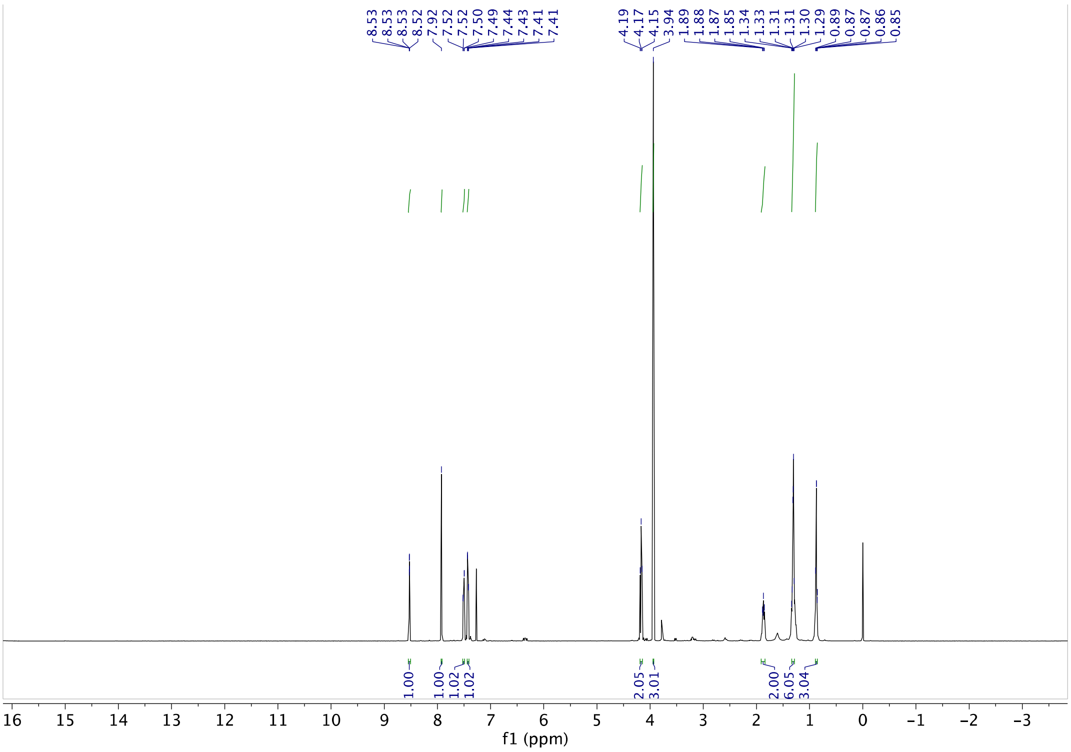

^{13}C NMR for methyl 5-cyano-1-hexyl-1*H*-indole-3-carboxylate (**18a**)

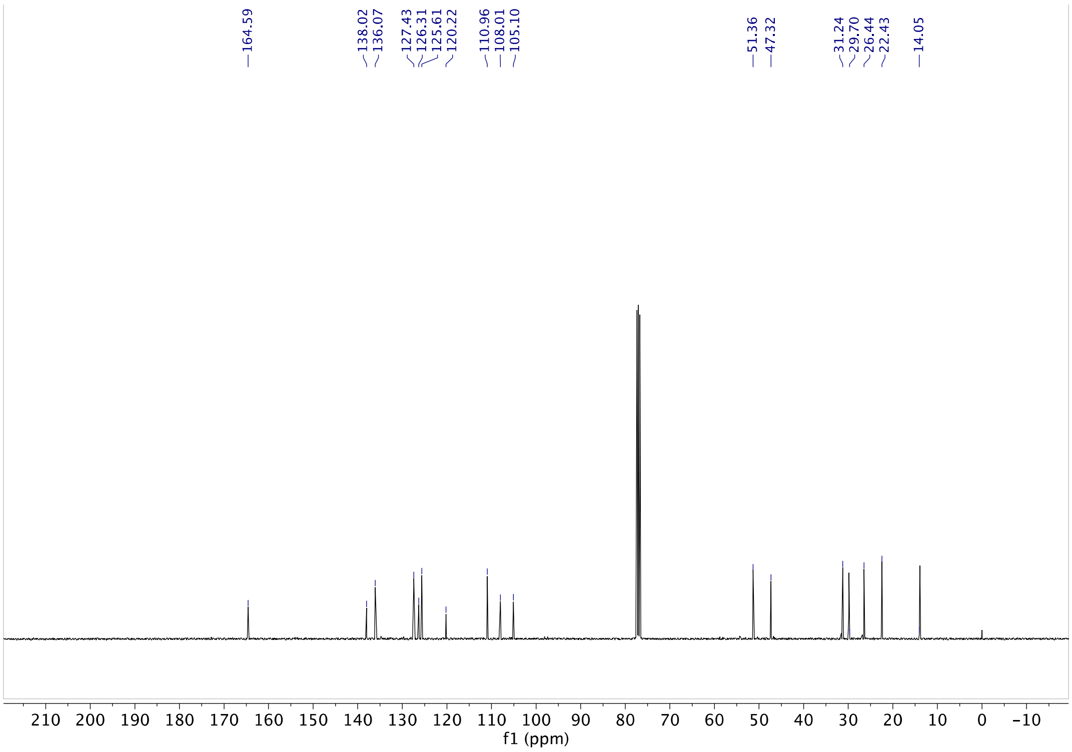

^{1}H NMR for methyl 5-cyano-1-isobutyl-1*H*-indole-3-carboxylate (**18b**)

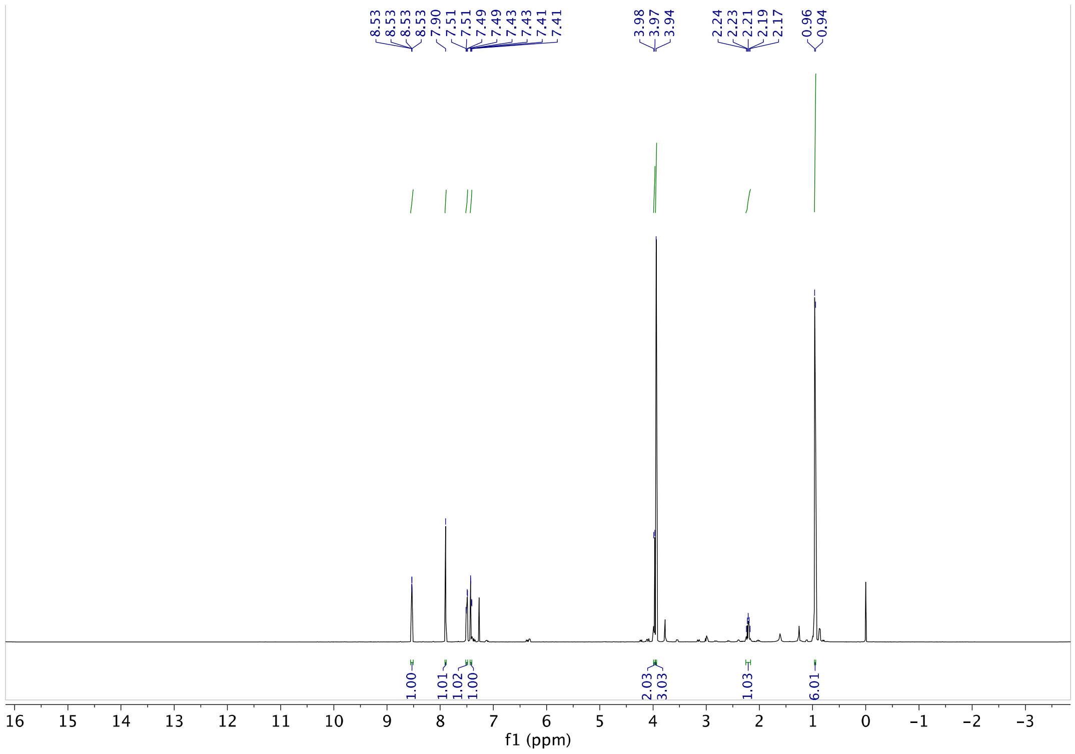

^{13}C NMR for methyl 5-cyano-1-isobutyl-1*H*-indole-3-carboxylate (**18b**)

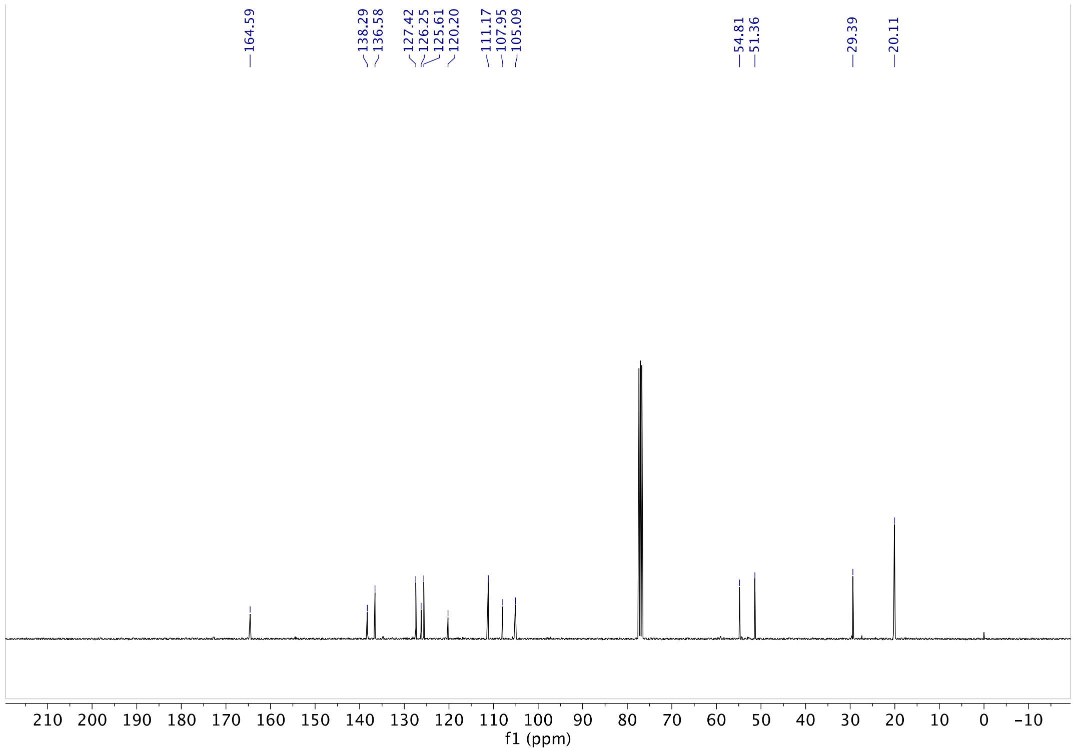

¹H NMR for methyl 5-cyano-1-cyclopropyl-1*H*-indole-3-carboxylate (**18d**)

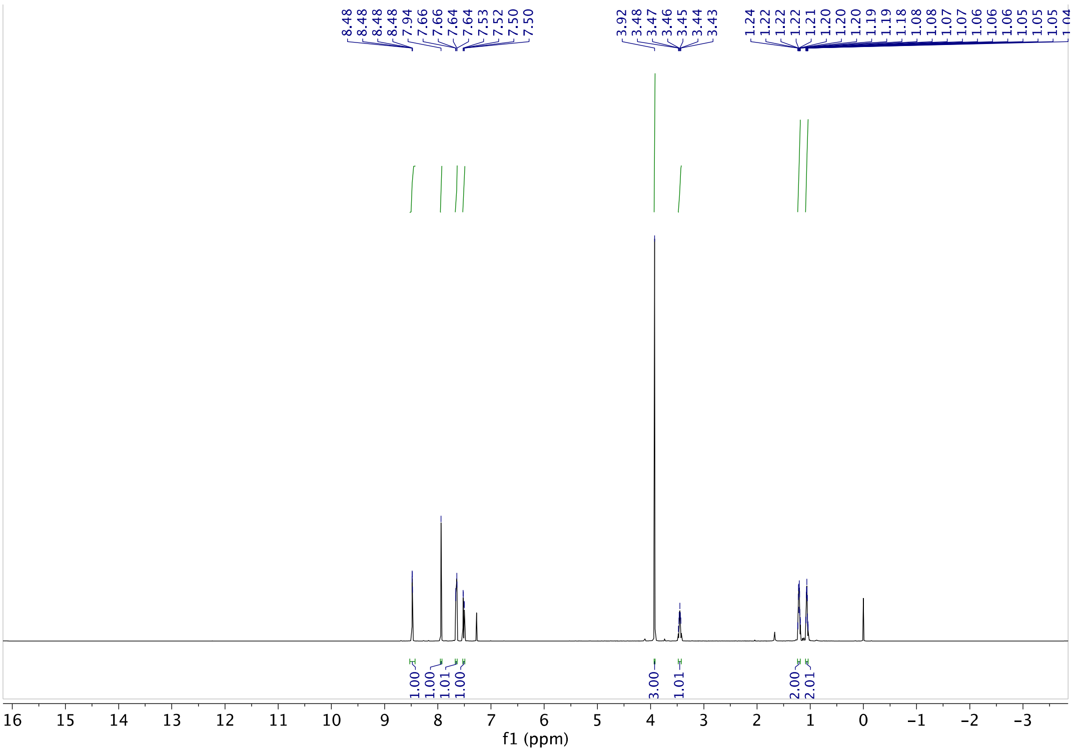

¹³C NMR for methyl 5-cyano-1-cyclopropyl-1*H*-indole-3-carboxylate (**18d**)

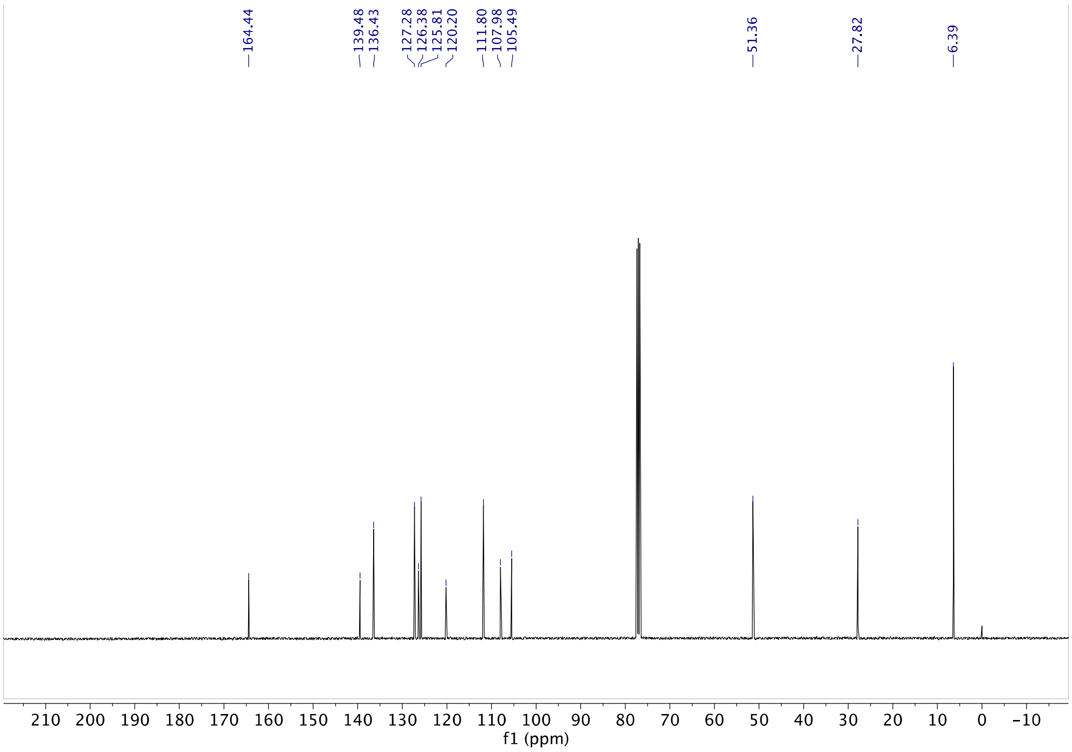

^{1}H NMR for methyl 5-cyano-1-cyclohexyl-1*H*-indole-3-carboxylate (**18e**)

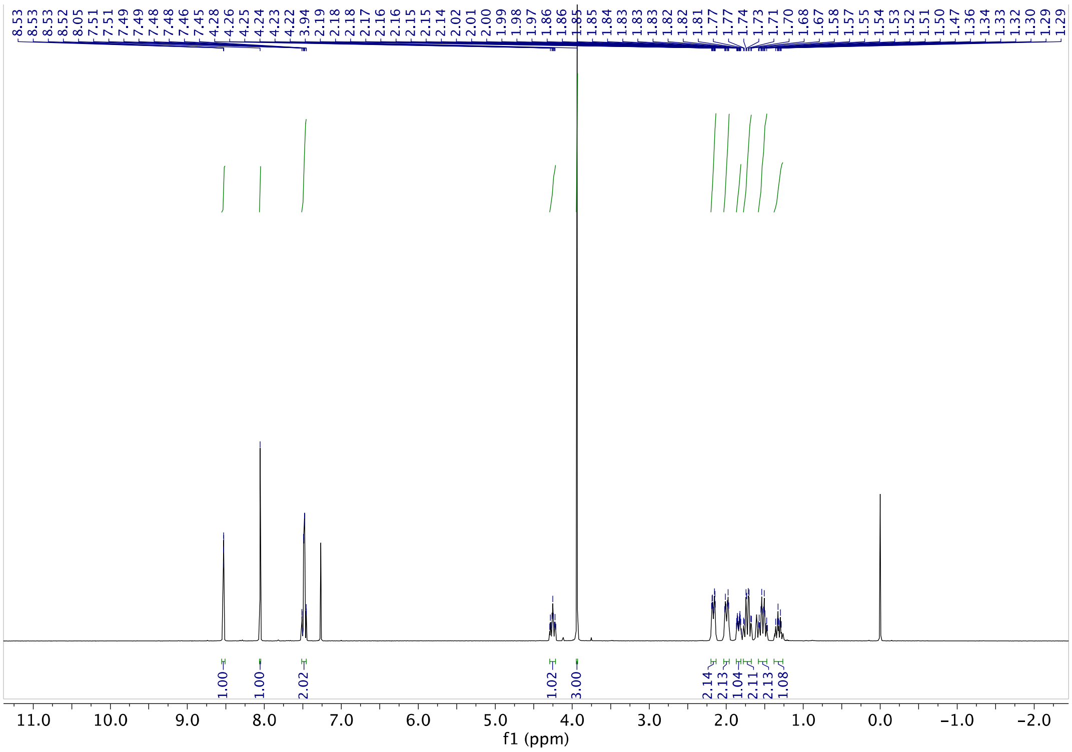

^{13}C NMR for methyl 5-cyano-1-cyclohexyl-1*H*-indole-3-carboxylate (**18e**)

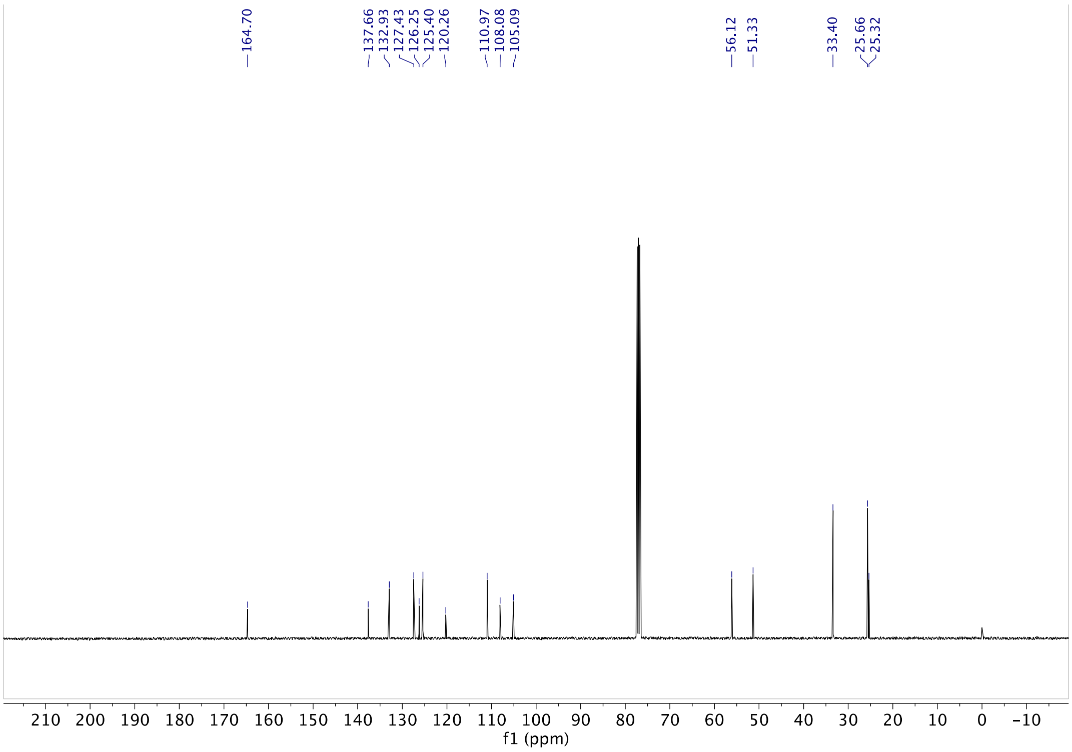

^{1}H NMR for methyl 5-cyano-1-phenethyl-1*H*-indole-3-carboxylate (**18g**)

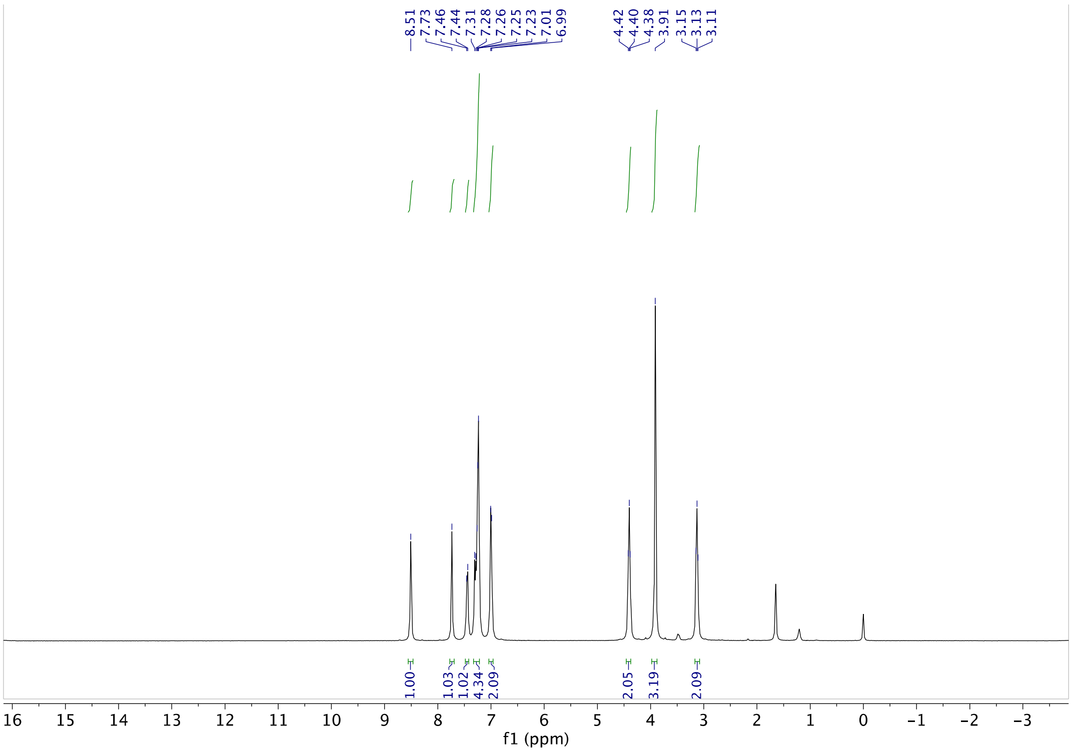

^{13}C NMR for methyl 5-cyano-1-phenethyl-1*H*-indole-3-carboxylate (**18g**)

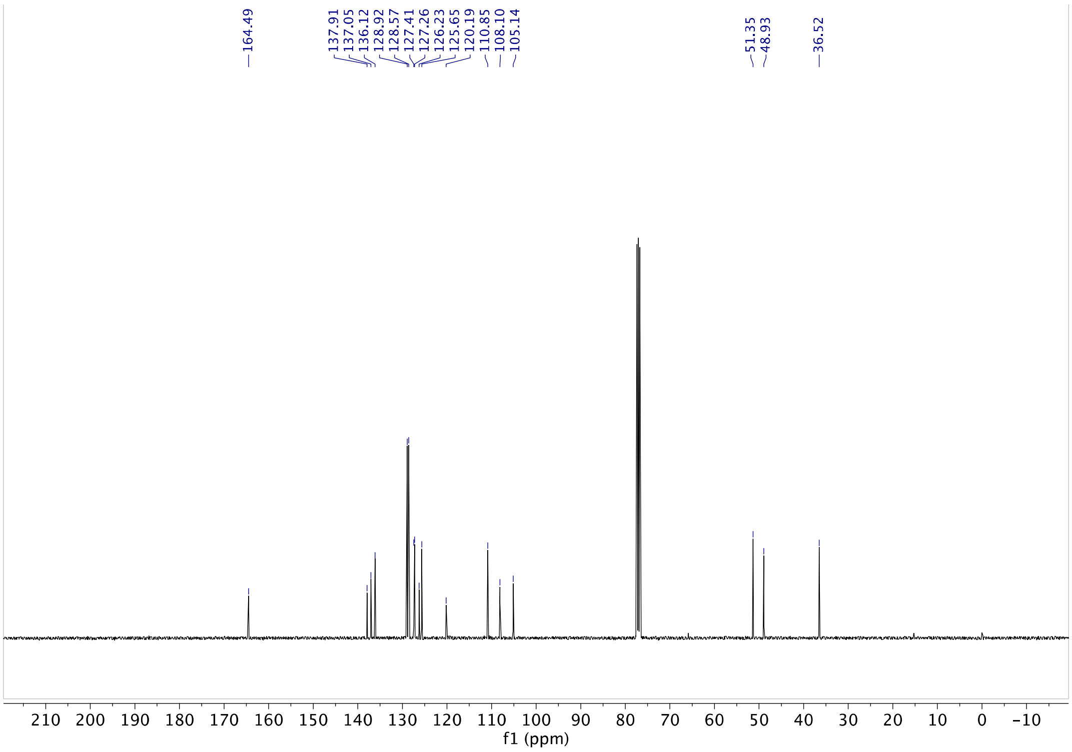

^{1}H NMR for methyl 1-benzyl-5-cyano-1*H*-indole-3-carboxylate (**18h**)

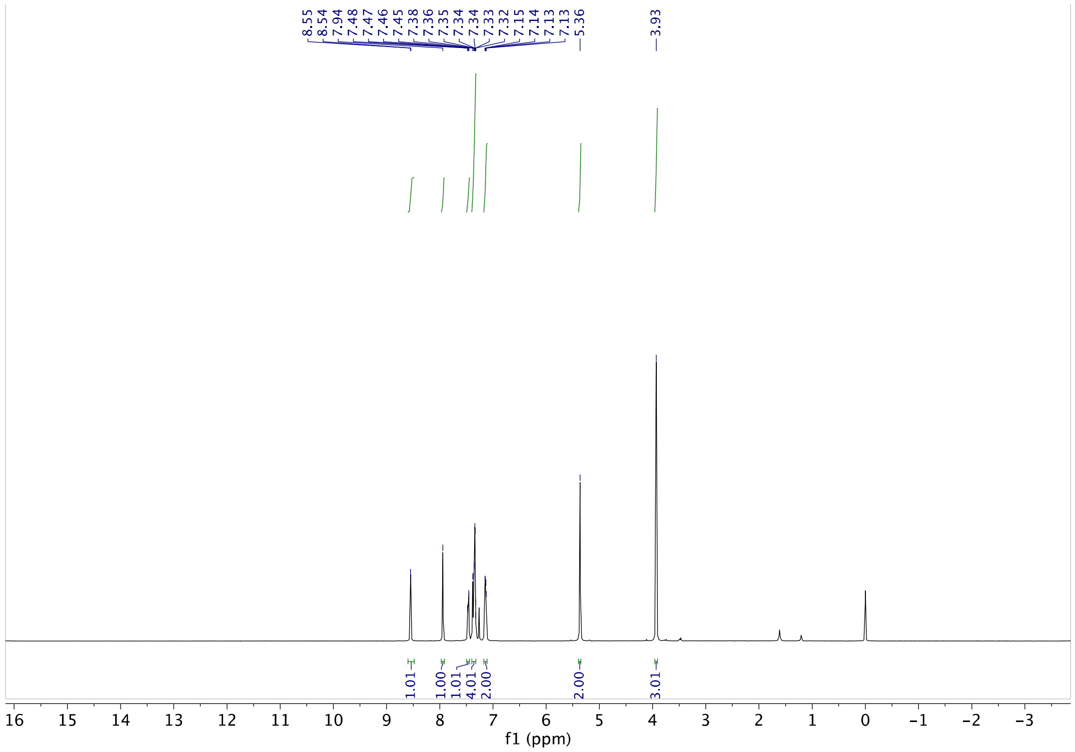

^{13}C NMR for methyl 1-benzyl-5-cyano-1*H*-indole-3-carboxylate (**18h**)

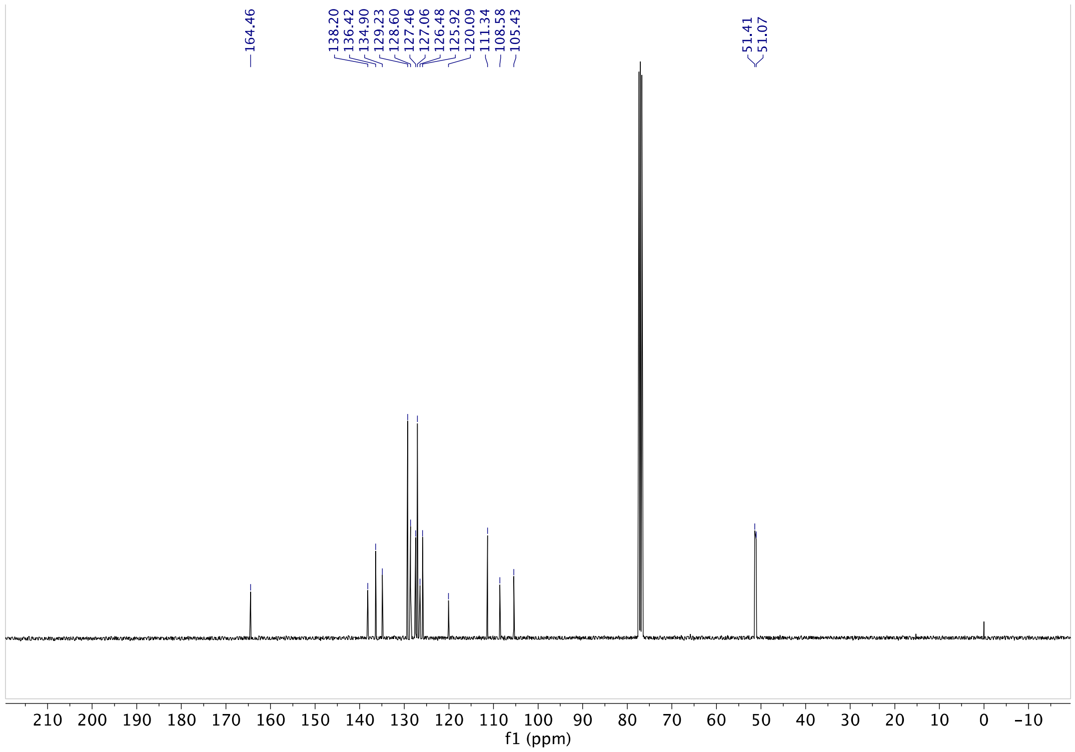

^{1}H NMR for methyl 5-cyano-1-(4-methylbenzyl)-1H-indole-3-carboxylate (**18i**)

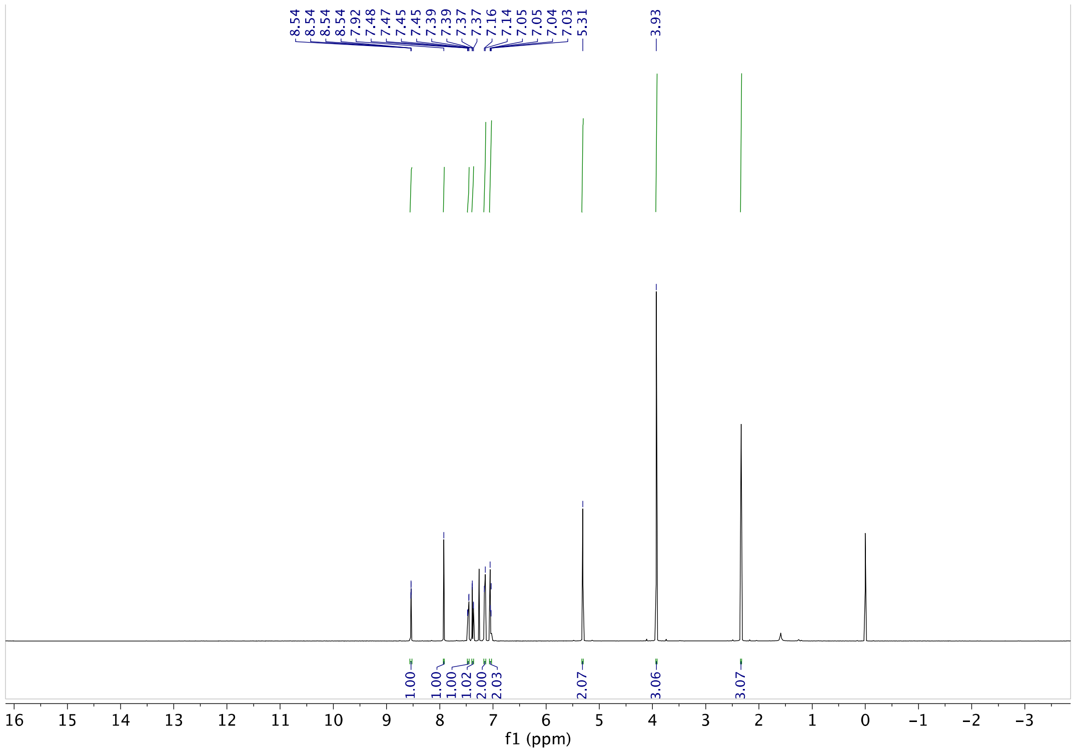

^{13}C NMR for methyl 5-cyano-1-(4-methylbenzyl)-1H-indole-3-carboxylate (**18i**)

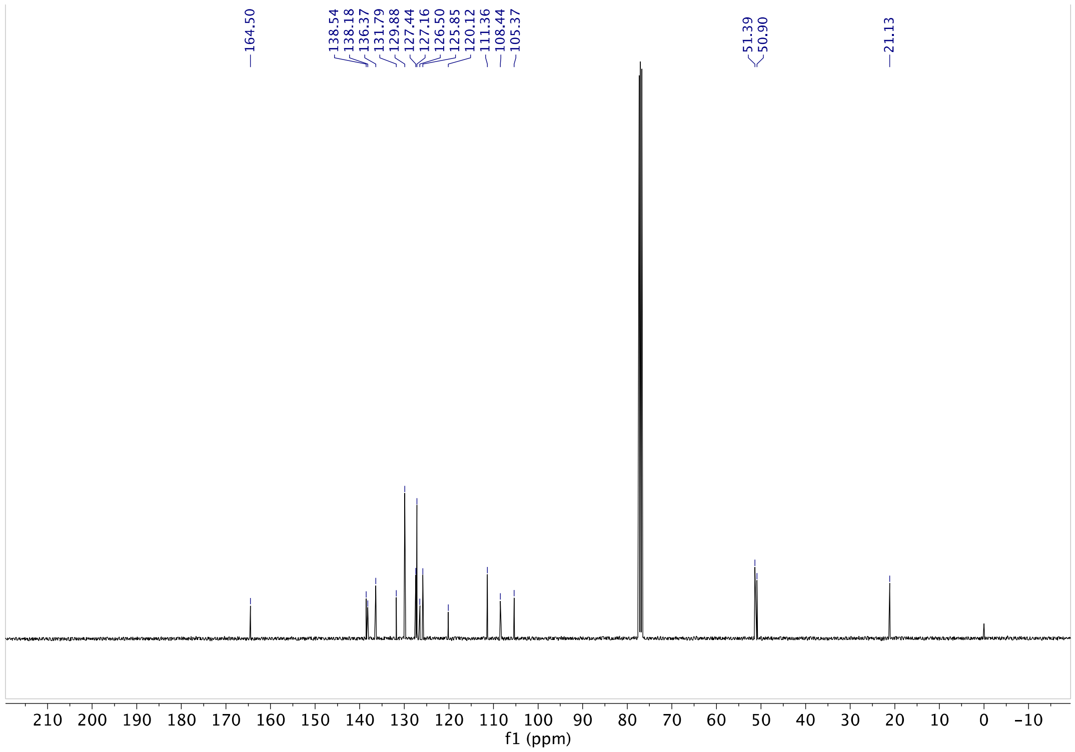

^{1}H NMR for methyl 5-cyano-1-(3-methoxyphenyl)-1*H*-indole-3-carboxylate (**18j**)

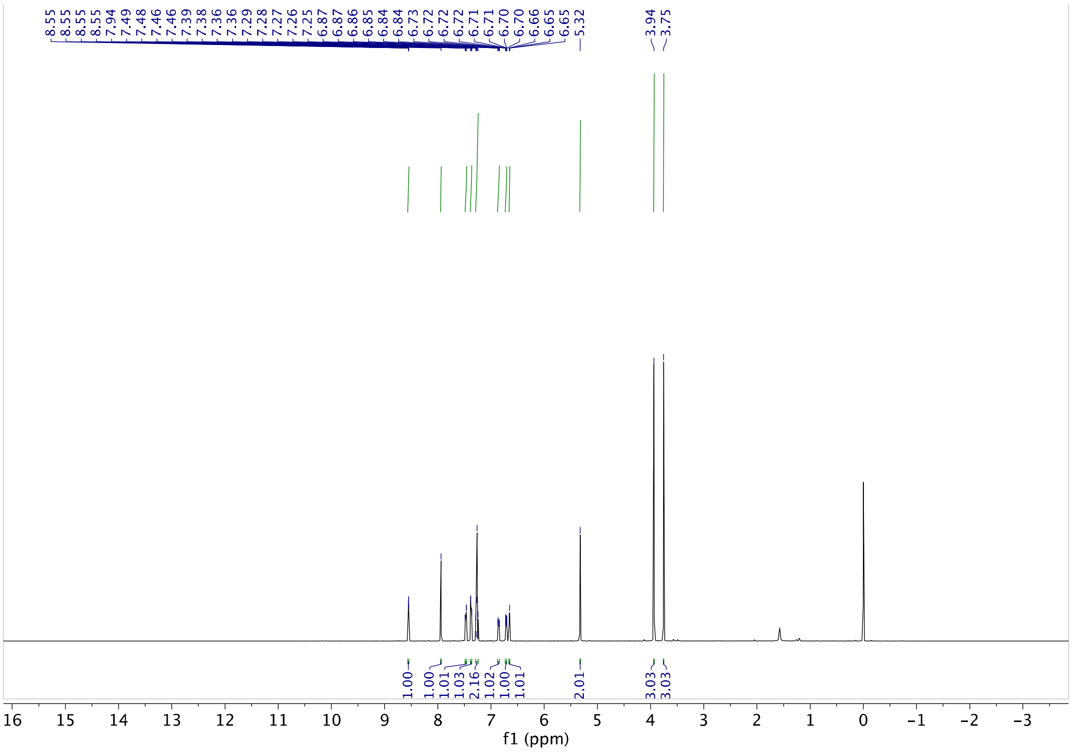

^{13}C NMR for methyl 5-cyano-1-(3-methoxyphenyl)-1*H*-indole-3-carboxylate (**18j**)

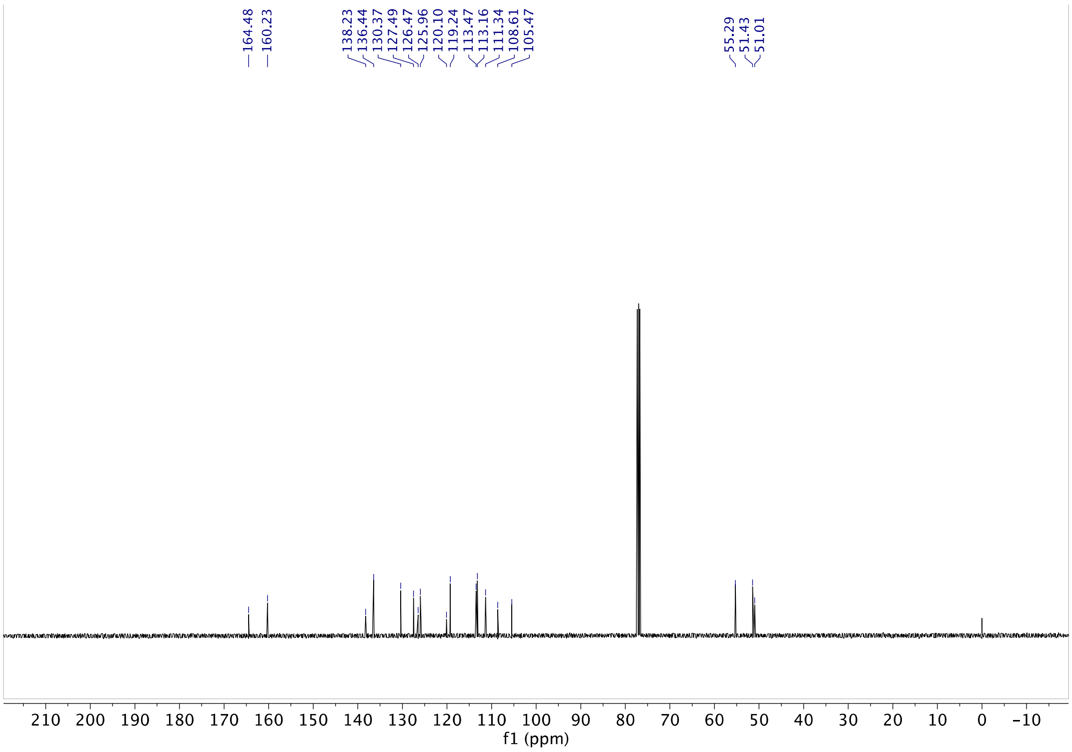

^{1}H NMR for methyl 5-cyano-1-(4-methoxyphenyl)-1*H*-indole-3-carboxylate (**18k**)

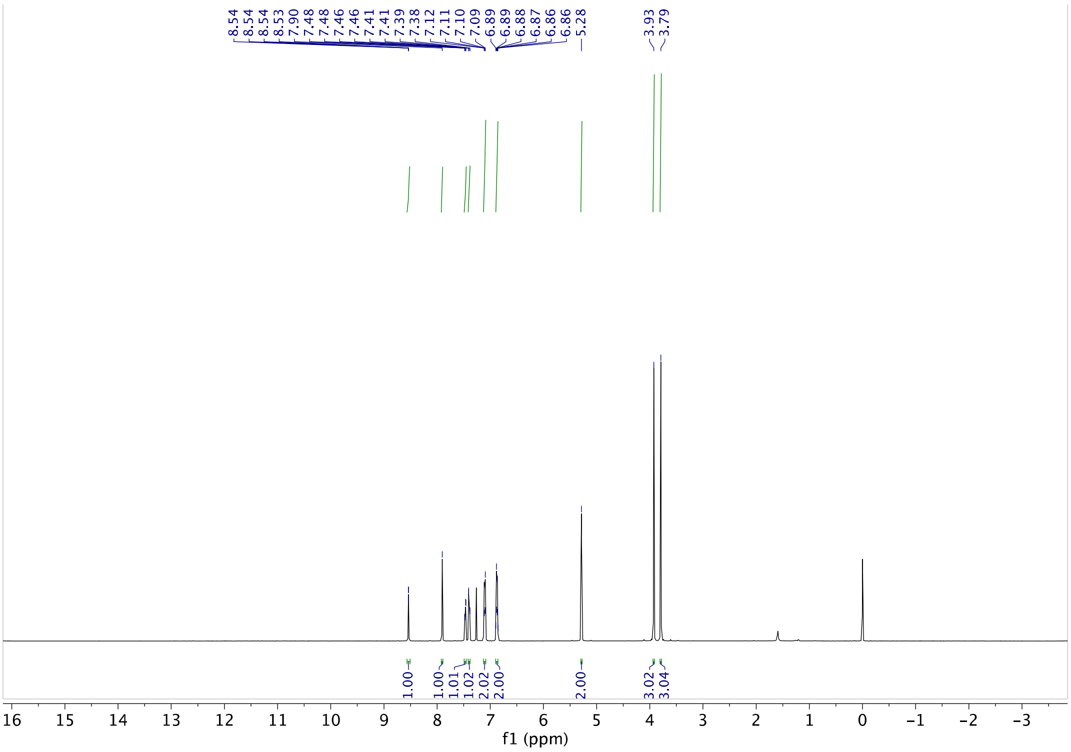

^{13}C NMR for methyl 5-cyano-1-(4-methoxyphenyl)-1*H*-indole-3-carboxylate (**18k**)

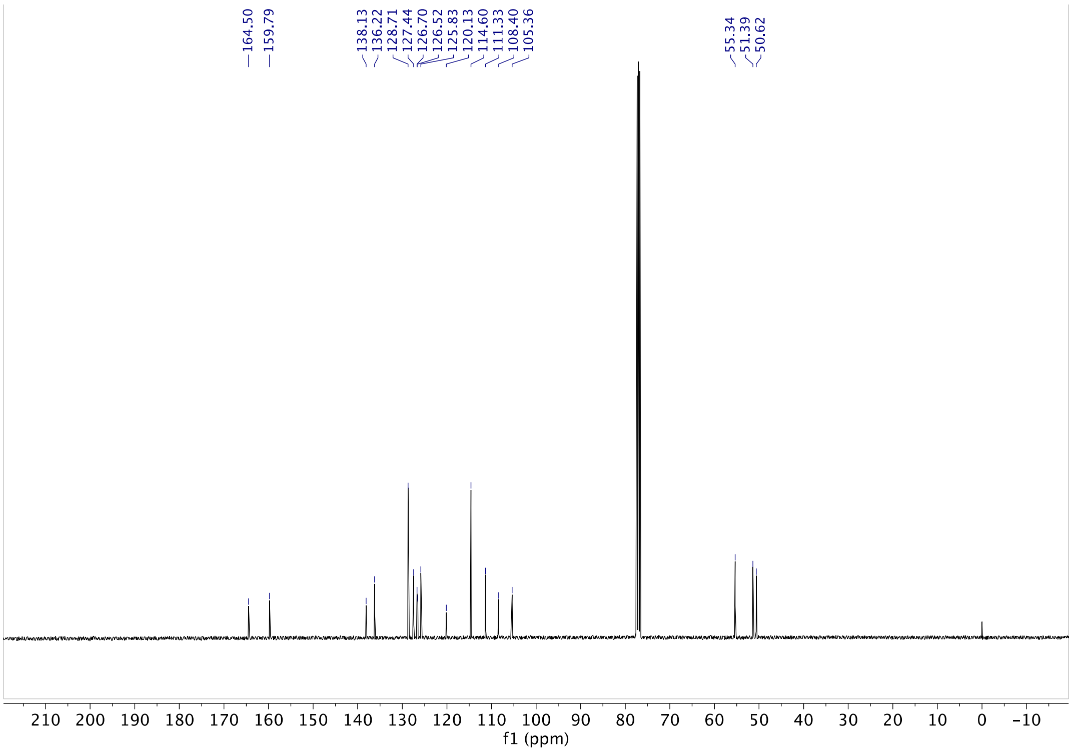

^{1}H NMR for methyl 1-(4-chlorophenyl)-5-cyano-1*H*-indole-3-carboxylate (**18l**)

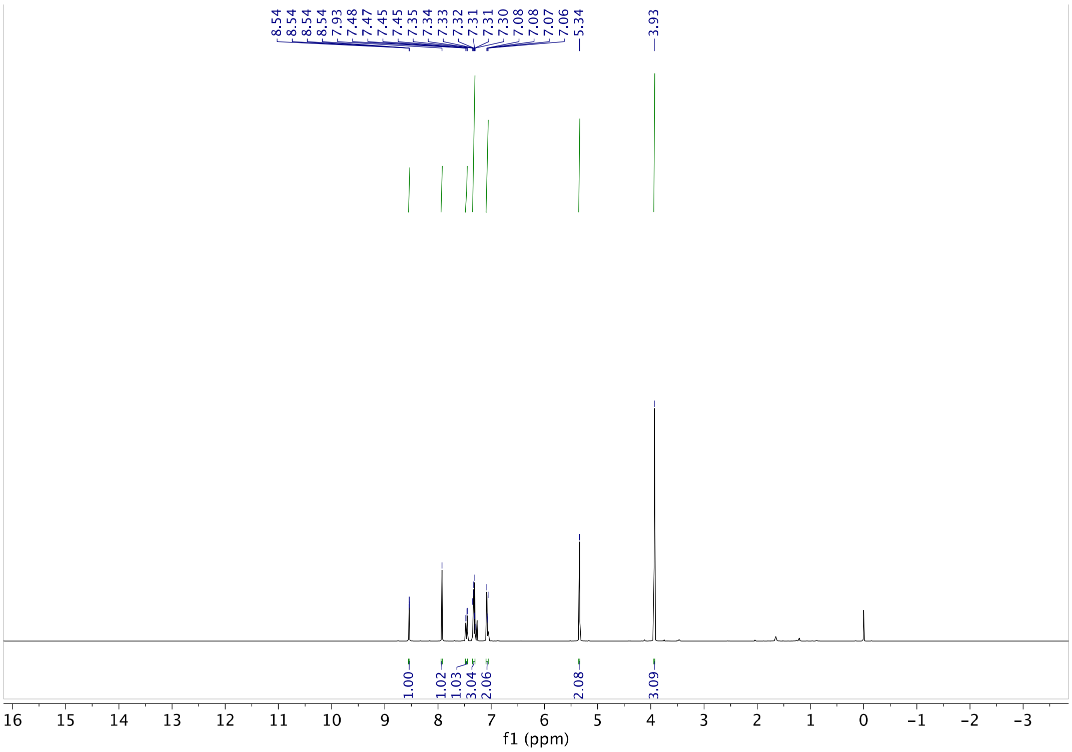

^{13}C NMR for methyl 1-(4-chlorophenyl)-5-cyano-1*H*-indole-3-carboxylate (**18l**)

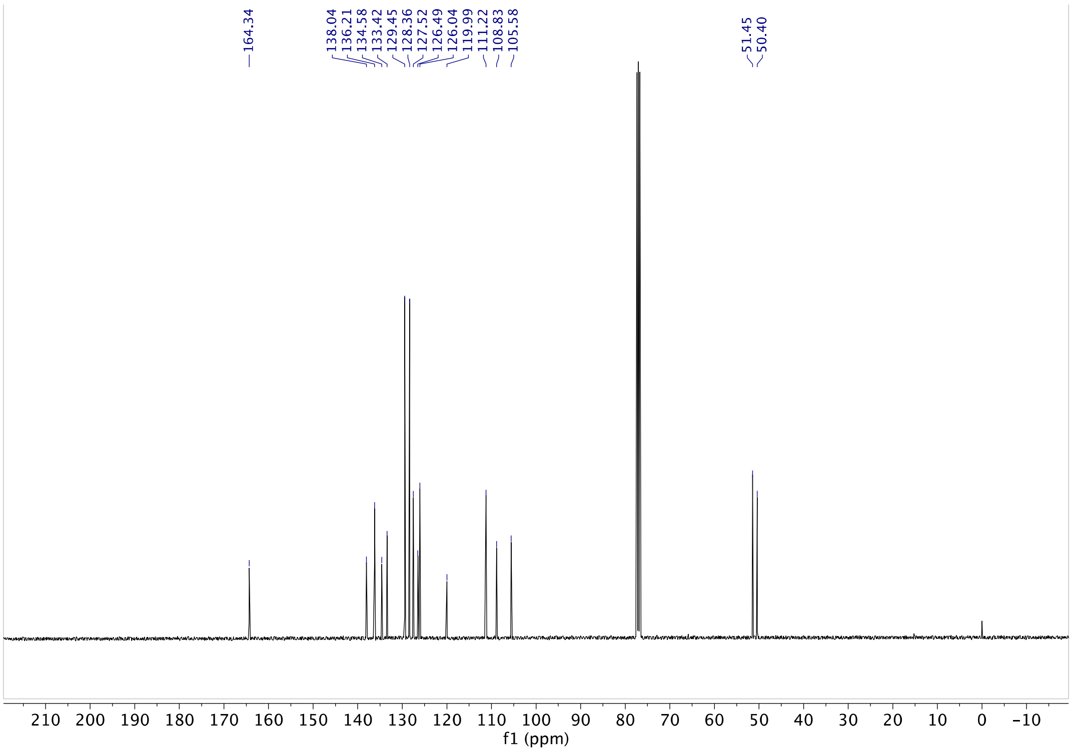

^{1}H NMR for methyl 5-cyano-1-(3-nitrobenzyl)-1*H*-indole-3-carboxylate (**18m**)

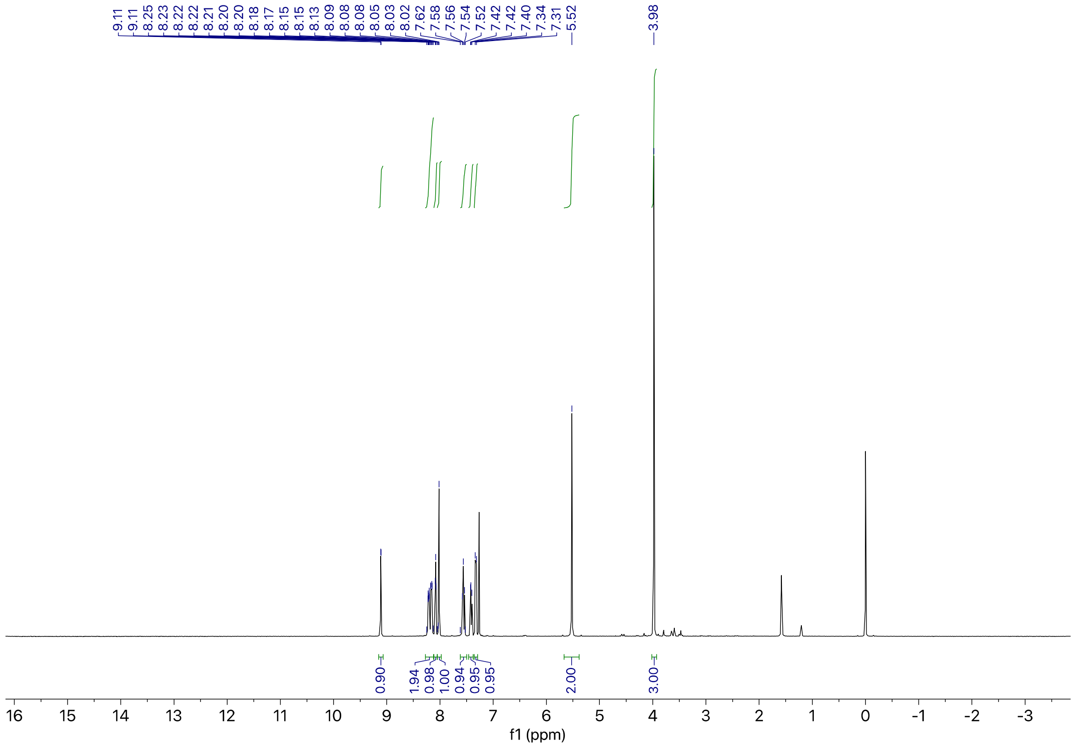

^{13}C NMR for methyl 5-cyano-1-(3-nitrobenzyl)-1*H*-indole-3-carboxylate (**18m**)

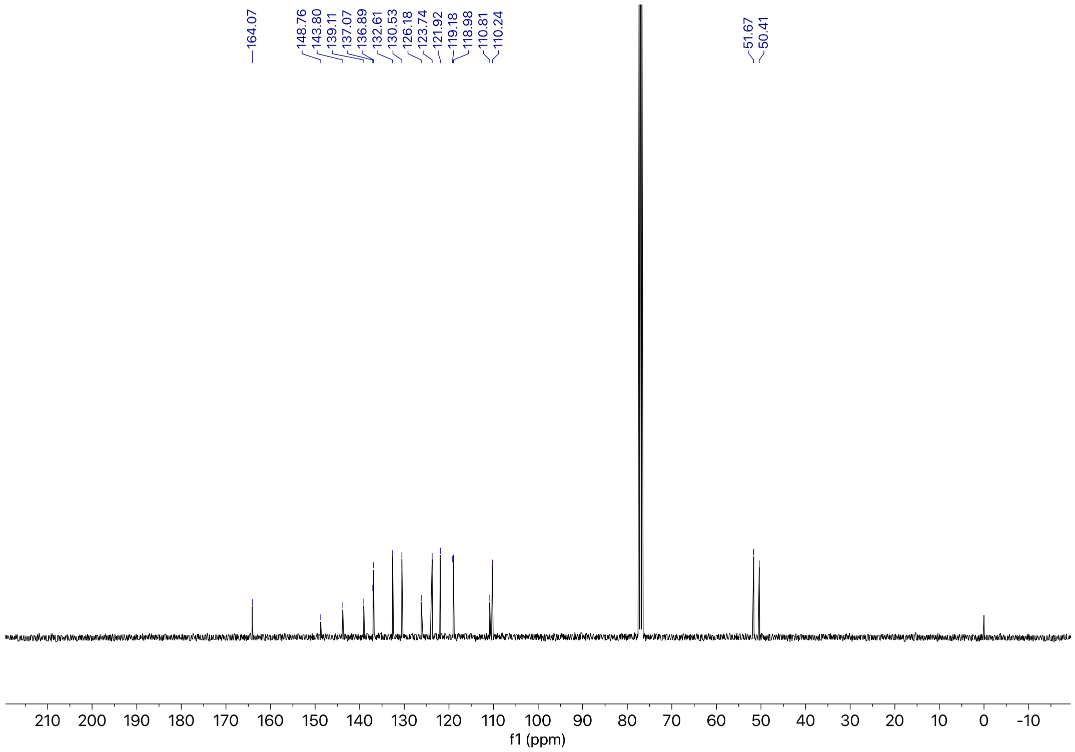

^{1}H NMR for dimethyl 1-hexyl-1*H*-indole-3,5-dicarboxylate (**19a**)

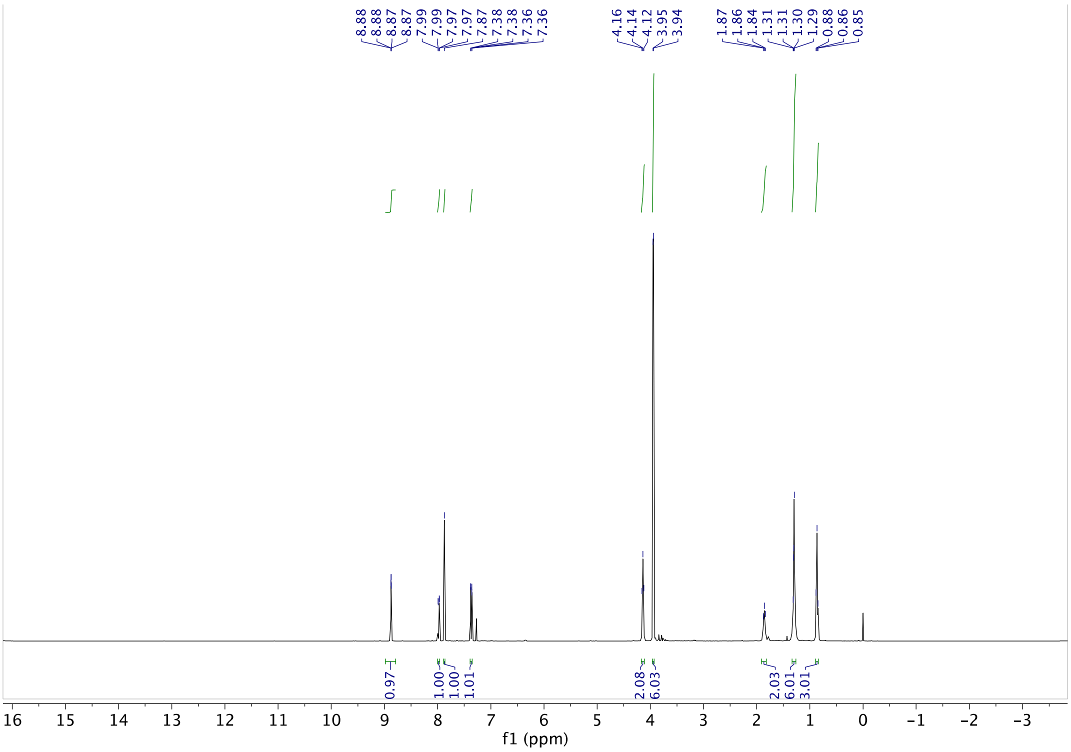

^{13}C NMR for dimethyl 1-hexyl-1*H*-indole-3,5-dicarboxylate (**19a**)

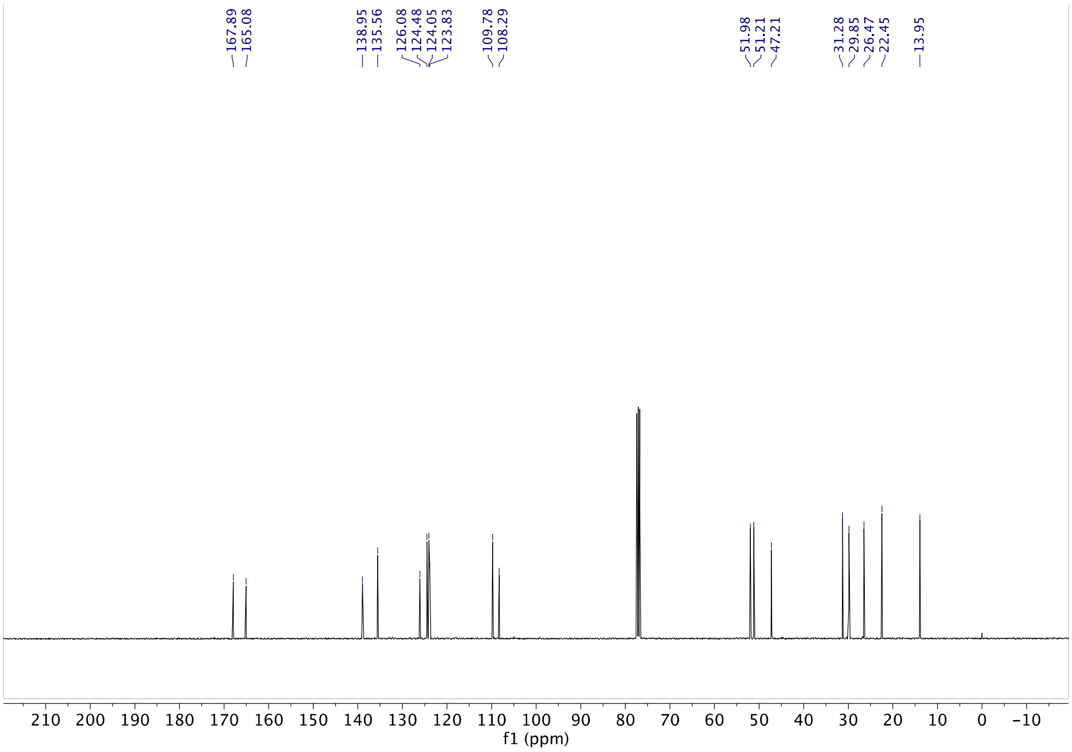

^{1}H NMR for dimethyl 1-isobutyl-1*H*-indole-3,5-dicarboxylate (**19b**)

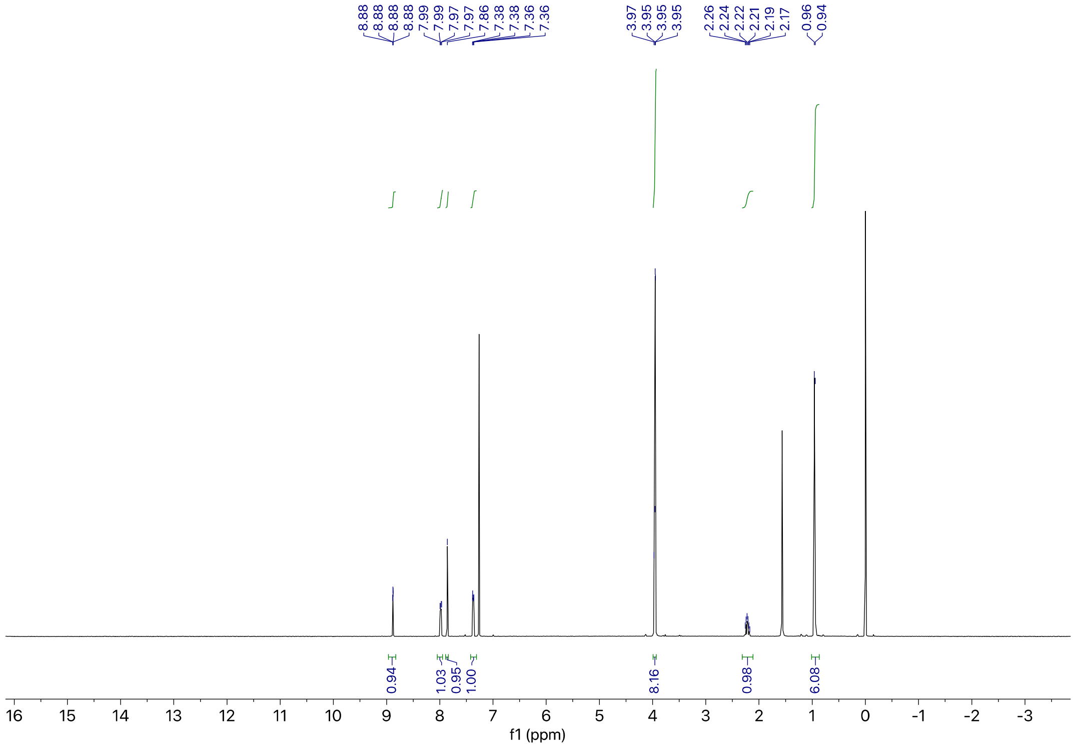

^{13}C NMR for dimethyl 1-isobutyl-1*H*-indole-3,5-dicarboxylate (**19b**)

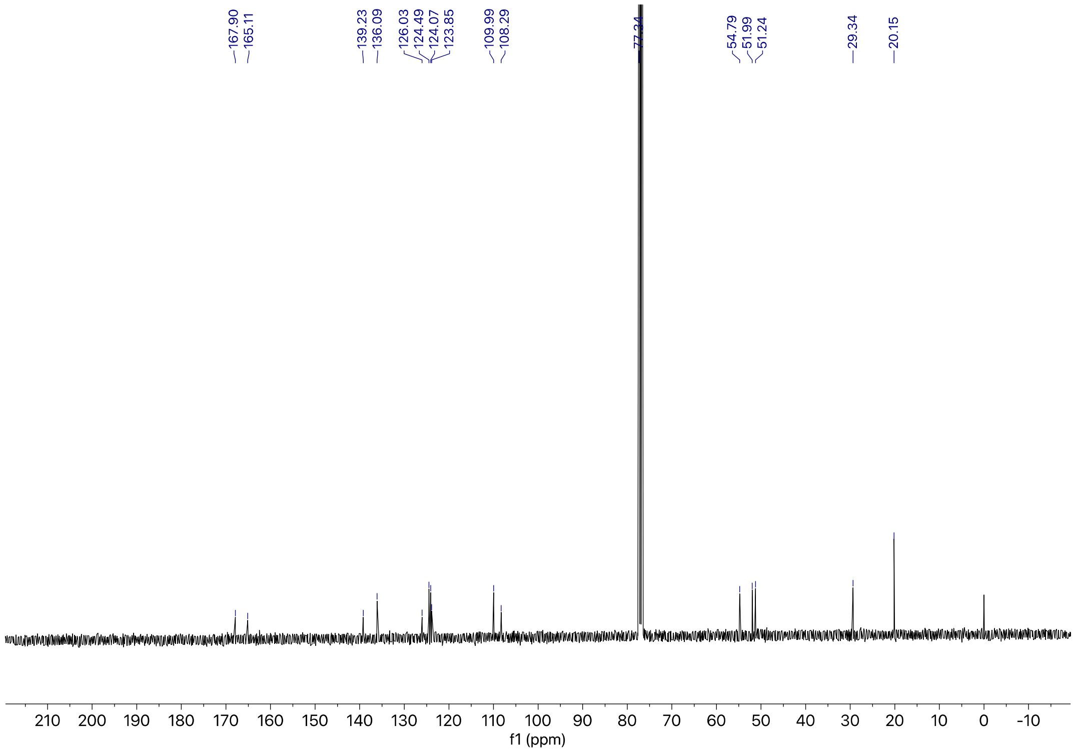

^{1}H NMR for dimethyl 1-allyl-1*H*-indole-3,5-dicarboxylate (**19c**)

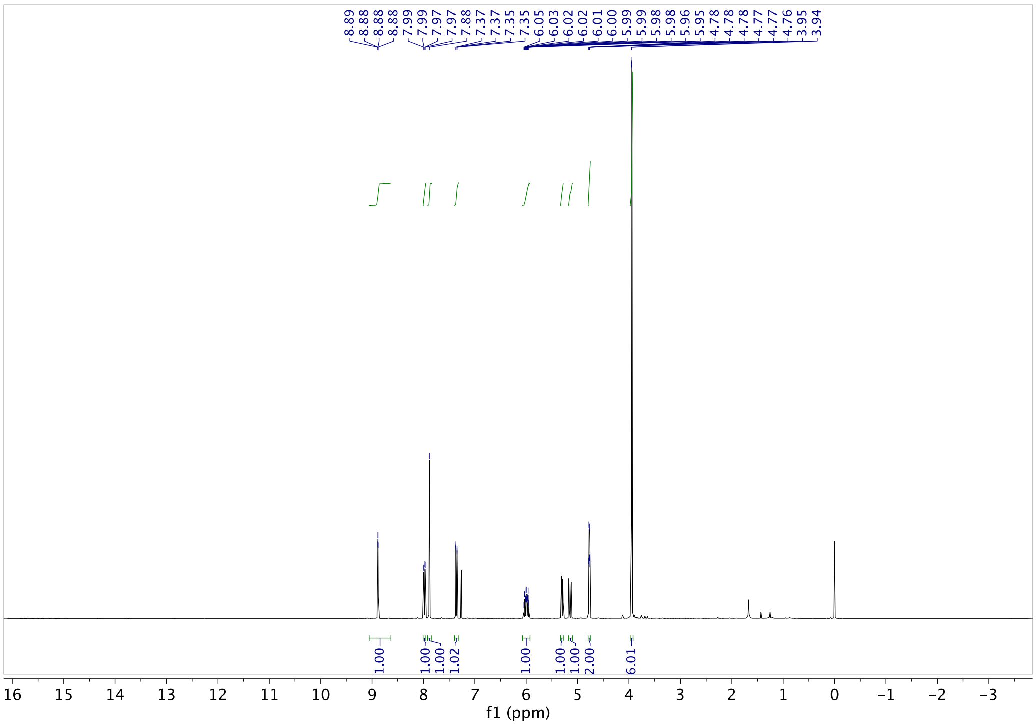

^{13}C NMR for dimethyl 1-allyl-1*H*-indole-3,5-dicarboxylate (**19c**)

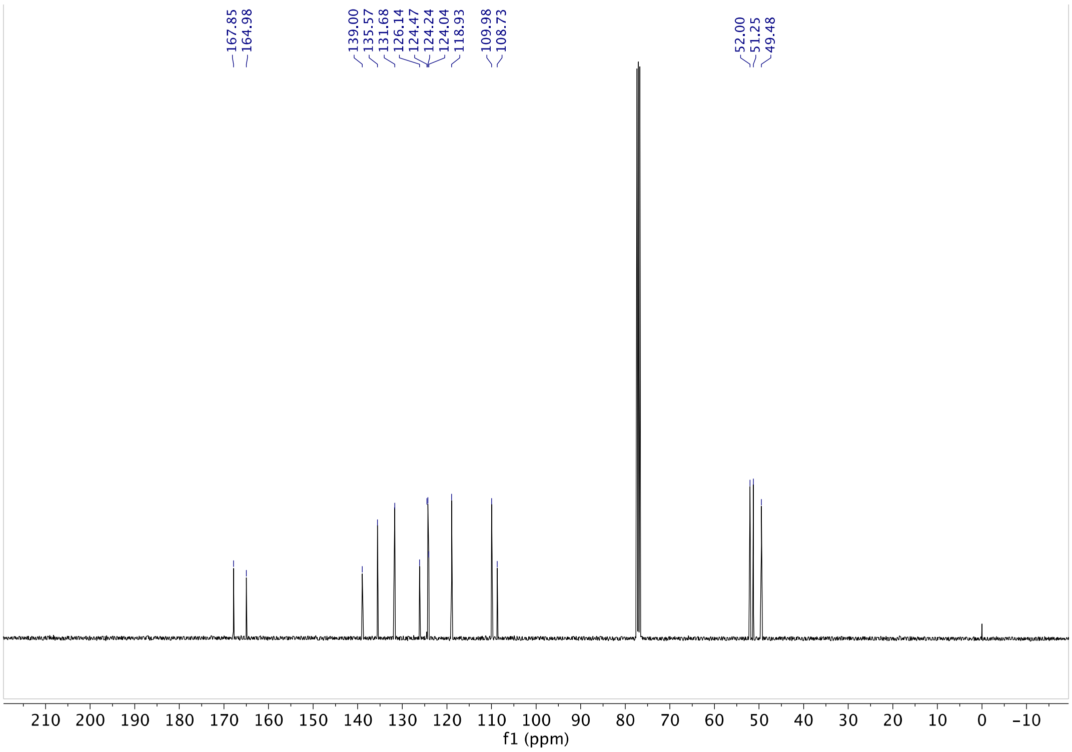

^{1}H NMR for dimethyl 1-cyclopropyl-1*H*-indole-3,5-dicarboxylate (**19d**)

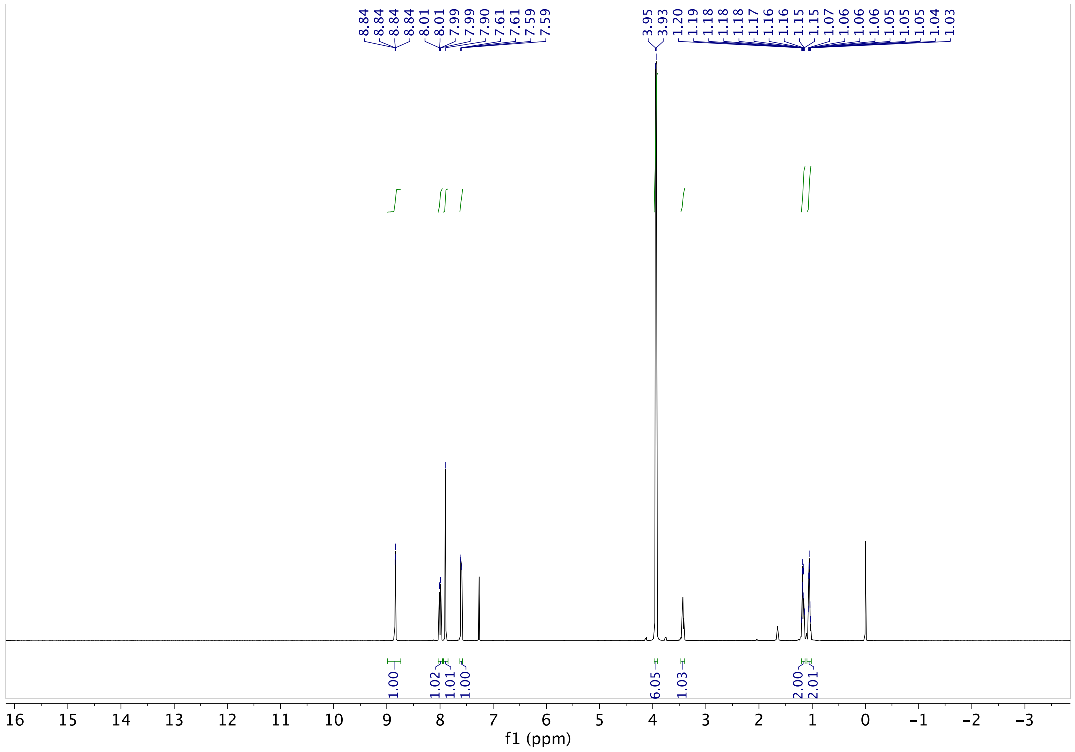

^{13}C NMR for dimethyl 1-cyclopropyl-1*H*-indole-3,5-dicarboxylate (**19d**)

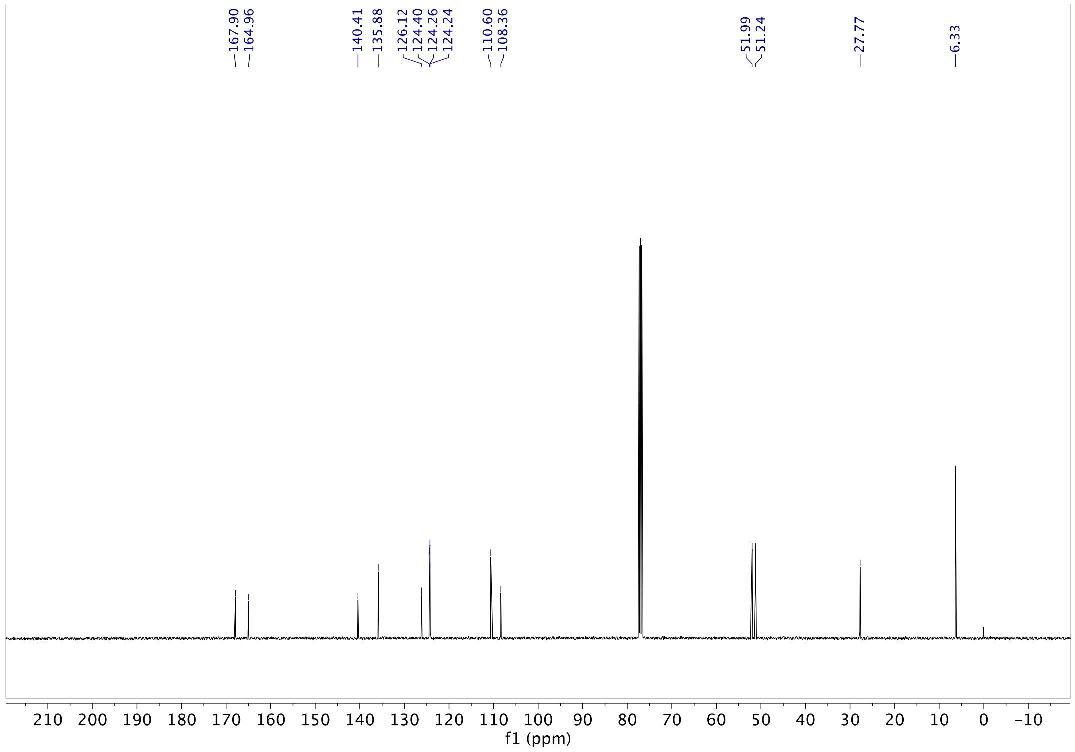

^{1}H NMR for dimethyl 1-cyclohexyl-1*H*-indole-3,5-dicarboxylate (**19e**)

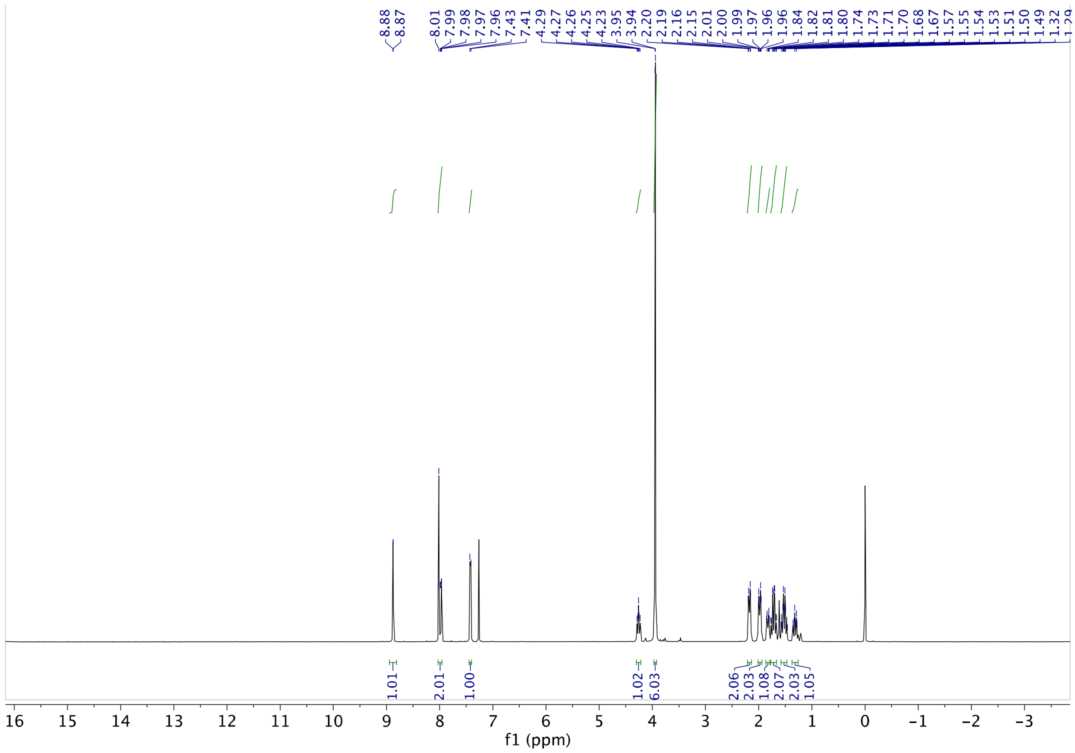

^{13}C NMR for dimethyl 1-cyclohexyl-1*H*-indole-3,5-dicarboxylate (**19e**)

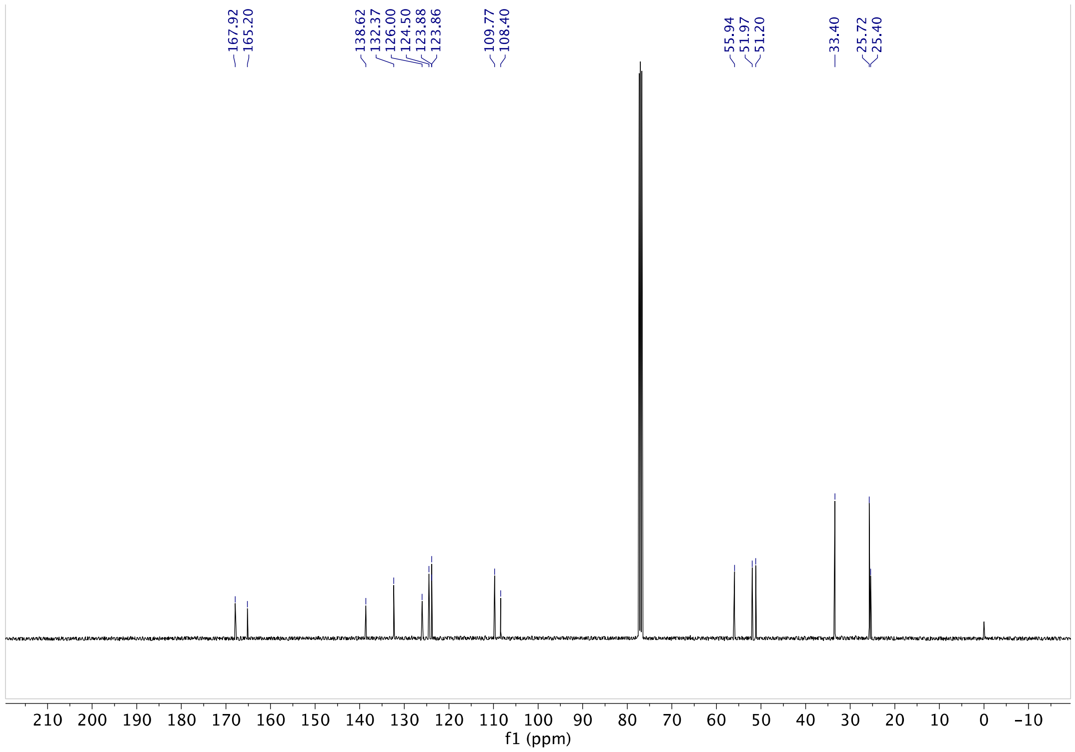

^{1}H NMR for dimethyl 1-phenethyl-1*H*-indole-3,5-dicarboxylate (**19g**)

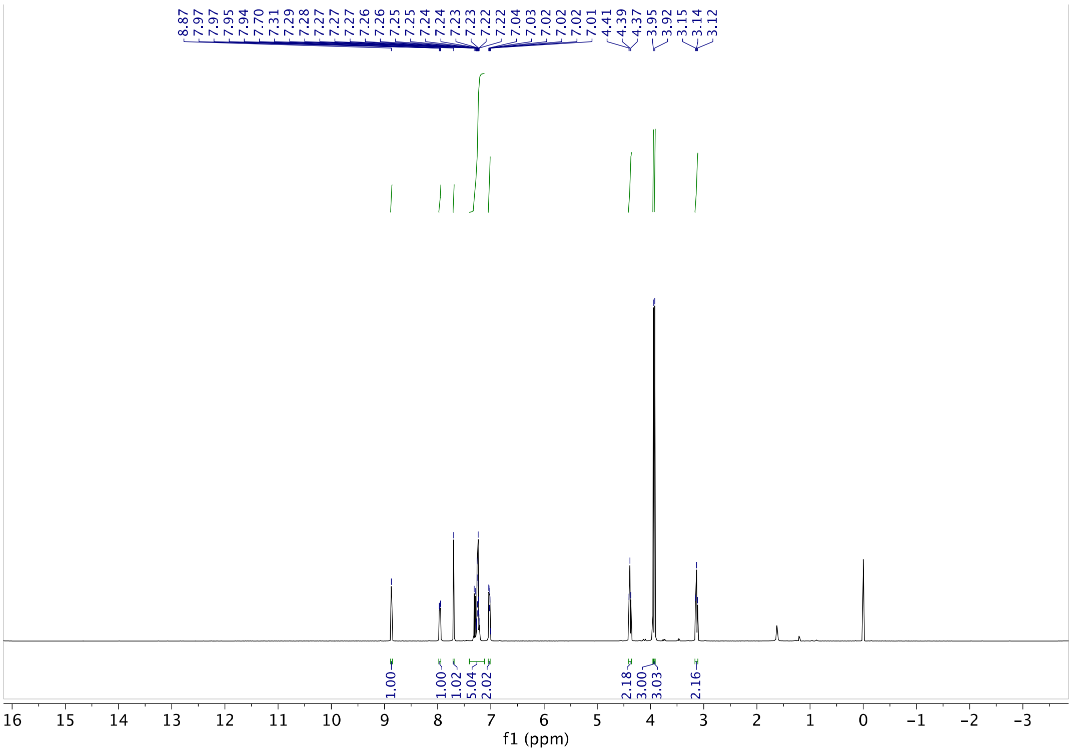

^{13}C NMR for dimethyl 1-phenethyl-1*H*-indole-3,5-dicarboxylate (**19g**)

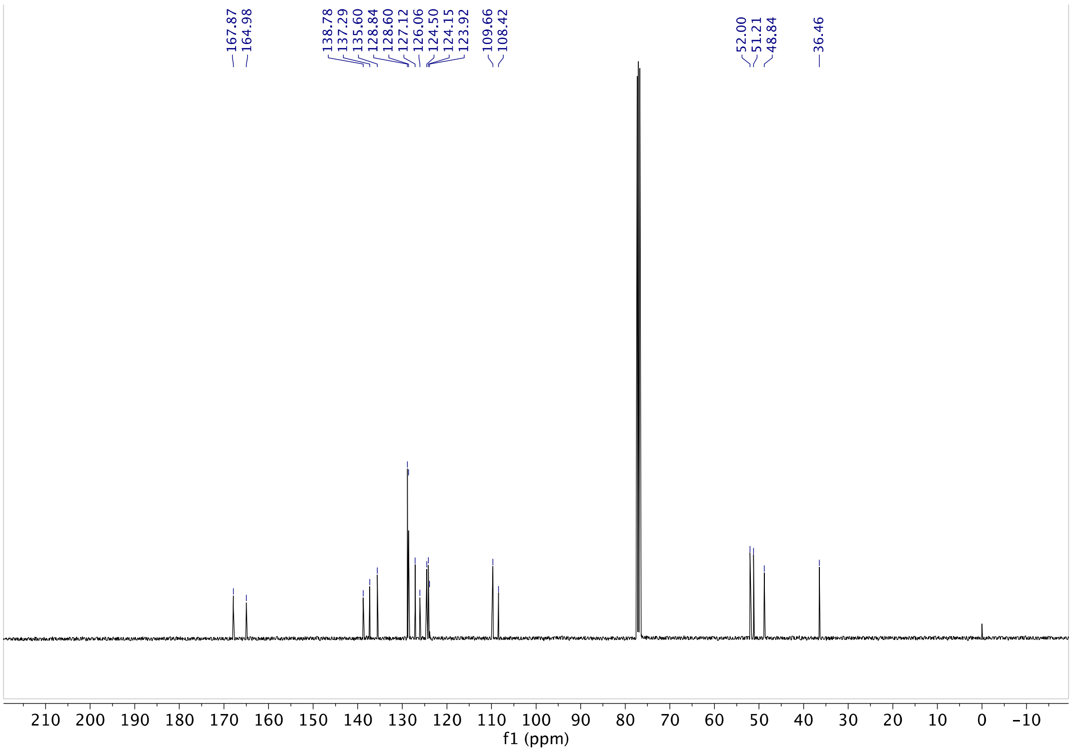

^{1}H NMR for dimethyl 1-benzyl-1*H*-indole-3,5-dicarboxylate (**19h**)

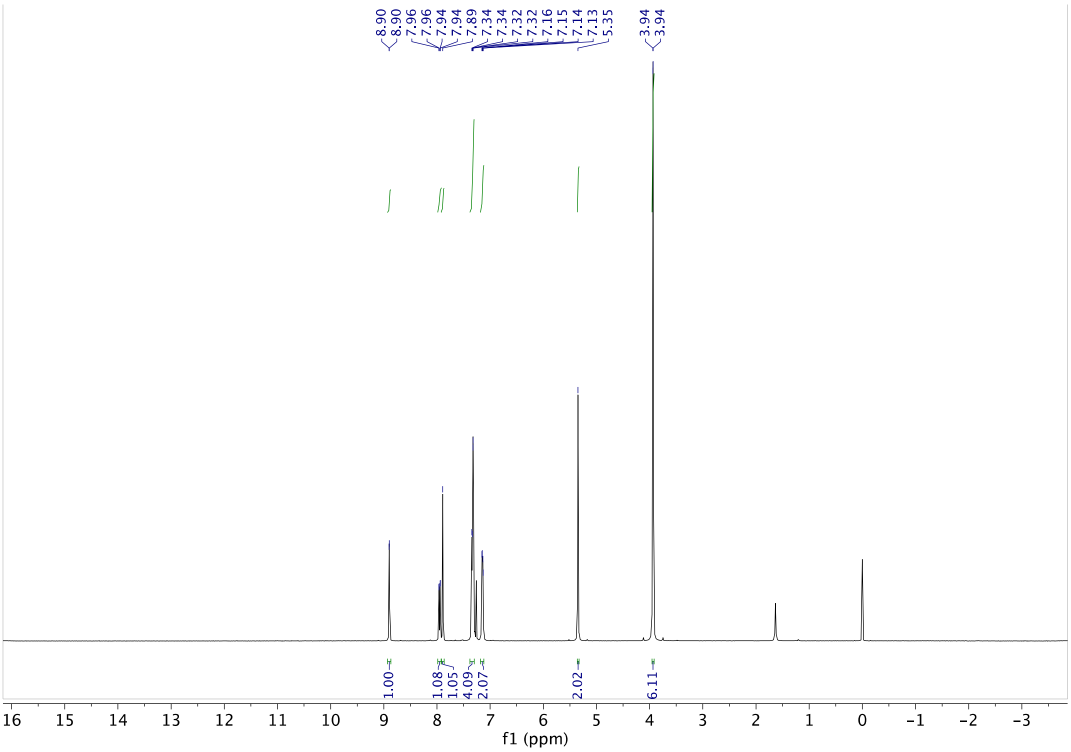

^{13}C NMR for dimethyl 1-benzyl-1*H*-indole-3,5-dicarboxylate (**19h**)

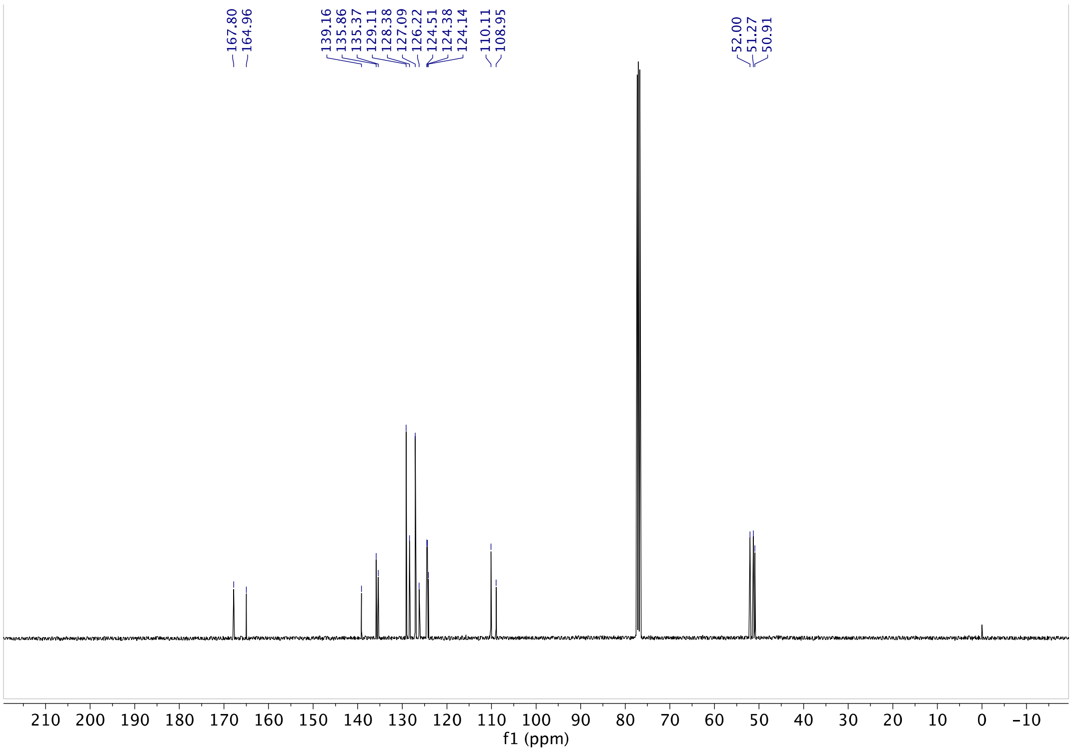

^{1}H NMR for dimethyl 1-(4-methylbenzyl)-1*H*-indole-3,5-dicarboxylate (**19i**)

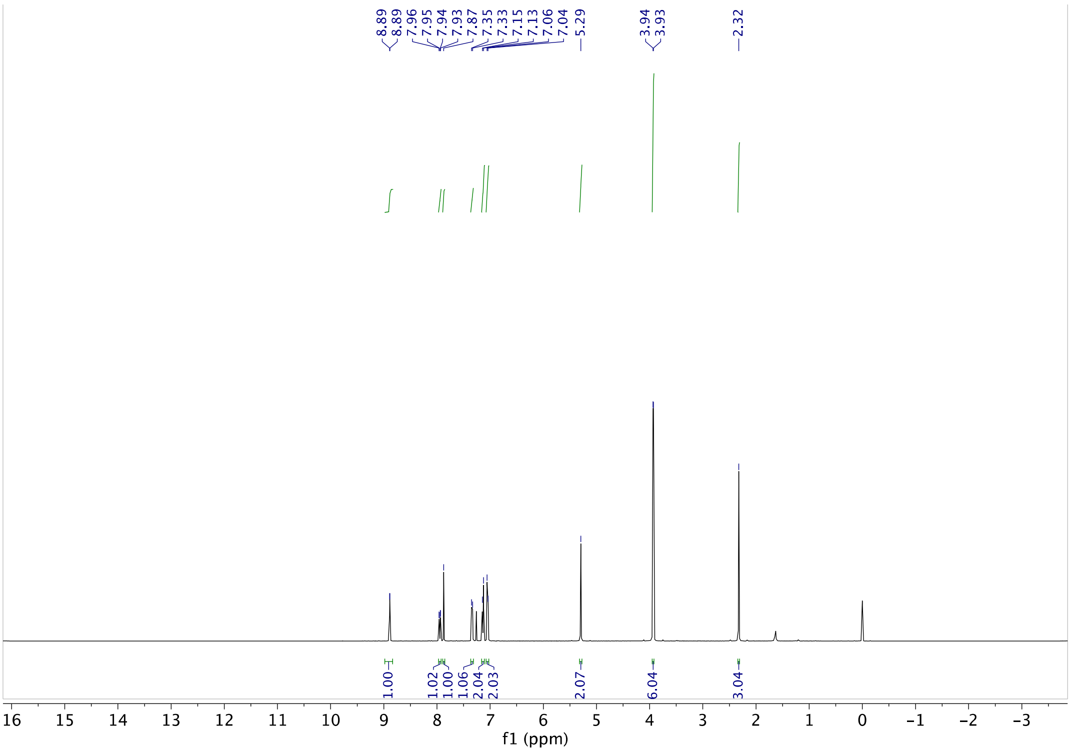

^{13}C NMR for dimethyl 1-(4-methylbenzyl)-1*H*-indole-3,5-dicarboxylate (**19i**)

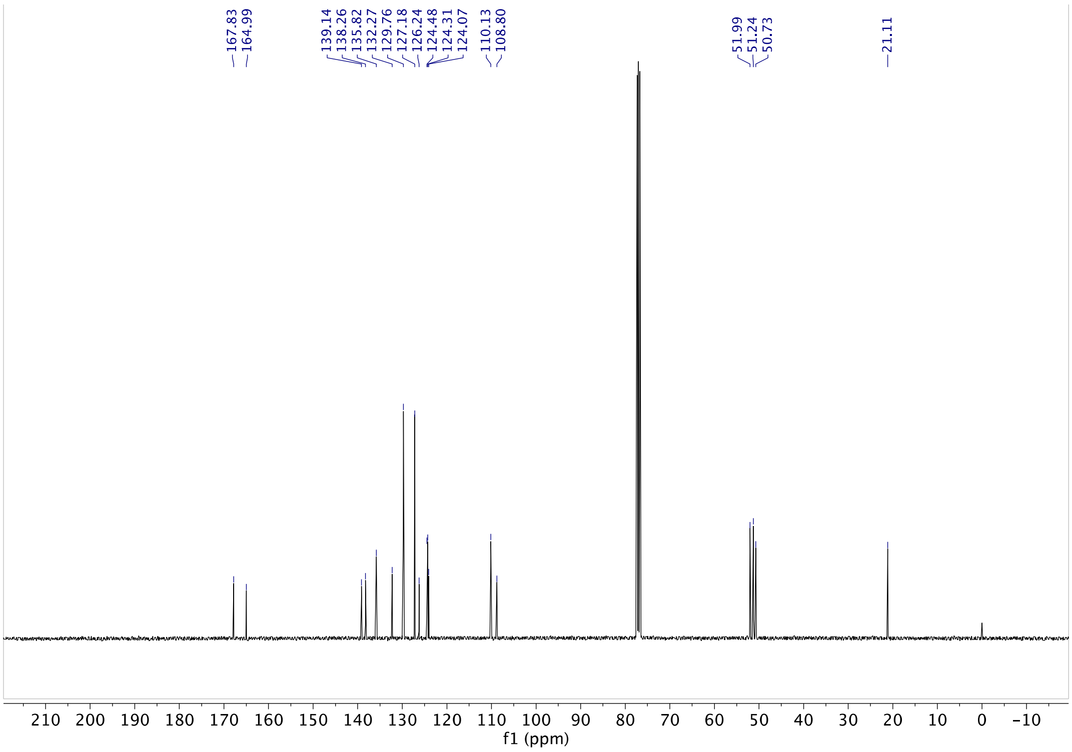

^{1}H NMR for dimethyl 1-(3-methoxybenzyl)-1*H*-indole-3,5-dicarboxylate (**19j**)

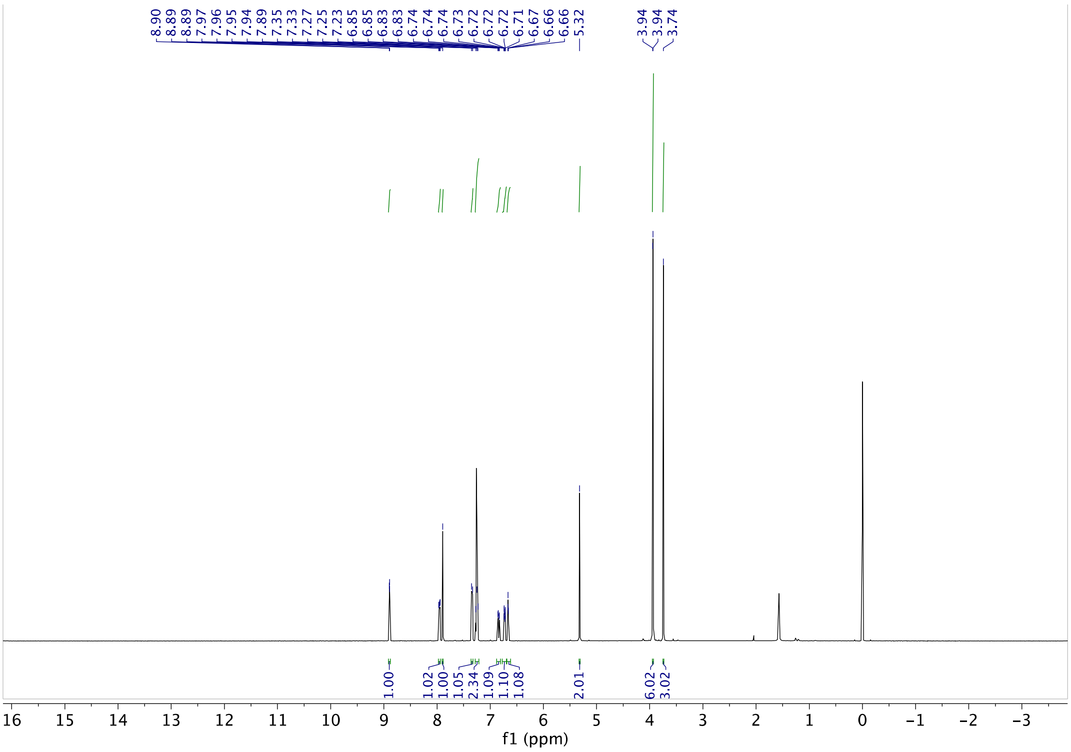

^{13}C NMR for dimethyl 1-(3-methoxybenzyl)-1*H*-indole-3,5-dicarboxylate (**19j**)

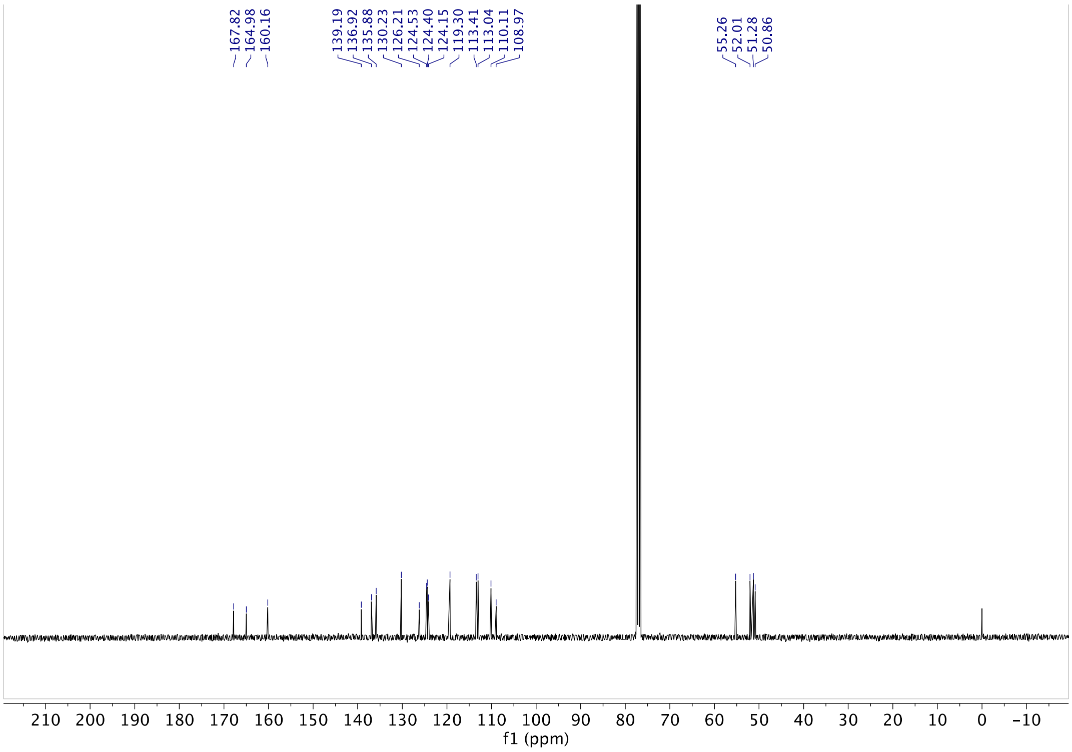

^{1}H NMR for dimethyl 1-(4-methoxybenzyl)-1*H*-indole-3,5-dicarboxylate (**19k**)

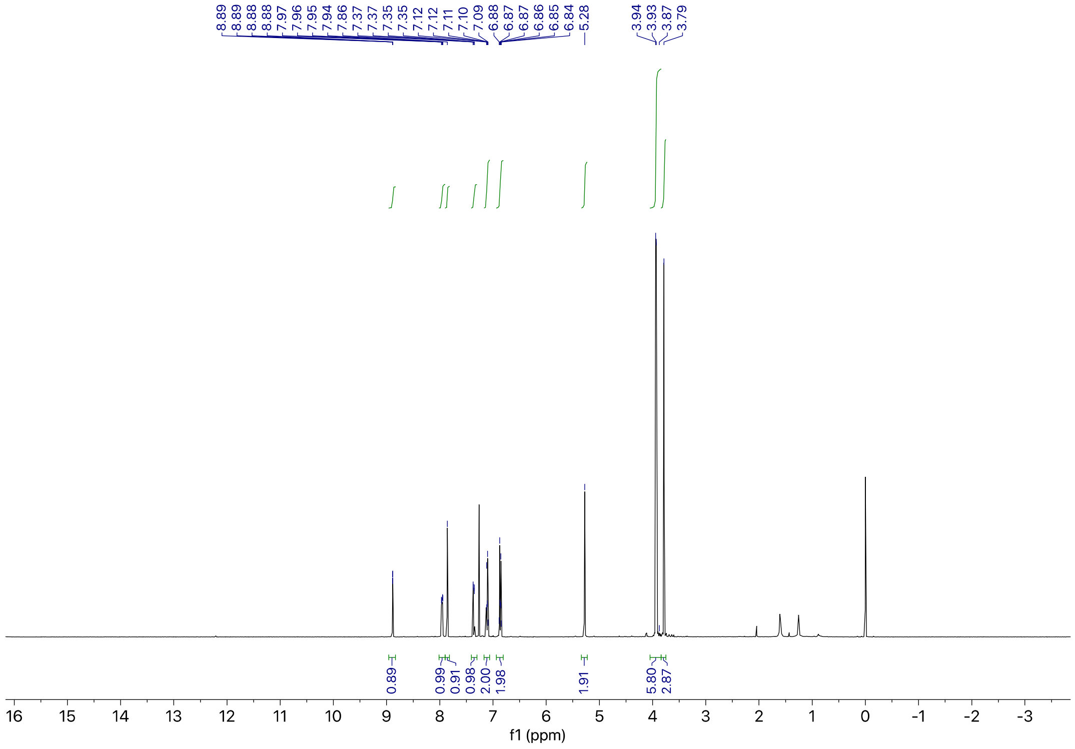

^{13}C NMR for dimethyl 1-(4-methoxybenzyl)-1*H*-indole-3,5-dicarboxylate (**19k**)

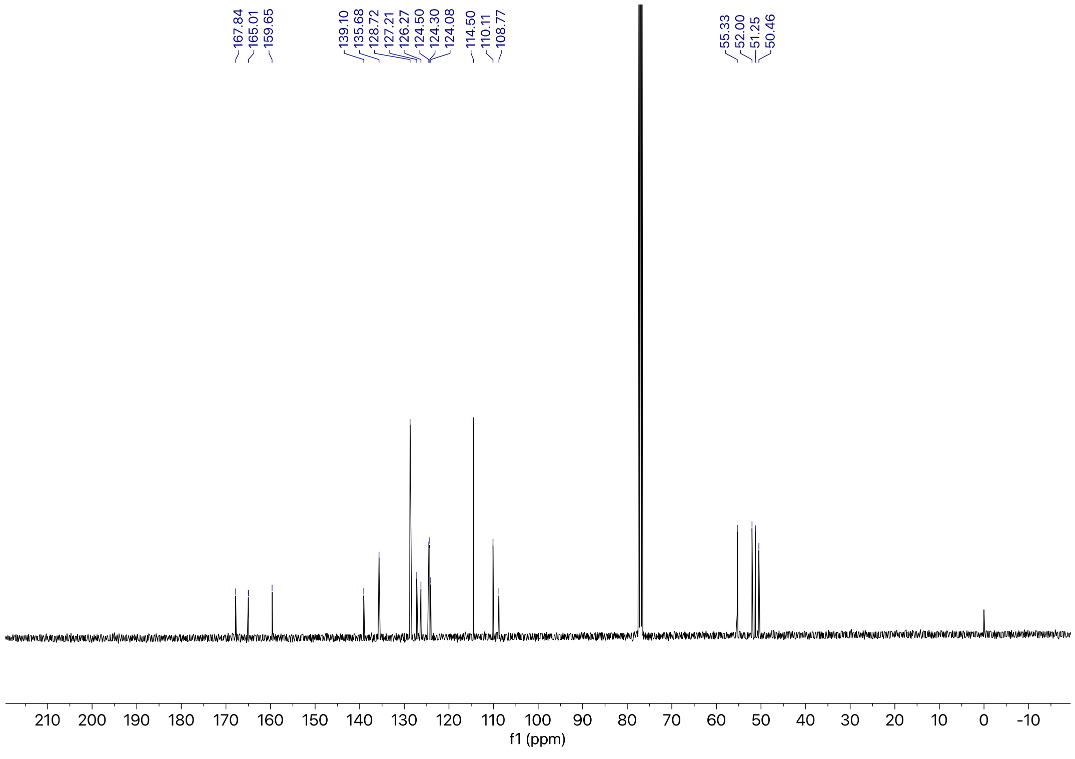

^{1}H NMR for dimethyl 1-(4-chlorobenzyl)-1*H*-indole-3,5-dicarboxylate (**19l**)

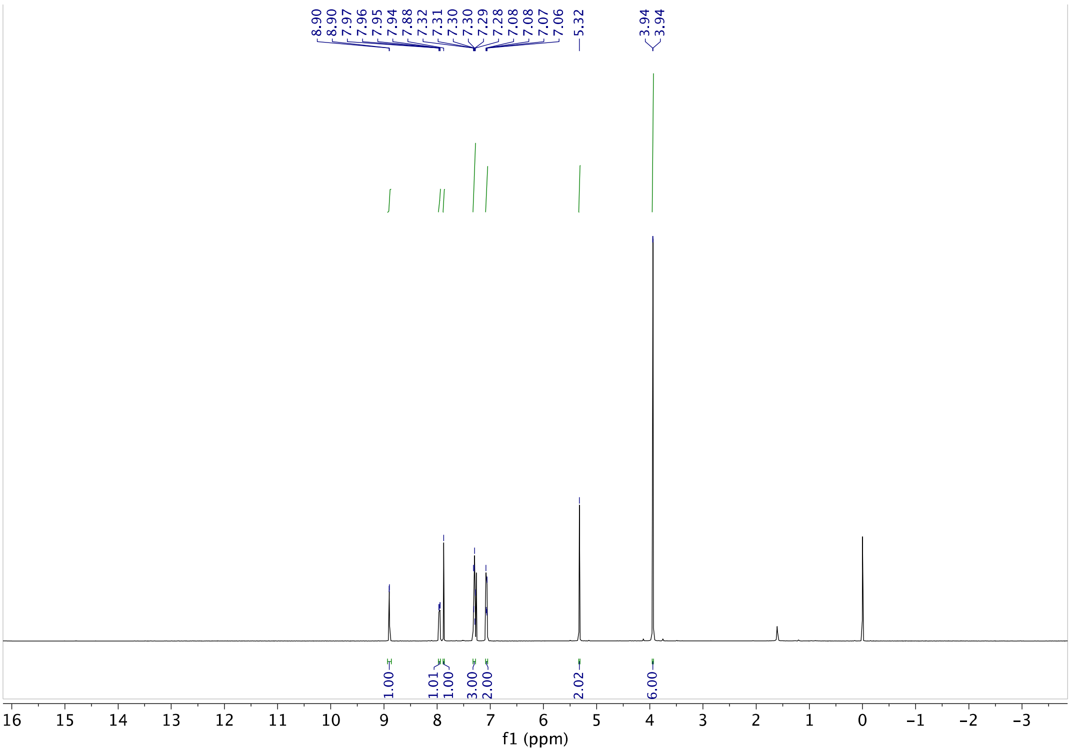

^{13}C NMR for dimethyl 1-(4-chlorobenzyl)-1*H*-indole-3,5-dicarboxylate (**19l**)

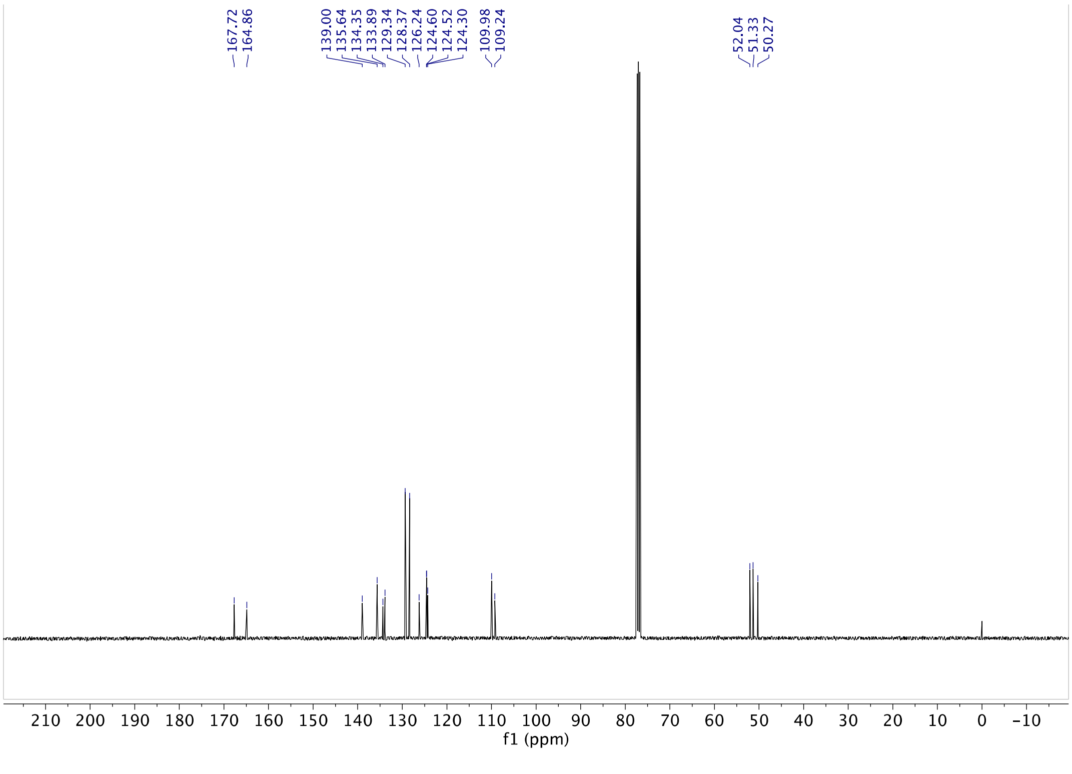

^{1}H NMR for dimethyl 1-(3-nitrobenzyl)-1*H*-indole-3,5-dicarboxylate (**19m**)

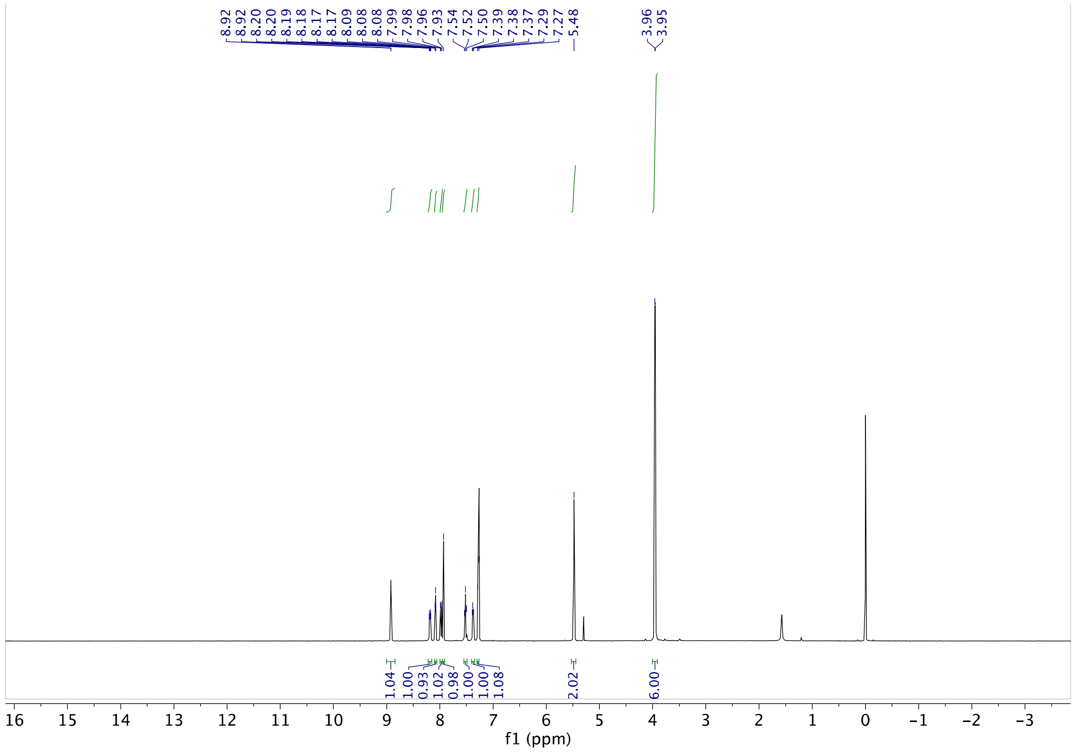

^{13}C NMR for dimethyl 1-(3-nitrobenzyl)-1*H*-indole-3,5-dicarboxylate (**19m**)

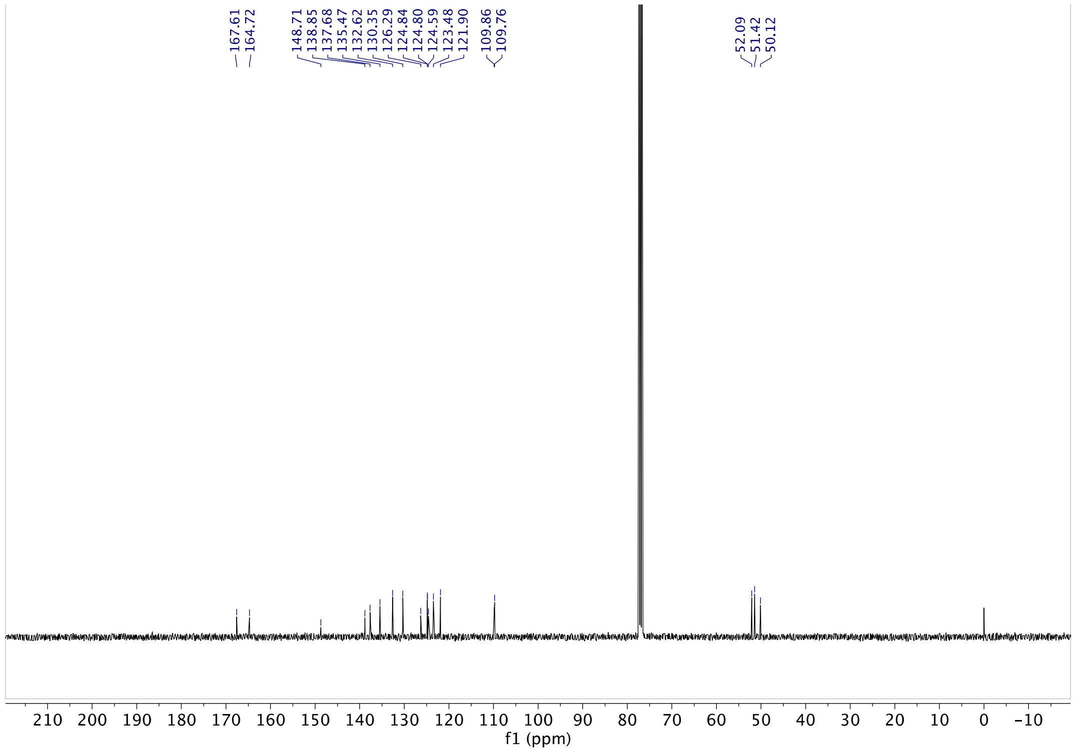

Supplement: Supplementary file 1 [file molecules-27-06998-s001.zip › molecules-1961713-supplementary.pdf]
